# Concerted aryl-sulfur reductive elimination from PNP pincer-supported Co(iii) and subsequent Co(i)/Co(iii) comproportionation†
†Electronic supplementary information (ESI) available: Experimental details and pictorial NMR spectra, details of the computational studies and the coordinate files. CCDC 1868266–1868268. For ESI and crystallographic data in CIF or other electronic format see DOI: 10.1039/d0sc01813a


**DOI:** 10.1039/d0sc01813a

**Published:** 2020-05-19

**Authors:** Bryan J. Foley, Chandra Mouli Palit, Nattamai Bhuvanesh, Jia Zhou, Oleg V. Ozerov

**Affiliations:** a Department of Chemistry , Texas A&M University , 3255 TAMU , College Station , Texas 77842 , USA . Email: ozerov@chem.tamu.edu; b School of Science , Harbin Institute of Technology , Shenzhen 518055 , China . Email: jiazhou@hit.edu.cn

## Abstract

This report discloses a combined experimental and computational study aimed at understanding C–S reductive elimination from Co(iii) supported by a diarylamido/bis(phosphine) PNP pincer ligand. Divalent (PNP)Co-aryl complexes could be easily oxidized to five-coordinate Co(iii) derivatives, and anion metathesis provided five-coordinate (PNP)Co(Ar)(SAr′) complexes of Co(iii). In contrast to their previously described (POCOP)Co(Ar)(SAr′) analogs, but similarly to the (PNP)Rh(Ar)(SAr′) and (POCOP)Rh(Ar)(SAr′) analogs, (PNP)Co(Ar)(SAr′) undergo C–S reductive elimination with the formation of the desired diarylsulfide product ArSAr′. DFT studies and experimental observations are consistent with a concerted process. However, in contrast to the Rh analogs, the immediate product of such reductive elimination, the unobserved Co(i) complex (PNP)Co, un-dergoes rapid comproportionation with the (PNP)Co(Ar)(SAr′) starting material to give Co(ii) compounds (PNP)Co–Ar and (PNP)Co-SAr′.

## Abstract 2

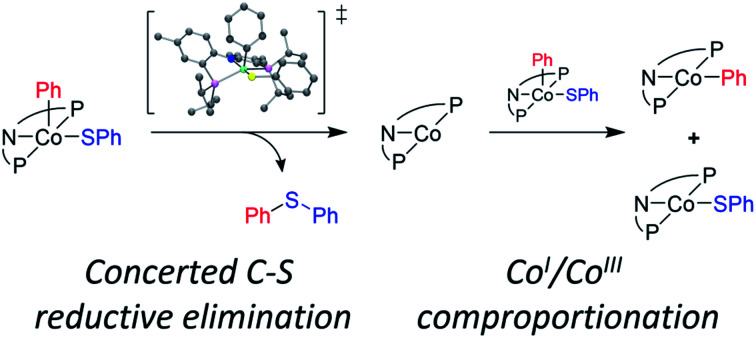
This report discloses a combined experimental and computational study aimed at understanding C–S reductive elimination from Co(iii) supported by a diarylamido/bis(phosphine) PNP pincer ligand.

## Introduction

Carbon-heteroatom cross coupling has become an immensely powerful synthetic tool in recent years.^1^–^4^ The existing art on cross-coupling reactions is historically dominated by palladium,^5^ with additional prominence by another group 10 metal Ni,^6^,^7^ as well as a group 11 metal Cu.^8^,^9^ There has recently been a renewed push to find alternatives to homogeneous precious metal catalysts from among cheaper, more Earth-abundant metals.^10^–^12^

**Fig. 1:**
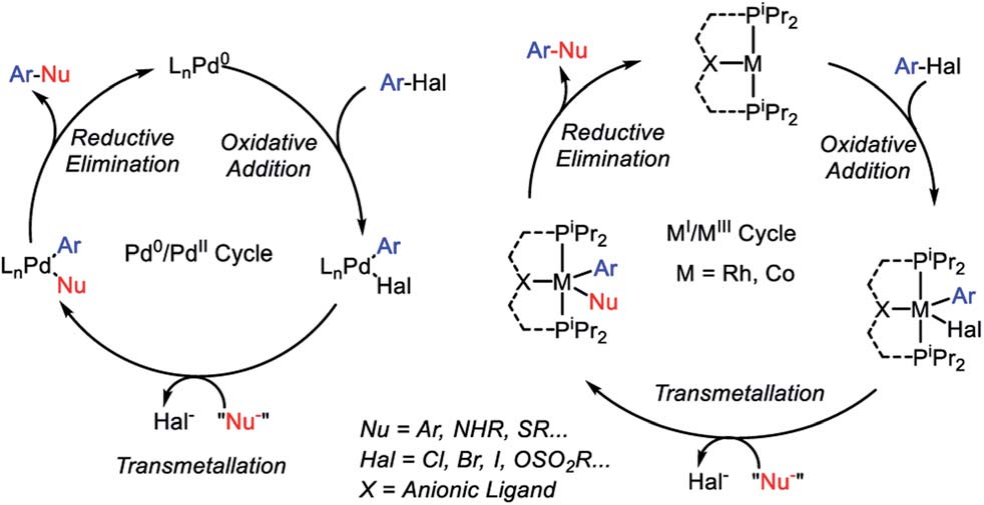
Cross-coupling mechanistic cycles for Pd^0^/Pd^II^ (left) and for Rh^I^/Rh^III^ or Co^I^/Co^III^ (right).

Cross-coupling reactions of aryl (pseudo)halides with nucleophiles typically rely on the oxidative addition (OA) – transmetallation (TM) – reductive elimination (RE) cycles such as depicted in Fig. 1. The OA and RE steps are two-electron processes that are well established for Pd. Our group has been interested in the potential of the analogous OA–TM–RE cycle to enable cross-coupling catalysis by group 9 metals. Pd (and Ni) go through the Pd^0^/Pd^II^ oxidation states corresponding to the d^10^/d^8^ configurations. For group 9 metals, we have targeted the M^I^/M^III^ oxidation states (d^8^/d^6^). In particular, we were able to establish that a T-shaped Rh^I^ center supported by an anionic pincer ligand possesses a rather striking similarity in its reactivity to the LPd^0^ fragment.^13^–^18^ This approach with Rh proved especially fruitful in catalytic C–S coupling.^19^ Catalytic C–S coupling with Pd has received a considerable amount of attention.^20^,^21^

It is easy to envisage the steps of the analogous pincer-supported Co^I/III^ cycle (Fig. 1). However, in the chemistry of 3d metals, competition from one-electron pathways to the desired two-electron steps is something that must be closely considered.^22^ In principle, there is a substantial body of literature describing Co-based cross-coupling catalysis,^23^ and Co^I^/Co^III^ cycles are often proposed.^24^–^28^ However, firm mechanistic information remains rather limited. Fout and coworkers analyzed a Co-catalyzed aryl halide amination system in 2014 (ref. 29) where the Co^I^/Co^III^ cycle was strongly implicated but the individual steps of OA and RE were not observed. Chirik *et al.* reported on the C–C coupling of aryl triflates in 2016, where it appears that the Co^I^/Co^III^ cycle should operate but the details were not uncovered.^30^ Bernskoetter's group reported a well-defined example of C–C RE from Co^III^ in 2011,^31^ but this involved coupling of two CH_3_ groups, only indirectly related to aryl halide reactions. However, outside of aryl halide coupling reactions, there have in recent years appeared examples of homogeneous catalysis by pincer-supported Co complexes where two-electron OA/RE steps are either well understood or strongly suggested.^32^–^42^

**Scheme 1:**
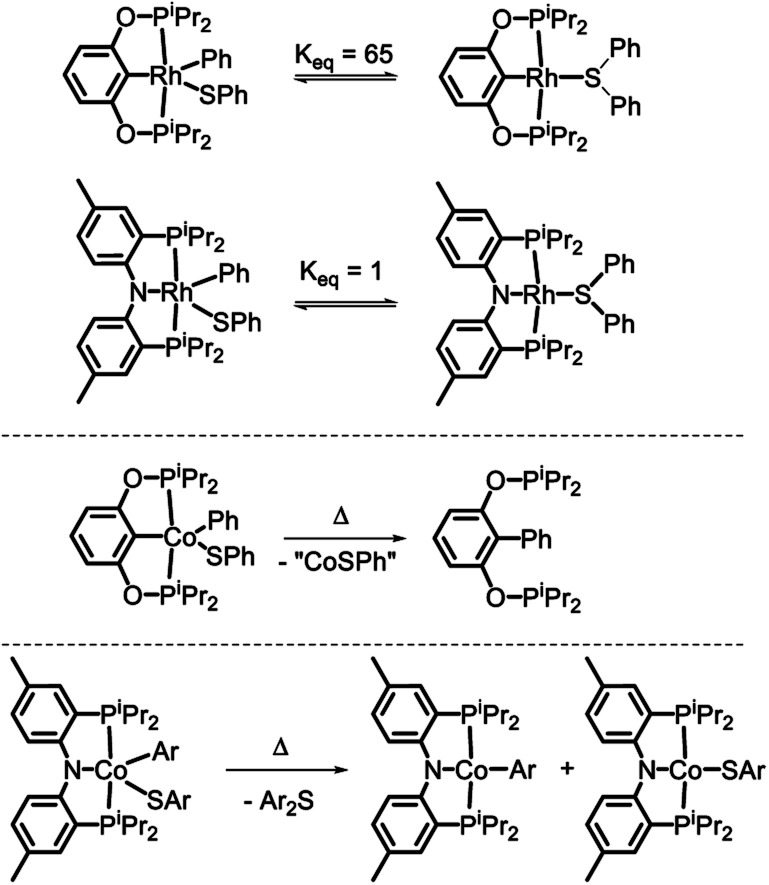
C–S reductive coupling observed for the (POCOP)Rh(Ph)(SPh) and (PNP)Rh(Ph)(SPh) complexes (ref. 18 and 19, top), C–C coupling with the pincer carbon in (POCOP)Co(Ph)(SPh) (ref. 43, middle), and the subject of this work (bottom).

In 2018, we reported on the reactivity of (POCOP)Co(Ph)(SPh).^43^ In contrast to (PNP)Rh(Ph)(SPh)^18^ or (POCOP)Rh(Ph)(SPh),^19^ it did not undergo C–S RE but instead a RE of the phenyl with the pincer aryl (Scheme 1). Because of this, the POCOP system did not allow for the investigation of the C–S RE. We surmised that the analogous RE with the amido of a PNP pincer should be less likely and set off to examine the reactivity of (PNP)Co(Ar)(SAr) complexes. The present report details our efforts in the synthesis of five-coordinate Co^III^-aryl/thiolate complexes supported by the PNP ligand, their propensity to undergo concerted C–S RE, and the subsequent comproportionation reactivity that again diverges from the Rh system.

## Results and discussion

### Synthesis and characterization of (PNP)Co^II^ complexes

**Scheme 2:**
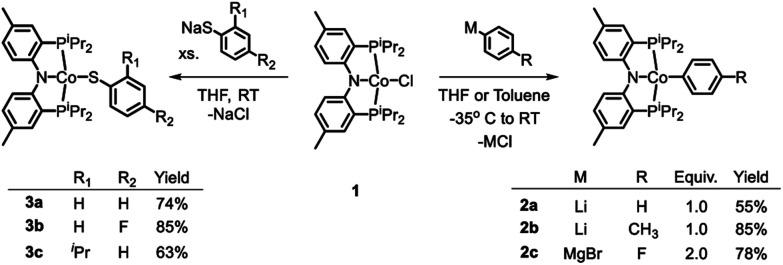
Transmetallation reactions among (PNP)Co^II^X.

Treatment of the previously reported square planar, low spin, *S* = 1/2 (PNP)CoCl^44^ (**1**) with selected aryl nucleophiles resulted in the formation of the corresponding Co^II^ aryl complexes **2a–c** (Scheme 2). Clean transmetallation of **1** was also accomplished using sodium thiophenolate reagents to give Co(ii) thiolate complexes **3a–c** (Scheme 2). Complexes **2a–c** and **3a–c** were green to dark teal in color. They exhibited paramagnetically shifted ^1^H NMR resonances contained in the +40 to –30 ppm range, except for the resonances at around –90 ppm in complexes **2a–2c** which we tentatively assign as *ortho*-hydrogens of the Co-bound aryl rings. No ^31^P NMR resonances were detected for these complexes.

**Fig. 2:**
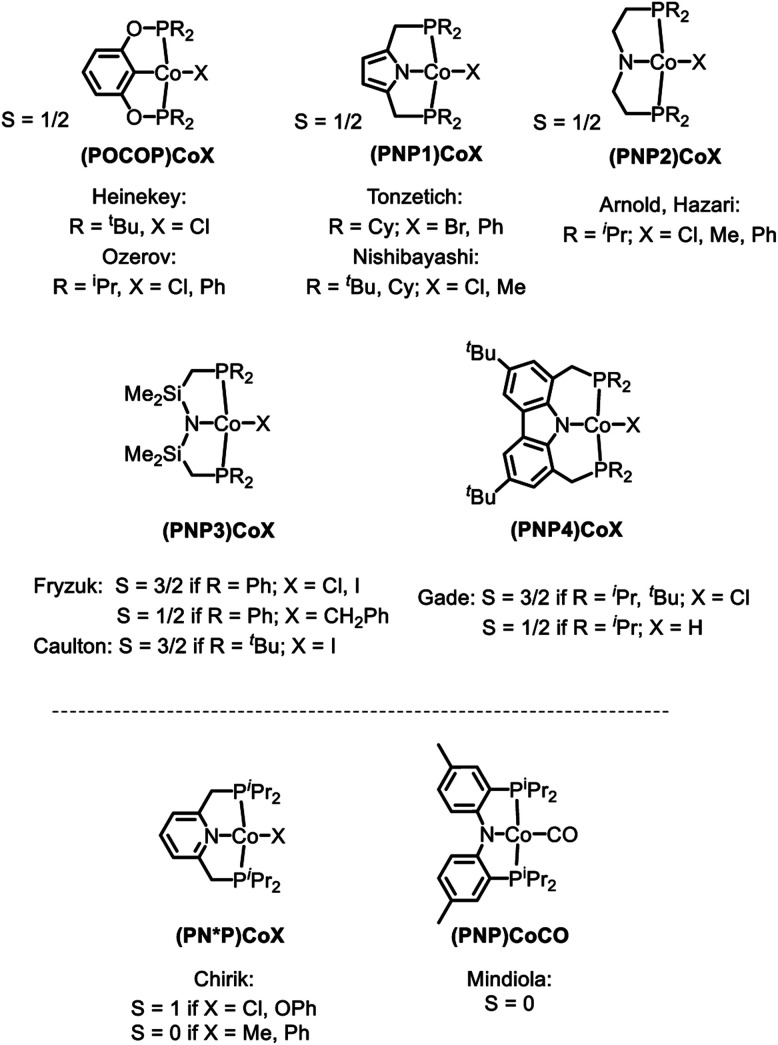
High and low spin dichotomy in POCOP and PNP pincer-supported four-coordinate complexes of Co^II^ (top) and Co^I^ (bottom).

Examination of the literature shows that four-coordinate Co(ii) complexes of anionic pincer ligands are known with both a low-spin *S* = 1/2 configuration (square-planar geometry) and a high-spin *S* = 3/2 configuration (pseudotetrahedral geometry).^43^–^52^ Low-spin, square-planar Co(ii) complexes give rise to paramagnetically shifted ^1^H NMR resonances that are broad compared to diamagnetic compounds, but are typically interpretable in terms of their relative integration and chemical (in)equivalence. High-spin Co(ii) compounds tend to produce ^1^H NMR spectra that are broadened beyond useful interpretation. Complexes **2a–c** and **3a–c** in the present work and the (POCOP)CoX complexes (Fig. 2) recently reported by us and Heinekey *et al.*^45^ are all low-spin compounds. The same is true for the (PNP1)CoX complexes of the pyrrolyl-based PNP ligand by Tonzetich *et al.*^46^ and Nishibayashi *et al.*,^47^ as well as the (PNP2)CoX complexes reported by Arnold *et al.* and Hazari *et al.*^48^,^49^ In contrast, Co(ii) halide complexes of the Fryzuk-type PNP3 and Gade's carbazole-based PNP4 ligands are high-spin.^50^–^52^ On the other hand, (PNP3)Co(CH_2_Ph) and (PNP4)CoH are low spin.^50^,^52^ It appears that the presence of even one very strong-field ligand such as hydride or aryl/alkyl is sufficient for the low-spin preference. It is interesting that in their absence, the various Co^II^ complexes in Fig. 2 contain low and high-spin complexes with essentially the same set of donors (*e.g.*, high-spin (PNP3)CoCl *vs.* low-spin (PNP)CoCl (**1**) or (PNP1)CoBr or (PNP2)CoCl). All the complexes in Fig. 2 possess *trans*-disposed phosphines or phosphinites in the pincer, and it does not appear that their presence alone is sufficient to ensure low-spin configurations. We surmise that the geometric constraint of the ligand plays a role in enforcing the corresponding geometry and thus spin state, with the less flexible PNP and PNP1 favoring square planar, low-spin Co^II^.^53^ The PNP4 ligand contains the most π-donating (dialkylamido) central N donor of the selection in Fig. 2, which may help stabilize a low-spin configuration. Amido/bis(phosphine) PNP pincer ligands can be oxidized at the ligand and thus are potentially redox non-innocent,^54^ but we see no evidence of it in this work.

### Synthesis and characterization of (PNP)Co^III^(Ar)(X) complexes

**Scheme 3:**
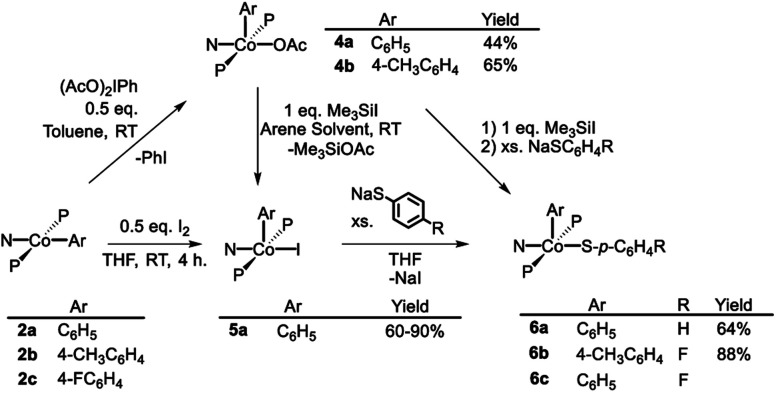
Oxidation of (PNP)Co^II^ and synthesis of target (PNP)Co(Ar)(S–Ar).

As is the case in our (POCOP)Co system,^43^ reacting (PNP)Co(Aryl) complexes **2a–b** with 0.5 eq. of PhI(OAc)_2_ led to the clean formation of (PNP)Co(Aryl)(OAc) (**4a–b**), isolated in good yield as tan solids after workup (Scheme 3). Treatment of **4a** with Me_3_SiI furnished dark blue-green (PNP)Co(Ph)(I) (**5a**). **5a** can also be prepared *via* reaction of **2a** with 0.5 equiv. of I_2_. Transmetallation from **5a** using sodium thiophenolate proceeded smoothly providing (PNP)Co(Ph)(SPh) complex **6a** as a dark blue solid in good yields after recrystallization (Scheme 3).^55^ The analogous synthesis of (PNP)Co(Ar)(SAr′) complexes **6b–c** from **4b–c** can be achieved without the isolation of the intermediate **5b–c**.

The (PNP)Co(Ar)(X) compounds (**4a–b**, **5a**, and **6a–c**) gave rise to ^1^H, ^31^P, and ^13^C NMR spectra expected for these diamagnetic complexes. The resonances arising from the Co-bound aryl group exhibited inequivalence between the two *ortho*- and between the two *meta*-hydrogens, characteristic of restricted rotation of the metal-bound aryl oriented *cis* to the central donor of a pincer ligand with two side –P^i^Pr_2_ arms.^14^,^43^,^56^ In the cases of the aryl/-thiolate complexes **6a–6c**, these aromatic resonances were broad humps, whereas in the aryl/-halide **5a** and aryl/-acetato complexes **4a–4b**, sharp resonances with well-resolved fine structure were observed.

### Synthesis and characterization of (PNP)Co^I^ complexes

**Scheme 4:**
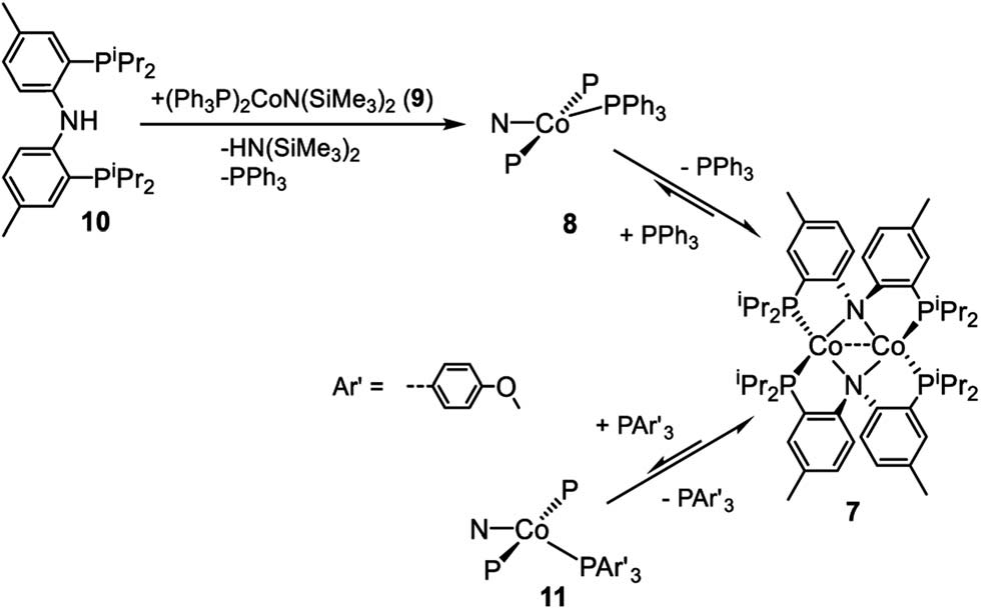
Formation of proposed (PNP)Co(PPh_3_) and equilibrium between (PNP)_2_Co_2_ and (PNP)Co(P(Ar)_3_).

The dimeric compound [(PNP)Co]_2_ (**7**) (Scheme 4) was previously reported by Mindiola *et al.*^44^ We were also able to observe a Co^I^ complex (PNP)Co(PPh_3_) (**8**) by treatment of (Ph_3_P)_2_CoN(SiMe_3_)_2_ (**9**)^29^ with (PNP)H (**10**). This reaction liberated triphenyl phosphine and HN(SiMe_3_)_2_ (Scheme 4). A wide ^1^H NMR spectral window revealed a new set of paramagnetically shifted ^1^H NMR resonances which we have assigned to **8**. Compound **8** was not isolated as it appears to be in equilibrium with diamagnetic **7** on the timescale of experimental handling. For example, freshly made **8** was observed to produce **7** when left overnight in a –35 °C freezer, while addition of 12 equiv. of PPh_3_ to **7** led to the observation of **8** (Scheme 4). Addition of 12 eq. of tris(4-methoxyphenyl)phosphine to this mixture gave rise to a second set of distinct but very similar paramagnetically shifted ^1^H NMR resonances we interpret as belonging to **11** (Scheme 4, Fig. S12†). This observation supports the notion that **8** is a PPh_3_-bound Co complex.

Based on the paramagnetically shifted ^1^H NMR spectra, we assume that both **8** and **11** possess an *S* = 1 ground state. As with four-coordinate Co(ii), there are examples of both high-spin and low-spin pincer complexes of Co(i) (*S* = 1 or 0) (Fig. 2, bottom). With the pyridine-centered PN*P ligand, complexes substituted with stronger field Me or Ph are low-spin, while Cl or OAr as the fourth donor are high-spin.^30^,^57^ With the PNP ligand, CO in place of PPh_3_ in **8** was reported to give a low-spin carbonyl complex (PNP)Co(CO). Thus, it appears that the presence of at least one strong-field ligand (CO, or hydride/alkyl/aryl) is needed to ensure an *S* = 0 ground state.

### X-ray structural studies

**Fig. 3:**
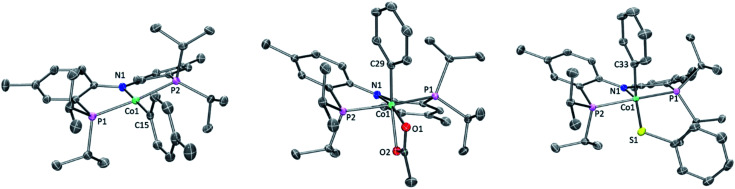
POV-ray renditions of ORTEP drawings (50% probability ellipsoids) of (PNP)Co(Tol), (PNP)Co(Ph)(OAc), and (PNP)Co(Ph)(SPh). All hydrogen atoms omitted. Selected bond distances (Å) and angles (degrees) for (PNP)Co(Tol) (**2b**, left): N1–Co1, 1.9262(17); P1–Co1, 2.1756(7); P2–Co1, 2.1849(7); C15–Co1, 1.939(2); N1–Co1–C15, 178.99(9); P1–Co1–P2, 172.63(3). Selected bond distances (Å) and angles (degrees) for (PNP)Co(Ph)(OAc) (**4a**, middle): N1–Co1, 1.9333(12); O1–Co1, 1.9896(12); O2–Co1, 2.1166(11); P1–Co1, 2.2619(7); P2–Co1, 2.2353(6); C29–Co1, 1.9403(15); C29–Co1–N1, 97.59(6); N1–Co1–O1, 165.17(5). Selected bond distances (Å) and angles (degrees) for (PNP)Co(Ph)(SPh) (**6a**, right): N1–Co1, 1.9497(18); P1–Co1, 2.2597(7); P2–Co1, 2.2313(7); C33–Co1, 1.933(2); S1–Co1, 2.2069(6); N1–Co1–C33, 98.54(8); N1–Co1–S1, 149.60(6).

Single crystals of **2b**, **4a**, and **6a** suitable for X-ray diffraction were grown from hydrocarbon solvents at –35 °C (Fig. 3). The geometry about the cobalt center in the solid-state structure of **2b** is slightly distorted square planar. The Co-bound tolyl ring in **2b** is approximately perpendicular to the Co/P/N/P plane.

The structure of **4a** is pseudo-octahedral about the metal center, with a κ^2^ acetate coordination. The two oxygens of the acetate are bound *trans* to two donors of markedly different *trans*-influence (amido N *vs.* phenyl C), which is reflected in the large difference between the two Co–O bond distances (*ca.* 0.13 Å). The geometry about Co in **6a** is intermediate between square-pyramidal with the phenyl *trans* to the empty site and Y-shaped (with the thiolate at the base of the Y). The preference of low-spin five-coordinate d^6^ complexes for square-pyramidal and Y-shaped geometries have been discussed elsewhere.^58^ The angles, bond lengths, and orientation of the thioaryl ligand about the cobalt center for **6a** are similar to those that we reported for (POCOP)Co(Ph)(SPh).^43^

### Thermolysis of (PNP)Co^III^(Ar)(SAr) complexes

**Scheme 5:**
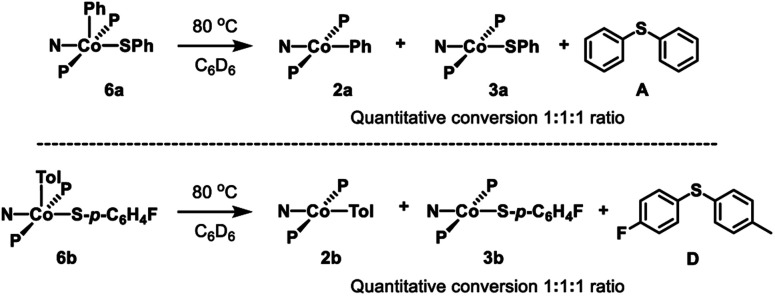
Top: Thermolysis of **6a** and observed products. Bottom: Thermolysis of **6b** and observed products.

Thermolysis of **6a** in benzene led to the formation of **2a**, **3a**, and **A** in a 1 : 1 : 1 ratio (Scheme 5, top). Further investigation showed that this process is first order in **6a** (Fig. S1†). Thermolysis of this complex in the presence of 1 eq. of 2,6-di-*tert*-butyl-4-methylphenol (BHT) resulted in the same distribution of products in the same time period, providing evidence against generation of free aryl radicals. Similarly, thermolysis of **6b** in benzene resulted in the formation of **2b**, **3b**, and **C** in a 1 : 1 : 1 ratio (Scheme 5, bottom; Fig. S3 and S4†).

**Scheme 6:**
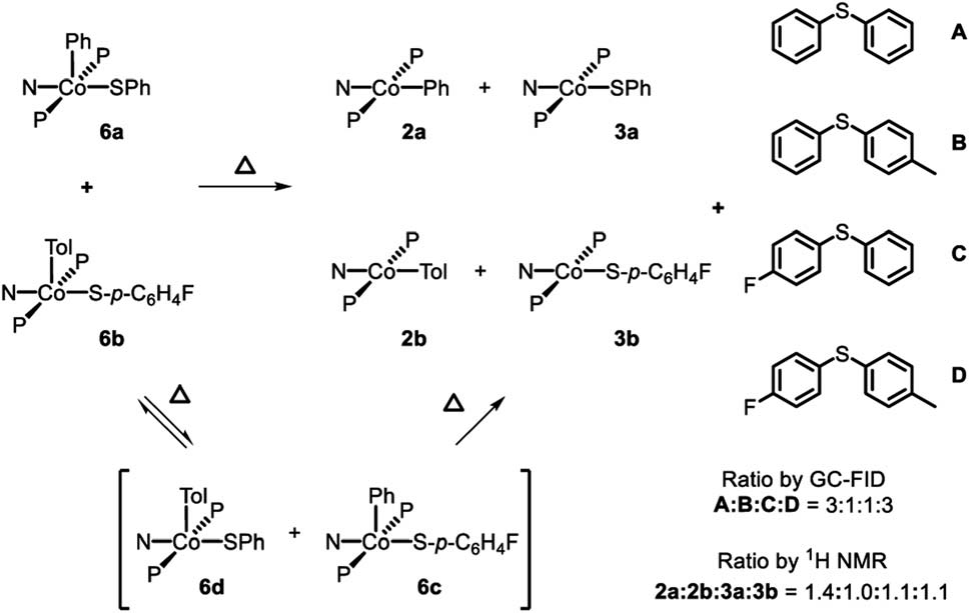
Thermolysis of a mixture of **6a** and **6b**.

Attempting to determine whether the C–S bond formation step happened at a single Co center, thermolysis of **6a** in a 1 : 1 ratio with **6b** was carried out. In principle, strict unimolecular C–S reductive elimination should lead to only two diarylsulfide products as a result of this thermolysis. In the event, formation of four diarylsulfides was instead observed, along with the four expected Co^II^ products: **2a**, **2b**, **3a**, and **3b** (Scheme 6). ^19^F NMR analysis during the course of the reaction at 80 °C revealed the formation of the Co(iii) crossover product **6c** (Fig. S5†). This suggested that during the thermolysis, thiolate ligands can exchange between the Co(iii) centers prior to RE. This exchange would then lead to the formation of crossover diarylsulfides, even if RE happens unimolecularly, and thus prevent us from firmly excluding crossover *via* other pathways. Performing this reaction at double the initial concentration of Co^III^ complexes still showed **6c** during thermolysis and a very similar distribution of Co^II^ products and diaryl sulfides after the reaction had completed.

**Scheme 7:**
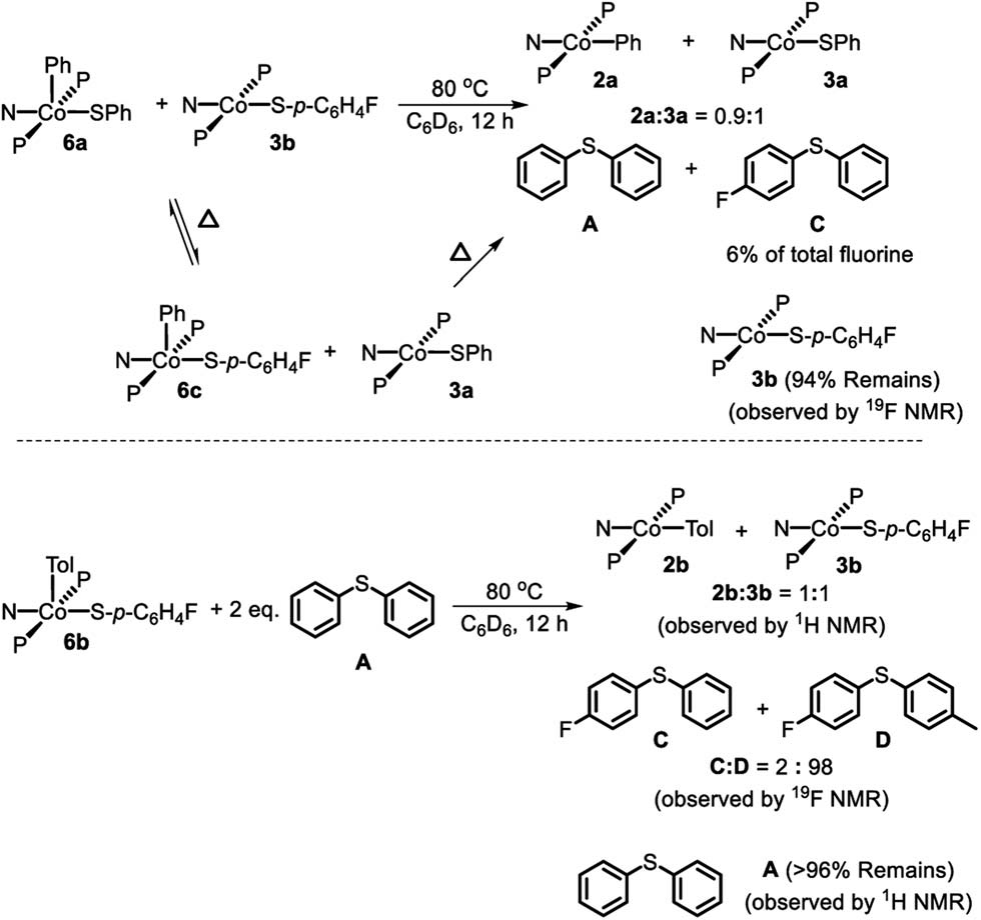
Top: Thermolysis of (PNP)Co(Ph)(SPh) (**6a**) in the presence of (PNP)Co(S-*p*-C_6_H_4_F) (**3b**) and observed products. Bottom: Thermolysis of **6b** in the presence of **A** and observed products.

The thiolate exchange between Co(iii) complexes could occur in at least^59^ two non-exclusive ways: (1) direct thiolate exchange between two Co(iii) complexes, (2) *via* exchange between Co(ii) and Co(iii) thiolate complexes. To probe the ability of Co(iii) to exchange thiolate ligands with Co(ii), **6a** was thermolyzed in the presence of 1 eq. **3b**. *In situ*^19^F NMR observation at 80 °C revealed the formation **6c** during the reaction (Scheme 7, top). After the thermolysis was complete, 6% of the total starting fluorinated thiolate was found as **C** demonstrating that Co^II^ and Co^III^ can swap thiolates. The conditions required for the observation of thiolate swapping between two Co(iii) complexes inevitably led to at least some Co(ii) thiolate, which prevented us from establishing whether Co(iii) thiolate complexes can exchange thiolates without the involvement of Co(ii).

### Comproportionation hypothesis and reactions with Co^I^ compounds

**Scheme 8:**
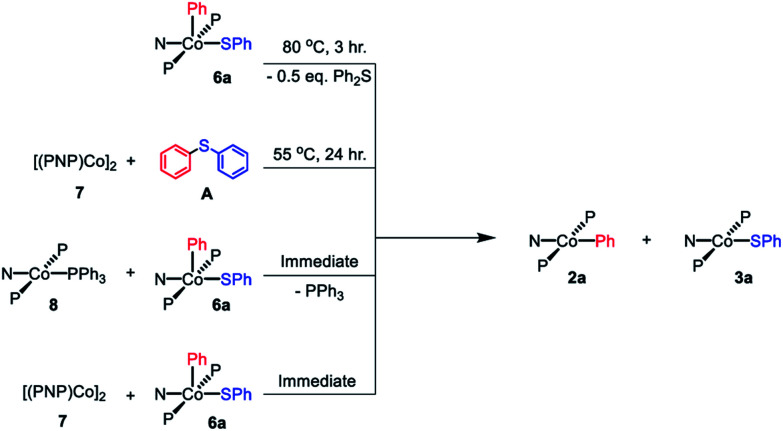
Reaction pathways shown to generate 1 : 1 mixture of **2a** and **3a**.

By analogy with our work on pincer rhodium complexes,^13^,^15^,^19^ we envisioned that after the concerted C–S reductive elimination from **6a**, an unsaturated (PNP)Co fragment (**12**) would be generated. We further hypothesized that this unsaturated (PNP)Co species **12** undergoes rapid comproprotionation with the remaining **6a** to generate the observed Co(ii) products.^60^–^62^ To test this hypothesis, **7** was combined with **6a** in benzene at ambient temperature. An immediate color change to green was observed upon mixing, indicating the formation of (PNP)Co^II^ complexes (Scheme 8). ^1^H NMR spectroscopic observation confirmed the formation of **2a** and **3a** in a 1 : 1 ratio. Similarly, mixing freshly made **8** with **6a** resulted in an immediate comproportionation producing **2a** and **3a** in a 1 : 1 ratio by ^1^H NMR spectroscopy (Scheme 8). In this case, free triphenylphosphine was also observed by ^1^H and ^31^P{^1^H} NMR spectroscopy.

### Reversibility of C–S RE?

Treatment of **7** with diphenyl sulfide (**A**) and heating overnight^63^ in a 55 °C oil bath resulted in the formation of **2a** and **3a** in a 1 : 1 ratio (Scheme 8). This experiment shows that Co(i) here can cleave a C–S bond in a diarylsulfide, suggesting that C–S RE might be reversible. A related observation is that thermolysis of **6b** in the presence of **A** resulted in the formation of a small amount of **C** in addition to **D** (Scheme 7, bottom); which can be interpreted as occasional trapping of a Co(i) species formed in the RE of **D** by Ph_2_S (**A**) as opposed to by the Co(iii) starting material **6b**. By way of a control experiment, thermolysis of **2b** and **3b** with **A** at 80 °C for 7 d resulted in no detectable change, establishing that Co^II^ compounds do not react with a diarylsulfide.

**Scheme 9:**
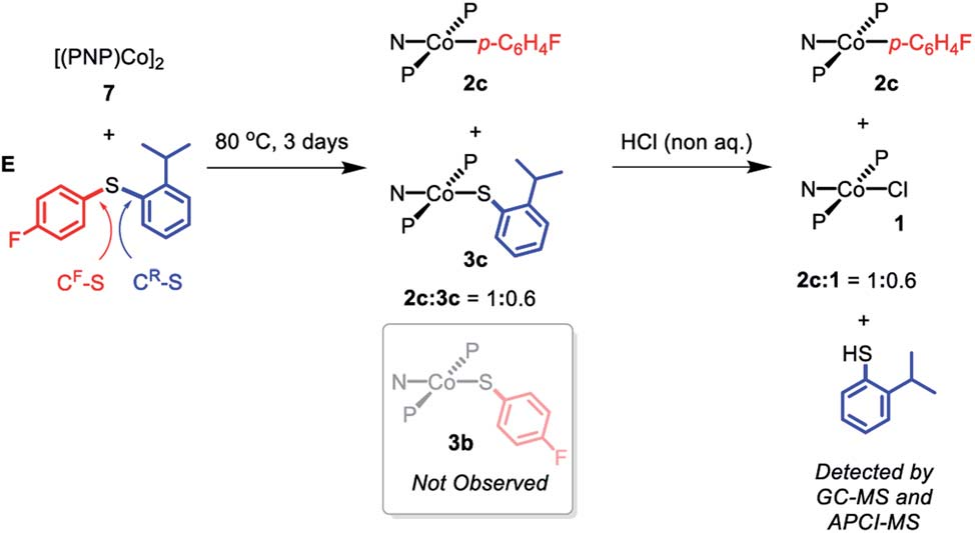
Thermolysis of [(PNP)Co]_2_ (**7**) with 2-isoropylphenyl-4′-fluorophenyl sulfide (**E**) and the following treatment with non-aqueous HCl.

In order to gain some insight into whether this C–S cleavage by Co(i) occurs *via* concerted OA, we subjected **7** to thermolysis with 2-isopropylphenyl-4′-fluorophenyl sulfide (**E**). Based on what we learned of the preferences of the (pincer)Rh systems in OA with aryl halides,^13^,^14^,^19^ it seemed reasonable to assume that the concerted OA mechanism with (PNP)Co should favor the C–S bond unencumbered by the *ortho*-isopropyl substituent (C^F^–S, Scheme 9). DFT calculations (see ESI†) predicted that homolytic cleavage of the C^F^–S bond is 1.6 kcal mol^–1^ less thermodynamically favorable than the cleavage of the C–S bond connecting to the 2-isopropylphenyl substituent (C^R^–S, Scheme 9). Thus, a radical abstraction mechanism for the C–S cleavage might be expected to favor the cleavage of C^R^–S.

Heating the mixture of **7** with **E** in at 80 °C for three days resulted in the complete consumption of **7** with the formation of a 1 : 1 mixture of **2c** and **3c** (Scheme 9). Compound **3b** was not observed by either ^1^H or ^19^F NMR spectroscopy. Treatment of this solution with anhydrous HCl resulted in the formation of **1** along with **2c** with presumed liberation of 2-isopropylthiophenol. GC-MS and APCI-MS analysis of this solution revealed the formation of 2-isopropylthiophenol and **E** as the only two volatile components; *p*-FC_6_H_4_SH was not detected. We did not detect any biaryl or bisulfide products by GC-MS. These observations show that only the C^F^–S bond was cleaved, consistent with a concerted OA C–S activation pathway.^64^

### DFT calculations

**Scheme 10:**
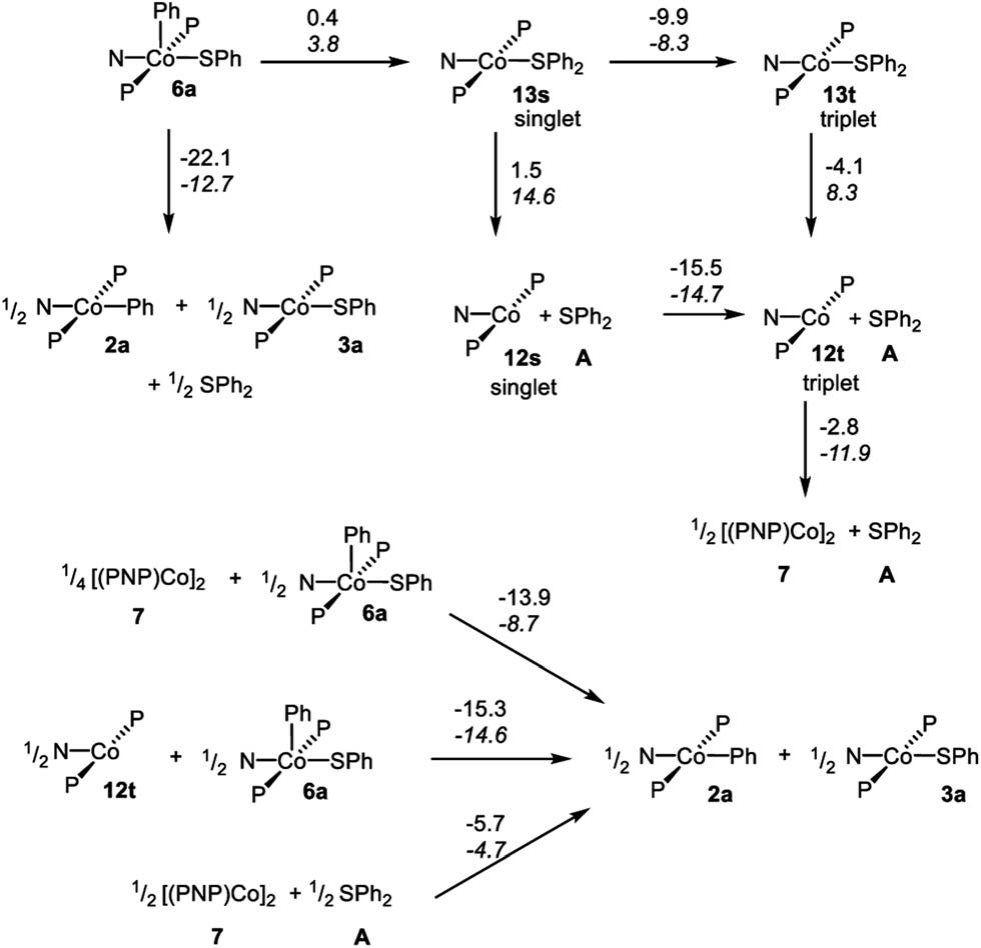
DFT calculated energies for the various transformations. Reaction free energies (at 298 K) are given over the arrows on top; reaction enthalpies in italic below. All the energies are in kcal mol^–1^, normalized per one mole of Co.

**Fig. 4:**
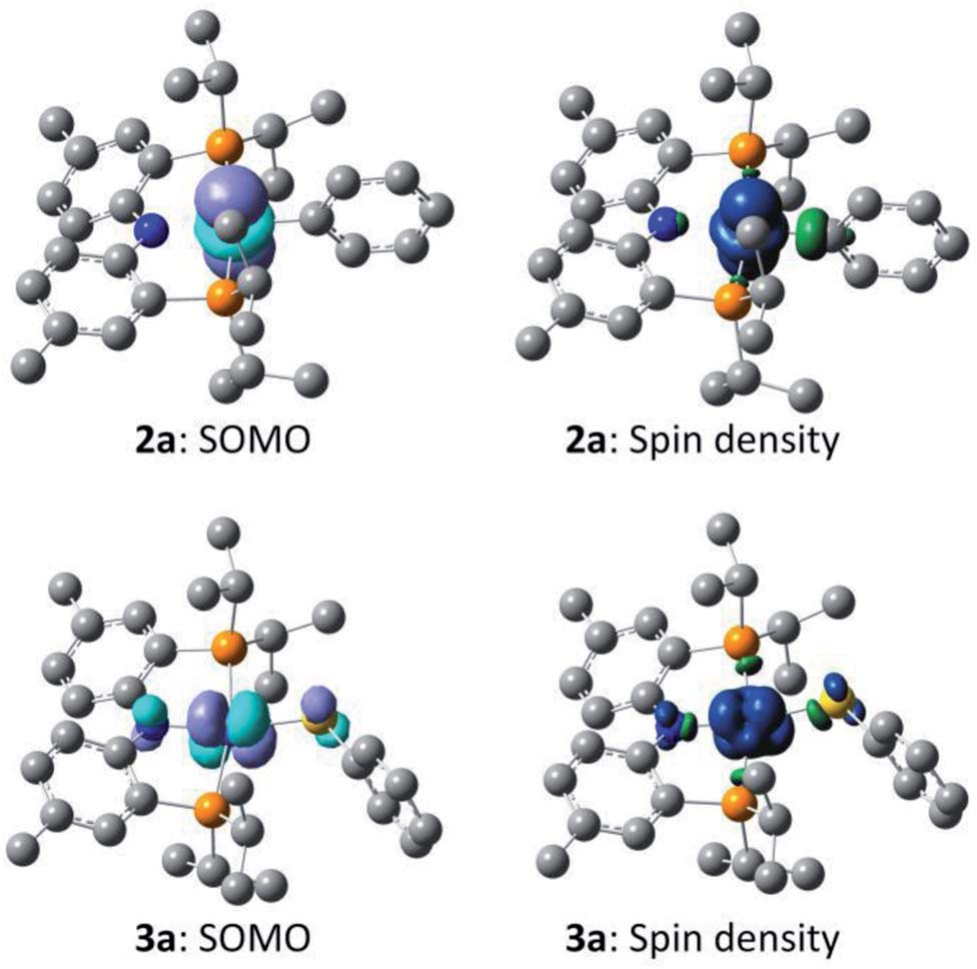
Depictions of the calculated singly occupied molecular orbital (SOMO) for **2a** and **3a** (left, isovalue 0.04), and of the calculated spin-density plots for **2a** and **3a** (right, isovalue 0.004). Hydrogen atoms are omitted for clarity.

In order to gain better understanding of the system, DFT calculations were carried out with the Gaussian 09 (ref. 65) program to address a few salient points (Scheme 10). The geometries were optimized using [B3LYP/LANL2DZ/6-31G(d)] level of theory^66^ in the gas phase and the energies for these geometries were then determined with the [M06/SDD/6-311+G(d,p)] method^67^ incorporating the benzene solvent effect *via* the SMD model.^68^ Further details are given in the ESI.† We first evaluated the thermodynamics of the overall observed reaction. Conversion of 1 equiv. of **6a** into a 0.5 : 0.5 : 0.5 mixture of **2a** : **3a** : **A** was calculated to be favorable by –22.1 kcal mol^–1^ in free energy. The geometries of **2a** and **3a** in the doublet ground state were approximately square-planar geometry and consistent with the X-ray structure of **2b**. The calculated structure of **6a** reproduced the overall geometry determined in the XRD study, as well. Fig. 4 shows the calculated SOMO's and spin density profiles fo **2a** and **3a**. Interestingly, the nature of the SOMO's in these two compounds differs. In **2a**, it is essentially a pure d_*z*^2^_, whereas in **3a** it is primarily d_*xz*_ (*x* axis along Co–S) with small contributions from the amido and thiolate ligands. This disparity reflects the fact that a stronger σ-donor Ph elevates the energy of d_*z*^2^_ in **2a** relative to d_*xz*_. In either case, the SOMO is firmly metal-based.

We then considered the monomeric Co(i) intermediates in the reaction. Both the “naked” (PNP)Co fragment **12** and its SPh_2_ adduct **13** were calculated to favor a triplet ground state (by 15.5 kcal mol^–1^ and 9.9 kcal mol^–1^ in free energy, respectively). The geometry of the triplet (PNP)Co(SPh_2_) (**13t**) about Co is decidedly not planar, and can be described as attempting to approach tetrahedral within the constraint of the pincer. The array of donor atoms in **13t** is the same as in the low-spin doublet **3a**, but all the calculated bond distances to Co are considerably longer, especially that for the C–S bond (2.538 Å in **13t***vs.* 2.264 Å in **3a**).

The reductive C–S coupling in **6a** to give singlet (PNP)Co(SPh_2_) (**13s**) is nearly ergoneutral, but the conversion of **6a** to **13t** is favorable on the enthalpy (by 4.5 kcal mol^–1^) and on the free energy (by 9.5 kcal mol^–1^) surfaces. The dissociation of SPh_2_ from **13t** to give triplet **12t** and free SPh_2_ is endothermic, but is of course favored entropically, resulting in a favorable free energy of dissociation. Thus the complete RE from **6a** to give **12t** and **A** is exoergic by 13.6 kcal mol^–1^, but that is less favorable than the formation of a mixture of **2a** : **3a** : **A**.

The dimerization of **12t** to form **7** was calculated to be enthalpically favorable, but disfavored entropically and overall slightly exoergic (by 2.8 kcal mol^–1^ per Co). This is consistent with the experimental observation of the dimer **7** as the ground state.

The thermodynamics of the standalone Co(i)/Co(iii) comproprotionation reactions were calculated to be consistent with our hypothesis outlined above. The reaction of **12t** with **6a** to give **2a** and **3a** was found to be exothermic and exoergic (by –14.6 and –15.3 kcal mol^–1^, respectively). A similar comproportionation starting from **7** instead of **12t** was also found to be favorable (by –13.9 kcal mol^–1^ per mole of Co).

**Fig. 5:**
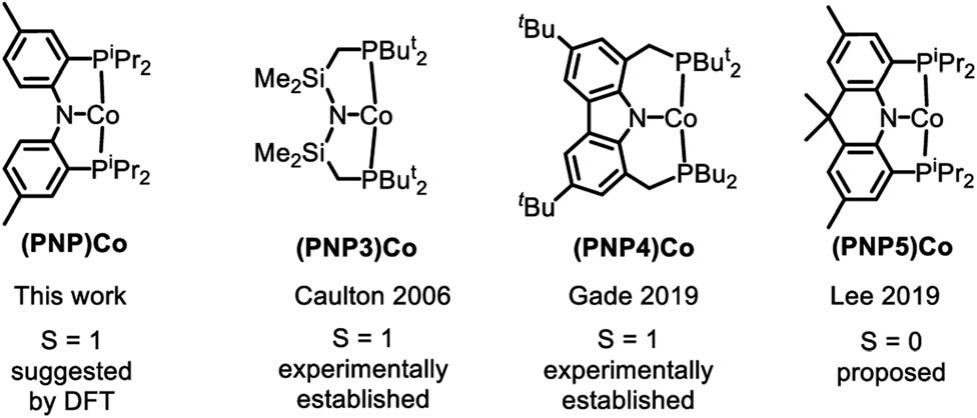
Examples of three-coordinate Co complexes supported by various anionic PNP ligands.

The substantial (*ca.* 15 kcal mol^–1^) calculated preference for the triplet state of (PNP)Co (**12t**) is at odds with the recent report by Lee and coworkers, which presented three-coordinate (PNP5)Co as a singlet species (Fig. 5).^69^ This interpretation by Lee *et al.* is also at odds with the unambiguously established triplet ground states for the (PNP3)Co and (PNP4)Co by the Caulton^70^ and Gade groups,^52^ respectively (Fig. 5). Both (PNP3)Co^70^ and (PNP4)Co^52^ were isolated and fully characterized, including by X-ray crystallography, magnetic moment measurement, as well as by elemental analysis for (PNP4)Co. (PNP5)Co was purported to be isolated, but no structural determination or magnetic moment was reported, and satisfactory elemental analysis was not obtained. (PNP5)Co was analyzed by DFT calculations as a singlet, but the calculations examining the viability of the triplet state were not carried out. Given these facts and the very close similarity of the PNP and PNP4 ligands, it would be very surprising indeed if they led to different spin state preferences in **12***vs.* (PNP5)Co. Although we do not observe free **12**, it is also worth pointing out that even its adduct with PPh_3_ (**8**) does not present as a low-spin complex based on the appearance of its NMR spectra. It is possible that the (PNP5)Co system needs to be reexamined more closely.

**Fig. 6:**
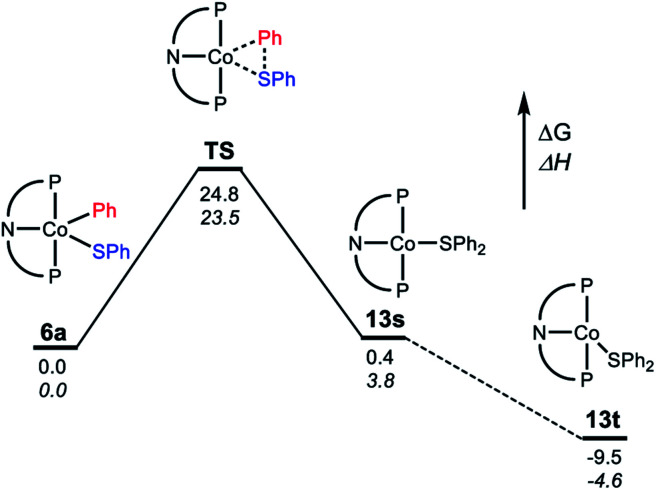
Representation of the reaction coordinate for the RE of SPh_2_ (**A**) from (PNP)Co(Ph)(SPh) (**6a**). Energy values are given below the bars: free energy on top, enthalpy on the bottom in italic.

For the C–S reductive coupling en route to **13** from **6a**, a transition state was found, lying 24.8 kcal mol^–1^ above **6a** in free energy (**TS**, Fig. 6). This transformation requires spin crossover in the process, which we propose happens after the singlet **TS** on the reaction coordinate. A singlet state of the reductive coupling product (PNP)Co(SPh_2_) (**13s**) is only 0.4 kcal mol^–1^ endergonic relative to **6a**. However, **13t** is lower in energy still. The activation barrier magnitude calculated by DFT agrees reasonably well with the experimental observations. The observed half-life of 0.6 h at 80 °C for **6a** corresponds to *ca.* 26 kcal mol^–1^ in free energy barrier 
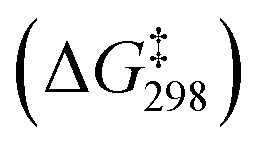
.

**Fig. 7:**
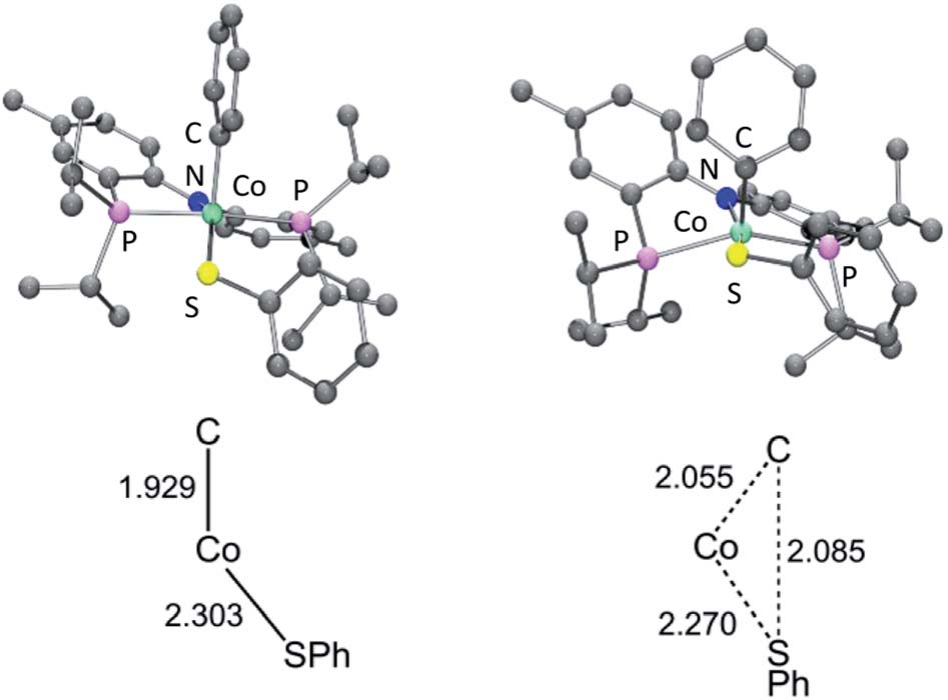
Top: DFT calculated structures of **6a** (left) and **TS** (right). Bottom: Key distances (in Å) in **6a** and **TS**; bottom structures not to scale.

Examination of the geometry of **TS** (Fig. 7) shows that it can be thought of as reflecting the migration of the Co-bound Ph group onto the S atom, which in turn is brought more closely into the plane defined by P/N/P/Co. The Co–S distance in **TS** is actually slightly shorter than in **6a**, and much shorter than calculated in **13t**. The Co–C_Ph_ distance elongates by *ca.* 0.13 Å in **TS** (2.055 Å) *vs.***6a** (1.929 Å), while the newly forming C–S distance (2.085 Å) is about 0.29 Å longer than the expected C–S distances of *ca.* 1.80 Å in Ph_2_S or its complexes. The other geometric feature of **TS** that needs to be emphasized is the necessary rotation of the Co-bound phenyl ring from edge-on relative to S in **6a** to side-on in **TS**. The hindrance of this rotation by the ^i^Pr groups is a major contributor to the magnitude of the activation barrier. This is a rather general observation for the reductive elimination of R–X from five-coordinate d^6^ complexes (pincer)M(R)(X) where R = aryl or alkenyl, first articulated by Goldman and Krogh-Jespersen for the (PCP)Ir system.^71^ We previously discussed this issue for the closely related RE reactions from (pincer)Rh(Ar)(X) complexes.^15^,^16^,^19^

Lastly, we considered the experimental observations of the apparent reversibility of C–S RE in reactions of **A** with Co(i) complexes. Thermodynamically, the experimentally observed reaction of [(PNP)Co]_2_ with Ph_2_S to give **2a** and **3a** was indeed calculated to be favorable (Scheme 10). At first glance, the microscopic reverse C–S OA might appear kinetically feasible as the energy of **13s** is similar to that of **6a**. However, given that (1) **13t**, (2) **12t** + free SPh_2_, and (3) **7** + free SPh_2_ are all considerably lower in energy than **13s**, the barriers for the microscopic reverse C–S OA starting from these states are prohibitively high.

In rationalization, two possibilities might be considered. First, it is possible that our DFT calculations do not accurately describe the relative energies of compounds in different spin states. The second option is that the reaction of **A** with **7** proceeds as an C–S OA within the dimer, without the formation of monomeric intermediates. The putative single C–S OA dicobalt product may then comproportionate intramolecularly to give **2a** and **3a** without the intermediacy of free **6a**. This reaction pathway would thus not be a microscopic reverse of the monomolecular C–S RE. The complexity of the many potential pathways that would need to be considered to properly analyze the reaction of [(PNP)Co]_2_ with **A** has deterred us from pursuing this problem computationally within the scope of this report.

## Conclusion

In summary, (PNP)Co complexes in the +1, +2, and +3 oxidations states relevant to potential cross-coupling reactions were prepared and fully characterized. A switch to a PNP ligand prevented intramolecular reductive elimination of the Co–Ar unit with the central donor of the pincer and permitted observation of concerted C–S reductive elimination. However, it appears that the PNP supporting ligand does not have a strong enough ligand field strength to prevent promotion of an electron from the (PNP)Co fragment to a triplet ground state. This fundamental realization is probably related to the swift Co(i)/Co(iii) comproportionation investigated in this work, which removes potentially catalytically competent odd-oxidation state cobalt complexes from the reaction.

Interestingly, the Co(i)/Co(iii) comproportionation observed here directly mimics the Ni(0)/Ni(ii) comproportionation observed and studied by the Hazari group.^72^ The similarity further underscores the close parallels in reactivity that exist between group 9 metals in the d^8^/d^6^ manifold and the group 10 metals in the d^10^/d^8^ manifold, as well as the contrast between the 3d metals (Co or Ni comproportionate) and the 4d metals (Rh or Pd do not comproprotionate) within the same group.

## Conflicts of interest

There are no conflicts to declare.

## Supplementary Material

Supplementary information

Supplementary information

Supplementary information

Crystal structure data
