# Supplementary material for: Concerted aryl-sulfur reductive elimination from PNP pincer-supported Co(iii) and subsequent Co(i)/Co(iii) comproportionation
Source: Chem Sci. 2020 May 19;11(23):6075–84. doi: 10.1039/d0sc01813a (PMC7480512; doi:10.1039/d0sc01813a)
Supplement: Supplementary file 3 [file SC-011-D0SC01813A-s003.pdf]

Electronic Supplementary Material (ESI) for Chemical Science.
This journal is © The Royal Society of Chemistry 2020

Electronic Supplementary Information

Concerted Aryl-Sulfur Reductive Elimination from PNP Pincer-Supported Co(III) and Subsequent Co(I)/Co(III) Comproportionation

Bryan J. Foley[1], Chandra Mouli Palit[1], Nattamai Bhuvanesh[1], Jia Zhou[2], and Oleg V. Ozerov[1]**

[1]Department of Chemistry, Texas A&M University, 3255 TAMU, College Station, TX 77842.
[2] School of Science, Harbin Institute of Technology, Shenzhen 518055, China

ozerov@chem.tamu.edu and jiazhou@hit.edu.cn

Table of Contents

I. General Considerations & Characterization Methods **S3**

II. X-Ray Structural Determination Details **S5**

III. Synthesis and Characterization **S10**

IV. Mechanistic Study **S18**

V. Reactions of Co (I) Compounds **S33**

VI. NMR Spectra & GC Chromatograms **S40**

VII. Computational Details **S68**

VIII. SI References **S69**

I. General Considerations & Characterization Methods

Unless otherwise specified, all manipulations were performed either inside an argon filled glove box, or by using rigorous Schlenk techniques. Pentane, THF, diethyl ether, and toluene were purified using a PureSolv MD-5 Solvent Purification System and were stored over 4Å molecular sieves in an argon-filled glove box. C_6D_6 was dried over NaK, benzophenone, and 18-crown-6 then stored in an argon-filled glove box over 4Å molecular sieves prior to use. All other chemicals were used as received from commercial vendors. $(PPh_3)_3Co(N(SiMe_3)_2$ (**9**)[1] was prepared via $(PPh_3)_3CoCl$[2] according to the literature procedures. (PNP)H (**10**),[3] 4-tolyllithium[4] were also prepared as described in the literature. Authentic samples of 4-$FC_6H_4SC_6H_5$, 4-$FC_6H_4SC_6H_4$-4'-CH_3, $C_6H_5SC_6H_4$-4-CH_3, and 4-FC_6H_4S-2'-$^i PrC_6H_4$ were prepared using a (POCOP)Rh catalyst previously reported by our group and their spectra matched those previously reported.[5,6]

All NMR spectra were acquired on a Bruker 400 spectrometer (^{1}H NMR, 400.2 MHz) and Varian Inova 500 (^{1}H NMR, 499.703 MHz; ^{13}C NMR, 125.697 MHz; ^{31}P NMR, 202.265 MHz, ^{19}F NMR, 470.135) in denoted solvents. All chemical shifts are reported in δ (ppm). All ^{1}H and ^{13}C NMR spectra were referenced internally to the residual solvent signal (C_6D_6 at δ 7.16 for ^{1}H and δ 128.06 for ^{13}C NMR). ^{19}F NMR spectra were referenced externally to neat trifluoroacetic acid δ -78.55. ^{31}P NMR spectra were externally referenced to an 85% phosphoric acid solution δ 0. Elemental analyses were performed by CALI Labs, Inc. (Highland Park, NJ). Note: All half-widths were acquired with an applied line broadening of 2 Hz; for peaks which overlap, the half-widths were best estimated by taking the frequency difference from the center of the resonance to the half-max of the unobstructed side of the peak and multiplying that value by two.

GC-FID Method

Column parameters: HP-5; 30 meters; I.D. 0.32 mm; Film 0.25 µm

Injection parameters: split-splitless 200-fold split (0.05 min); 1 µL injection; port temp. 250 °C.

Temperature gradient: 80 °C for one min., then ramp 20 °C/min to 210 °C (total time 8.5 min).

Mobile phase: carrier gas, helium; make-up gas, argon. Constant flow; 4.0 mL/min

Detector parameters: temp. 300 °C; FID gas, hydrogen and air.

GC-MS Method

Column parameters: DB-5MS; 30 meters; I.D. 0.25 mm; Film 0.25 µm

Injection parameters: split-splitless (splitless injection); 1 µL injection; port temp. 225 °C

Temperature gradient: 50 °C for 3 min., then ramp 20 °C/min to 300 °C and hold for 3 min. (total time 18.50 min.)

Mobile phase: carrier gas, helium. Constant flow, 1.5 ml/min

Detector parameters: MS Transfer Line 250 °C; mass range 30-500 amu; 70 eV filament

II. X-Ray Structural Determination Details

ORTEP-3 for Windows and POV-Ray were employed for the final data presentation and structure plots.[7,8]

$(^{Me}PNP^{iPr})Co(Tol)$ (2b): (CCDC Deposition #1868267) A Leica MZ 75 microscope was used to identify a suitable brown block with very well-defined faces with dimensions (max, intermediate, and min) 0.272 x 0.183 x 0.074 mm^3 from a representative sample of crystals of the same habit. The crystal mounted on a nylon loop was then placed in a cold nitrogen stream (Oxford) maintained at 110 K.

A BRUKER APEX 2 X-ray (three-circle) diffractometer was employed for crystal screening, unit cell determination, and data collection. The goniometer was controlled using the APEX2 software suite, v2008-6.0.[9] The sample was optically centered with the aid of a video camera such that no translations were observed as the crystal was rotated through all positions. The detector was set at 6.0 cm from the crystal sample (APEX2, 512x512 pixel). The X-ray radiation employed was generated from a Mo sealed X-ray tube (K_{α} = 0.70173Å with a potential of 40 kV and a current of 40 mA).

Sixty data frames were taken at widths of 1.0°. These reflections were used in the auto-indexing procedure to determine the unit cell. A suitable cell was found and refined by nonlinear least squares and Bravais lattice procedures. The unit cell was verified by examination of the *h k l* overlays on several frames of data. No super-cell or erroneous reflections were observed.

After careful examination of the unit cell, an extended data collection procedure (5 sets) was initiated using omega scans.

Data Reduction, Structure Solution, and Refinement

Integrated intensity information for each reflection was obtained by reduction of the data frames with the program APEX2.[9] The integration method employed a three-dimensional profiling algorithm and all data were corrected for Lorentz and polarization factors, as well as for crystal decay effects. Finally, the data was merged and scaled to produce a suitable data set. The absorption correction program SADABS[10] was employed to correct the data for absorption effects.

Systematic reflection conditions and statistical tests of the data suggested the space group $P2_1/c$. A solution was obtained readily using XT/XS in APEX2.[9,11] Hydrogen atoms were placed in idealized positions and were set riding on the respective parent atoms. All non-hydrogen atoms were refined with anisotropic thermal parameters. Absence of additional symmetry and voids were confirmed using PLATON (ADDSYM).[12] The structure was refined (weighted least squares refinement on F^2) to convergence.[11,13]

(^{Me}PNP^{iPr})Co(Ph)(OAc) (4a): (CCDC Deposition #1868268) A dark purple, multi-faceted crystal of suitable size and quality (0.10 x 0.05 x 0.02 mm) was selected using an optical microscope and mounted onto a nylon loop. Low temperature (150 K) X-ray data were obtained on a Bruker APEX2 CCD based diffractometer (Mo sealed X-ray tube, K_α = 0.71073 Å). All diffractometer manipulations, including data collection, integration and scaling were carried out using the Bruker APEX2 software.[9] An absorption correction was applied using SADABS.[10] The structure was initially solved in the monoclinic *C*2/*c* space group using XS[11] (incorporated in SHELXTL). The solution was refined by full-matrix least squares on F^2. No additional symmetry was found using ADDSYM incorporated into the PLATON program.[12] All non-hydrogen atoms were refined with anisotropic thermal parameters. The structure was refined (weighted least squares refinement on F^2) and the final least-squares refinement converged to R_1 = 0.0299 ($I > 2\sigma(I)$, 6839 data) and wR_2 = 0.0805 (F^2, 7986 data, 383 parameters).

(^{Me}PNP^{iPr})Co(Ph)(SPh) (6a): (CCDC Deposition #1868266) A Leica MZ 75 microscope was used to identify a suitable blue block with very well-defined faces with dimensions (max, intermediate, and min) 0.646 x 0.296 x 0.152 mm^3 from a representative sample of crystals of the same habit. The crystal mounted on a nylon loop was then placed in a cold nitrogen stream (Oxford) maintained at 110 K.

A BRUKER APEX 2 X-ray (three-circle) diffractometer was employed for crystal screening, unit cell determination, and data collection. The goniometer was controlled using the APEX2 software suite, v2008-6.0.[9] The sample was optically centered with the aid of a video camera such that no translations were observed as the crystal was rotated through all positions. The detector was set at 6.0 cm from the crystal sample (APEX2, 512x512 pixel). The X-ray radiation employed was generated from a Mo sealed X-ray tube (K_α = 0.70173Å with a potential of 40 kV and a current of 40 mA).

Sixty data frames were taken at widths of 1.0°. These reflections were used in the auto-indexing procedure to determine the unit cell. A suitable cell was found and refined by nonlinear least squares and Bravais lattice procedures. The unit cell was verified by examination of the *h k l* overlays on several frames of data. No super-cell or erroneous reflections were observed.

After careful examination of the unit cell, an extended data collection procedure (6 sets) was initiated using omega scans.

Data Reduction, Structure Solution, and Refinement

Integrated intensity information for each reflection was obtained by reduction of the data frames with the program APEX2.[9] The integration method employed a three-dimensional profiling algorithm and all data were corrected for Lorentz and polarization factors, as well as for crystal

decay effects. Finally, the data was merged and scaled to produce a suitable data set. The absorption correction program SADABS[10] was employed to correct the data for absorption effects.

Systematic reflection conditions and statistical tests of the data suggested the space group $P2_1/n$. A solution was obtained readily using XT/XS in APEX2.[9,11] Hydrogen atoms were placed in idealized positions and were set riding on the respective parent atoms. All non-hydrogen atoms were refined with anisotropic thermal parameters. Absence of additional symmetry and voids were confirmed using PLATON (ADDSYM).[12] The structure was refined (weighted least squares refinement on F^2) to convergence.[11,13]

III. Synthesis and Characterization

NaSPh & NaSC$_6$H$_4$-4-F. To a Schlenk flask was added NaH (~5 mmol), THF (20 mL), and the corresponding thiol (1.5 eq. v. NaH). The reaction was stirred overnight resulting in a colorless solution. Volatiles were removed *in vacuo* and the residue was completely dissolved in 10 mL THF. Pentane (10 mL) was added to the solution resulting in the immediate precipitation of the sodium thiolate. The salt was collected on a glass frit and washed with pentane and diethyl ether prior to drying *in vacuo*. All thiophenolate salts were stored in an Ar-filled glove box and used without further purification in accordance with literature precedent.[14]

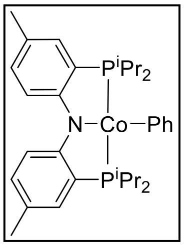

(PNP)Co(Ph) (2a). In an Ar-filled glove box, a 50 mL Schlenk flask was charged with **1** (1.168 g, 2.23 mmol) and 15 mL toluene. After all the solid was dissolved, the flask was placed into a -35 °C freezer for 30 min. Phenyllithium (1.25 mL of a 1.8 M solution in hexanes, 2.25 mmol) was added in one portion. This solution was stirred overnight. The solution was filtered through a pad of Celite and the volatiles were removed *in vacuo*. The residue was dissolved in 2 mL of toluene and layered with 4 mL of pentane. The flask was then placed in a -35 °C freezer overnight affording a green solid. The solid was washed with cold isooctane and then dried under vacuum at room temperature. Yield: 689 mg (55%). ^{1}H NMR (C$_6$D$_6$, 500 MHz): δ 39.6-36.7 (overlapping, $\Delta\nu_{1/2}$ = 180 Hz, $\Delta\nu_{1/2}$ = 960 Hz, 6H), 23.39 ($\Delta\nu_{1/2}$ = 42 Hz, 6H), 14.90 ($\Delta\nu_{1/2}$ = 310 Hz, 12H), 7.49 ($\Delta\nu_{1/2}$ = 21 Hz, 2H), 0.09 ($\Delta\nu_{1/2}$ = 1000 Hz, 12H), -13.99 ($\Delta\nu_{1/2}$ = 70 Hz, 2H), -28.91 ($\Delta\nu_{1/2}$ = 130 Hz, 2H), -92.51 ($\Delta\nu_{1/2}$ = 990 Hz, 2H).

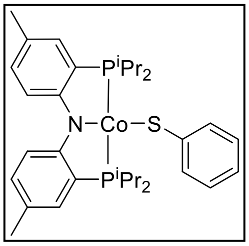

(PNP)Co(SPh) (3a). In an Ar-filled glove box, a 20 mL scintillation vial was charged with **1** (152 mg, 0.291 mmol) and 10 mL THF. To this solution, NaSPh (63 mg, 0.48 mmol) was added. The solution changed from deep blue to dark green over 30 min. The reaction was left to stir overnight. The volatiles were removed *in vacuo* and the product was extracted with 10 mL of pentane and filtered through a plug of Celite. The volatiles were removed again, yielding a green solid. Yield: 43 mg (74%). ^{1}H NMR (C_6D_6, 500 MHz): δ 25.21 ($\Delta\nu_{1/2}$ = 190 Hz, 6H), 23.84 ($\Delta\nu_{1/2}$ = 280 Hz, 2H), 10.89 ($\Delta\nu_{1/2}$ = 49 Hz, 2H), 9.42 ($\Delta\nu_{1/2}$ = 1400 Hz, 2H), 6.82 ($\Delta\nu_{1/2}$ = 52 Hz, 3H), 5.7-2.2 (overlapping, $\Delta\nu_{1/2}$ = 400 Hz, $\Delta\nu_{1/2}$ = 700 Hz, 25 H), -10.49 ($\Delta\nu_{1/2}$ = 520 Hz, 2H), -20.49 ($\Delta\nu_{1/2}$ = 380 Hz, 2H).

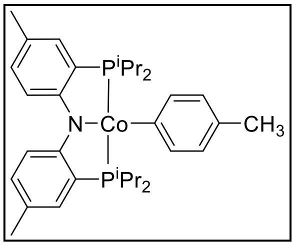

(PNP)Co(Tol) (2b). In an Ar-filled glove box, a 50 mL Schlenk flask was charged with **1** (1.366 g, 2.61 mmol) and 15 mL THF. Freshly prepared 4-tolyllithium (0.256 g, 2.64 mmol) was weighed into a scintillation vial and dissolved in 15 mL THF. Both of these solutions were placed into a -35 °C freezer within the glove box. After one hour, the 4-tolyllithium solution was pipetted into the Schlenk flask and the mixture was stirred overnight. The volatiles were removed *in vacuo* and the residue was dissolved in 15 mL toluene. After Celite filtration, the volatiles were removed *in vacuo* and the resulting solid was dissolved in 5 mL of pentane. This pentane solution was placed into a -35 °C freezer overnight affording green precipitate. Yield: 1.023 g (68%) The supernatant was concentrated and placed back into the freezer yielding an additional 0.254 g (85% overall). ^{1}H NMR (C_6D_6, 500 MHz): δ 39.6-36.2 (overlapping, $\Delta\nu_{1/2}$ = 180 Hz, $\Delta\nu_{1/2}$ = 860 Hz, 6H), 23.56 ($\Delta\nu_{1/2}$ = 34 Hz, 6H), 14.80 ($\Delta\nu_{1/2}$ = 320 Hz, 12H), 7.53 ($\Delta\nu_{1/2}$ = 17 Hz, 2H), 0.15 ($\Delta\nu_{1/2}$ = 870 Hz, 12H), -8.09 ($\Delta\nu_{1/2}$ = 14 Hz, 3H, tolyl CH_3), -12.65 ($\Delta\nu_{1/2}$ = 46

Hz, 2H), -29.04 (Δ$\nu_{1/2}$ = 110 Hz, 2H), -93.76 (Δ$\nu_{1/2}$ = 1000 Hz, 2H). Elem. Anal. Calcd. for $C_{33}H_{47}CoNP_2$: C, 68.50; H, 8.19. Found: C, 68.17; H, 7.85.

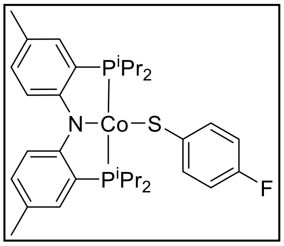

(PNP)Co(S-4-C_6H_4F) (3b). In an Ar-filled glove box, a 25 mL Schlenk flask was charged with **1** (0.209 g, 0.398 mmol) and 10 mL of THF. Sodium 4-fluorothiophenolate (0.083 g, 0.55 mmol) was added and the solution was stirred overnight. The volatiles were removed *in vacuo* and the residue was extracted with 10 mL pentane. The solution was passed through a plug of Celite and then dried under vacuum. Yield: 0.210 g (85%). ^{1}H NMR (C_6D_6, 500 MHz): δ 25.51 (Δ$\nu_{1/2}$ = 200 Hz, 6H), 23.69 (Δ$\nu_{1/2}$ = 260 Hz, 2H), 11.04 (Δ$\nu_{1/2}$ = 50 Hz, 2H), 8.60 (Δ$\nu_{1/2}$ = 1100 Hz, 2H), 6.48 (Δ$\nu_{1/2}$ = 52 Hz, 2H), 5.8-2.3 (overlapping, Δ$\nu_{1/2}$ = 400 Hz, Δ$\nu_{1/2}$ = 600 Hz, 24H), -9.24 (Δ$\nu_{1/2}$ = 460 Hz, 2H), -20.73 (Δ$\nu_{1/2}$ = 380 Hz, 2H). ^{19}F NMR (C_6D_6, 470 MHz): δ -117.4.

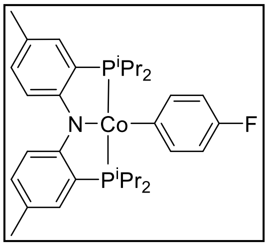

Synthesis of **(PNP)Co(*p*-C_6H_4F) (2c)**. In an Ar-filled glove box, a 50 mL Schlenk flask was charged with **1** (0.262 g, 0.50 mmol) and 20 mL of THF. This solution was placed into a -35 °C freezer for 40 minutes. A 1 M solution of 4-fluorophenylmagnesium bromide (1.0 mL, 1.0 mmol) was added rapidly dropwise and the reaction was stirred overnight. The volatiles were removed *in vacuo* and the hard residue was extracted with 20 mL pentane overnight while stirring. The solution was filtered through a pad of Celite and the volatiles were removed yielding a dark green solid. Yield: 227 mg (78%) ^{1}H NMR (C_6D_6, 500 MHz): δ 39.06 & 37.80 (overlapping, Δ$\nu_{1/2}$ = 170 Hz, Δ$\nu_{1/2}$ = 1100 Hz, 6H), 24.20 (Δ$\nu_{1/2}$ = 41 Hz, 6H), 14.91 (Δ$\nu_{1/2}$ = 350 Hz, 12H), 7.75 (Δ$\nu_{1/2}$ = 22 Hz, 2H), 0.19 (Δ$\nu_{1/2}$ = 1100 Hz, 12H), -11.56 (Δ$\nu_{1/2}$ = 51 Hz, 2H), -29.83 (Δ$\nu_{1/2}$ = 120 Hz, 2H), -93.68 (Δ$\nu_{1/2}$ = 1200 Hz, 2H). ^{19}F NMR (C_6D_6, 470 MHz): δ -160.0.

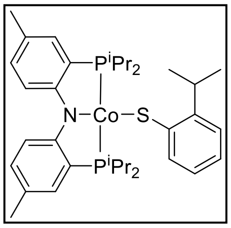

(PNP)Co(S-2-^{i}PrC$_6$H$_4$) (3c). In an Ar-filled glove box, a 20 mL scintillation vial was charged with **1** (0.148 g, 0.282 mmol) and 10 mL THF. In a separate vial, sodium 2-isopropylthiolate (60 mg, 0.34 mmol) was dissolved in 5 mL THF. The thiolate solution was pipetted into the vial containing the cobalt complex and allowed to stir overnight. The volatiles were removed *in vacuo* and the residue was dissolved in pentane. After Celite filtration, the volatiles were removed again yielding a green solid. Yield: 112 mg (63%). ^{1}H NMR (C_6D_6, 400 MHz): δ 24.53 ($\Delta\nu_{1/2}$ = 250 Hz, overlap, 4H), 23.83 ($\Delta\nu_{1/2}$ = 380 Hz, overlap, 2H), 10.71 ($\Delta\nu_{1/2}$ = 50 Hz, overlap, 2H), 10.52 ($\Delta\nu_{1/2}$ = 80 Hz, overlap, 1H), 6.4-4.0 (overlapping, $\Delta\nu_{1/2}$ = 90 Hz, $\Delta\nu_{1/2}$ = 600 Hz, 12H), 3.8-2.1 (overlapping $\Delta\nu_{1/2}$ = 80 Hz, $\Delta\nu_{1/2}$ = 850 Hz, 12H), -1.66 ($\Delta\nu_{1/2}$ = 70 Hz, 5H), -15.91 ($\Delta\nu_{1/2}$ = 940 Hz, 2H), -19.90 ($\Delta\nu_{1/2}$ = 430 Hz, 2H).

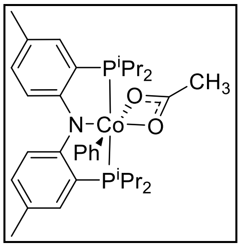

(PNP)Co(Ph)(OAc) (4a). In an Ar-filed glove box, a 20 mL scintillation was charged with **2a** (0.550 g, 0.974 mmol) and 10 mL toluene. Bis(acetoxyiodo)benzene (0.183 g, 0.57 mmol) was added in one portion and the solution was stirred overnight. The volatiles were removed *in vacuo*. The residue was dissolved in toluene and filtered through a pad of Celite. The volatiles were removed again, and the resulting solid was dissolved in pentane and placed into a -35 °C freezer to afford a tan precipitate. Yield: 0.269 g (44%). ^{1}H NMR (C_6D_6, 500 MHz): δ 7.46 (dt, *J* = 8.4 Hz, *J* = 2.0 Hz, 2H), 7.06 (dt, *J* = 7.8 Hz, *J* = 1.4 Hz, 1H), 7.01 (dd, *J* = 5.2 Hz, *J* = 3.2 Hz, 2H), 6.93 (td, *J* = 7.5 Hz, *J* = 1.9 Hz, 1H), 6.89 (tt, *J* = 7.0 Hz, *J* = 1.0 Hz, 1H), 6.78 (ddd, *J* = 7.9 Hz, *J* = 7.0 Hz, *J* = 1.9 Hz, 1H), 6.67 (dd, *J* = 8.5 Hz, *J* = 1.8 Hz, 2H), 6.62 (ddd, *J* = 7.9 Hz, *J* = 1.8 Hz, *J* = 0.8 Hz, 1H), 2.48 (m, 2H), 2.15 (s, 8H, overlapping tolyl methyl and methine resonances), 1.69 (s, 3H, OAc–CH_3), 1.37 (dvt, *J* = 6.3 Hz, *J* = 7.3 Hz, 6H, P–CH–(CH_3)$_2$), 1.28 (dvt, *J* = 6.4 Hz, *J* = 6.3

Hz, 6H, P–CH–(CH_3)$_2$), 1.04 (dvt, *J* = 7.2 Hz, *J* = 7.2 Hz, 6H, P–CH–(CH_3)$_2$), 0.59 (dvt, *J* = 6.8 Hz, *J* = 6.8 Hz, 6H, P–CH–(CH_3)$_2$). $^{13}C\{^1H\}$ NMR (C_6D_6, 125 MHz): δ 183.2 (t, *J* = 1.8 Hz, C=O), 159.3 (vt, *J* = 10.8 Hz), 140.4 (br s), 135.6 (t, *J* = 2.3 Hz), 131.84 (s), 131.41 (overlapping signals), 125.96 (vt, *J* = 2.5 Hz), 124.70 (t, *J* = 3.0 Hz), 124.60 (t, *J* = 2.3 Hz), 123.28 (br s), 122.88 (m, *J* = 19.8 Hz, *J* = 19.8 Hz), 119.84 (vt, *J* = 4.5 Hz), 23.81 (vt, J= 10.7 Hz, P–*C*H–(CH_3)$_2$), 23.04 (br s, OAc–*C*H_3), 22.6 (vt, *J* = 7.6 Hz, P–*C*H–(CH_3)$_2$), 20.72 (s, Ar-*C*H_3), 19.2 (vt, *J* = 2.1 Hz, P–CH–(*C*H_3)$_2$), 18.6 (br s, P–CH–(*C*H_3)$_2$), 17.88 (br s, two overlapping P–CH–(*C*H_3)$_2$). $^{31}P\{^1H\}$ NMR (C_6D_6, 202 MHz): δ 40.1. Elem. Anal. Calcd for $C_{34}H_{48}CoNO_2P_2$: C, 65.48; H, 7.76. Found: C, 65.38; H, 7.56.

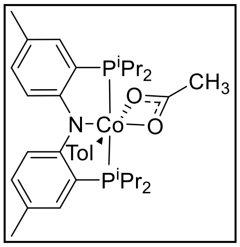

(PNP)Co(Tol)(OAc) (4b). In an Ar-filed glove box, a 50 mL scintillation was charged with **2b** (0.219 g, 0.378 mmol) and 30 mL toluene. Bis(acetoxyiodo)benzene (0.063 g, 0.20 mmol) was added in one portion and the solution was stirred overnight. The volatiles were removed *in vacuo*. The residue was dissolved in pentane and filtered through a plug of Celite. The volatiles were removed again, and the resulting solid was dissolved in 1 mL of pentane and placed into a -35 °C freezer to afford a brown-tan precipitate. Yield: 0.160 g (65%). ^1H NMR (C_6D_6, 500 MHz): δ 7.46 (dt, *J* = 8.5 Hz, *J* = 2.1 Hz, 2H), 7.02 (dd, *J* = 5.4 Hz, *J* = 3.2 Hz, 2H), 6.94 (dd, *J* = 8.1 Hz, *J* = 1.6 Hz, 1H), 6.83 (dd, *J* = 8.2 Hz, *J* = 1.9 Hz, 1H), 6.67 (dd, *J* = 8.5 Hz, *J* = 1.9 Hz, 2H), 6.61 (dd, *J* = 8.1 Hz, *J* = 1.8 Hz, 1H), 6.47 (dq, *J* = 8.0 Hz, *J* = 2.0 Hz , 1H), 2.49 (m, 2H, P–C*H*–(CH_3)$_2$), 2.21 (s, 3H, tolyl–CH_3), 2.16 (s, 8H, overlapping backbone tolyl methyl and methine resonances), 1.70 (s, 3H, OAc–CH_3), 1.38 (dvt, *J* = 7.3 Hz, *J* = 7.3 Hz, 6H, P–CH–(CH_3)$_2$). $^{13}C\{^1H\}$ NMR (C_6D_6, 125 MHz): δ 183.18 (t, *J* = 1.7 Hz, C=O), 159.42 (vt, *J* = 10.9 Hz), 140.10 (t, *J* =1.9 Hz), 135.26 (t, *J* = 2.3 Hz), 131.92 (t, *J* = 2.0 Hz), 131.84 (s), 131.36 (s), 127.01 (t, *J* = 2.7 Hz), 125.73 (vt, *J* = 2.5

Hz), 124.62 (t, J = 3.0 Hz), 122.99 (m, J = 19.6 Hz, J = 19.8 Hz), 119.81 (vt, J = 4.6 Hz), 23.78 (vt, J = 10.5 Hz, P–CH–(CH_3)$_2$), 23.04 (s, OAc–CH_3), 22.65 (vt, J = 7.5 Hz, P–CH–(CH_3)$_2$), 20.72 (s, backbone tolyl methyls), 20.69 (s, Co–C_6H_4–CH$_3$), 19.17 (vt, J = 2.2 Hz, P–CH–(CH$_3$)$_2$), 18.64 (s, P–CH–(CH$_3$)$_2$), 17.96 (s, P–CH–(CH$_3$)$_2$), 17.93 (s, P–CH–(CH$_3$)$_2$). ^{31}P{^{1}H} NMR (C_6D_6, 202 MHz): δ 40.1 (br s). Elem. Anal. Calcd. for $C_{35}H_{50}CoNO_2P_2$: C, 65.93; H, 7.90. Found: C, 66.22; H, 7.57.

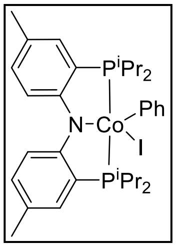

(PNP)Co(Ph)(I) (5a). **Method A:** In an Ar-filled glove box, **4a** (0.100 g, 0.161 mmol) was dissolved in ca. 10 mL of 1:1 mixture of toluene/C_6H_6. To this solution Me_3SiI (23 μL, 0.16 mmol) was added and the reaction was left to stir for 1 h. The volatiles were then removed and the product was extracted with ca. 30 mL of pentane and filtered through a pad of celite on a glass frit. The volatiles were removed and a blue-green solid was obtained (101 mg, 90%). **Method B:** In an Ar-filled glove box, a 25 mL Schlenk flask was charged with **2a** (0.218 g, 0.386 mmol) and 20 mL of toluene. To this flask, a solution of freshly sublimed I_2 (1.0 mL of a 0.19 M solution in THF, 0.19 mmol) was added in one portion. After four hours, the volatiles were removed *in vacuo*. The residue was dissolved in 4 mL pentane and placed into a -35 °C freezer inside the glovebox. The supernatant was decanted, and the solids were dried *in vacuo*. Yield: 0.152 g (57%). ^{1}H NMR (C_6D_6, 500 MHz): δ 7.86 (d, J = 8.6 Hz, 2H), 7.17 (overlapped, 3H), 6.72 (dd, J = 8.6 Hz, J = 2.0 Hz, 2H), 6.52 (br t, J =7.1 Hz, 1H), 6.38 (ddd, J = 8.9 Hz, J = 7.1 Hz, J = 2.0 Hz, 1H), 6.14 (ddd, J = 9.0 Hz, J = 7.2 Hz, J = 2.0 Hz, 1H), 5.92 (d, J = 8.2 Hz, 1H), 4.06 (observed hept., J = 6.8 Hz, 2H, P–CH–(CH_3)$_2$), 2.67 (m, 2H, P–CH–(CH_3)$_2$), 2.11 (s, 6H, backbone tolyl methyls), 1.67 (br d, J = 6.1 Hz, 6H, P–CH–(CH_3)$_2$), 1.24 (br d, J = 6.5 Hz, 6H, P–CH–(CH_3)$_2$), 0.73 (br d, J = 5.5 Hz, 6H, P–CH–(CH_3)$_2$), 0.37 (br d, J = 6.2 Hz, 6H, P–CH–(CH_3)$_2$). ^{13}C{^{1}H} NMR (C_6D_6, 125 MHz): δ 162.61 (br s), 152.19 (s), 136.19 (s),

132.88 (s), 131.43 (s), 126.04 (s), 125.44 (s), 123.70 (s), 122.99 (br s), 122.67 (s), 27.02 (br s, P–*C*H–(CH_3)$_2$), 24.83 (br s, P–*C*H–(CH_3)$_2$), 20.61 (s, backbone tolyl methyls), 18.66 (s, P–CH–(*C*H_3)$_2$), 18.62 (s, P–CH–(*C*H_3)$_2$), 17.56 (s, P–CH–(*C*H_3)$_2$), 17.39 (s, P–CH–(*C*H_3)$_2$). ^{31}P{^{1}H} NMR (C_6D_6, 202 MHz): δ 37.7 (br s).

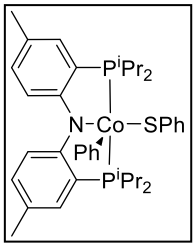

(PNP)Co(Ph)(SPh) (6a). In an Ar-filled glove box, a 20 mL scintillation vial was charged with **4a** (0.269 g, 0.431 mmol) and 10 mL of toluene. To this solution, Me_3SiI (23 μL, 0.16 mmol) was added and the reaction was left to stir overnight. The volatiles were removed *in vacuo*. The residue was dissolved in THF and sodium thiophenolate (0.097 g, 0.73 mmol) was added to the solution. After three hours, the volatiles were then removed and the product was extracted with 20 mL of pentane and filtered through a pad of Celite. The volatiles were removed and a dark blue-green solid was obtained. Yield: 187 mg (64%). ^{1}H NMR (C_6D_6, 500 MHz): δ 7.88 (d, *J* = 8.6 Hz, 2H), 7.80 (overlapping, 3H), 7.10 (br s, 2H), 6.99 (m, 3H), 6.76 (ddd, *J* = 8.6 Hz, *J* = 2.1 Hz, *J* = 0.6 Hz, 1H), 6.62 (br t, *J* = 7.0 Hz, 1H), 6.57 (br s, 1H), 6.28 (br s, 1H), 6.01 (br s, 1H), 2.89 (observed hept. *J* = 6.9 Hz, 2H, P–C*H*–(CH_3)$_2$), 2.49 (m, 2H, P–C*H*–(CH_3)$_2$), 2.16 (s, 6H, backbone tolyl methyls), 1.26 (br d, *J* = 5.9 Hz, 6H, P–CH–(*C*H_3)$_2$), 1.12 (br d, *J* = 5.7 Hz, 6H, P–CH–(*C*H_3)$_2$), 0.95 (br d, *J* = 6.1 Hz, 6H, P–CH–(*C*H_3)$_2$), 0.44 (br d, *J* = 5.9 Hz, 6H, P–CH–(*C*H_3)$_2$). ^{13}C{^{1}H} NMR (C_6D_6, 125 MHz): δ 162.01 (br), 149.07 (br), 146.65 (br), 139.90 (br), 135.93 (two overlapping signals), 132.29 (two overlapping signals), 131.31 (two overlapping signals) 126.73, 125.76 (br), 124.83 (br), 124.74, 123.52, 121.48, 25.84 (br, P–*C*H–(CH_3)$_2$), 24.80 (br, P–*C*H–(CH_3)$_2$), 20.56 (s, backbone tolyl methyls), 20.00 (s, P–CH–(*C*H_3)$_2$), 18.07 (s, P–CH–(*C*H_3)$_2$), 17.71 (s, P–CH–(*C*H_3)$_2$), 17.29 (s, P–CH–(*C*H_3)$_2$). ^{31}P{^{1}H} NMR (C_6D_6, 202 MHz): δ 31.1 (br s). Elem. Anal. Calcd for $C_{38}H_{50}CoNP_2S$: C, 67.74; H, 7.48. Found: C, 67.85; H, 7.18.

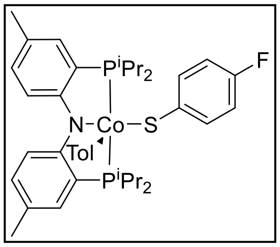

(PNP)Co(Tol)(SAr^F) (6b). In an Ar-filled glove box, a 20 mL scintillation vial was charged with **4b** (65 mg, 0.10 mmol) and 10 mL toluene. To this solution was added trimethylsilyliodide (20 µL, 0.14 mmol) in one portion and the solution was stirred overnight. The volatiles were removed *in vacuo*. The residue was dissolved in THF and sodium 4-fluorothiophenolate (0.033 g, 0.22 mmol) was added. After 30 minutes, the volatiles were removed and the residue was dissolved in 10 mL pentane and filtered through a pad of Celite. The filtrate was dried *in vacuo* resulting in a dark blue solid. Yield: 0.063 g (88%). ^{1}H NMR (C_6D_6, 500 MHz): δ 7.87 (d, *J* = 8.6 Hz, 2H), 7.60 (overlapping, dd, *J* = 8.7 Hz, *J* = 5.5 Hz, and broad singlet, 3H), 7.10 (s, 2H), 6.77 (dd, *J* = 8.6 Hz, *J* = 1.6 Hz 2H), 6.69 (t, *J* = 8.7 Hz, 2H) 6.45 (br s, 1H), 6.12 (br s, 1H), 5.86 (br s, 1H), 2.84 (observed hept. *J* = 6.9 Hz, 2H, P–C*H*–(CH_3)$_2$), 2.46 (m, 2H, P–C*H*–(CH_3)$_2$), 2.18 (s, 6H, backbone tolyl methyls), 2.02 (s, 3H, tolyl–CH_3) 1.23 (br d, *J* = 4.4 Hz, 6H, P–CH–($\mathit{C}H_3$)$_2$), 1.12 (br d, *J* = 5.8 Hz, 6H, P–CH–($\mathit{C}H_3$)$_2$), 0.92 (br d, *J* = 5.4 Hz, 6H, P–CH–($\mathit{C}H_3$)$_2$), 0.44 (br d, *J* = 5.3 Hz, 6H, P–CH–($\mathit{C}H_3$)$_2$). ^{13}C{^{1}H} NMR (C_6D_6, 125 MHz): δ 162.02 (br, C–N), 161.7 (d, *J* = 243 Hz, *C*–F), 148.30 (br), 141.3 (br), 139.57 (br), 137.20 (d, *J* = 7.1 Hz, C_6H_4F), 132.47, 132.27, 131.30, 126.78, 125.85 (br), 121.53, 114.91 (d, *J* = 21 Hz, C_6H_4F), 25.79 (br, P–*C*H–(CH_3)$_2$), 24.81 (br, P–*C*H–(CH_3)$_2$), 20.57 (s, backbone tolyl methyls), 20.28 (s, Co–C_6H_4–$\mathit{C}H_3$), 19.95 (s, P–CH–($\mathit{C}H_3$)$_2$), 18.10 (s, P–CH–($\mathit{C}H_3$)$_2$), 17.67 (s, P–CH–($\mathit{C}H_3$)$_2$), 17.27 (s, P–CH–($\mathit{C}H_3$)$_2$). ^{31}P{^{1}H} NMR (C_6D_6, 202 MHz): δ 30.2 (br s). ^{19}F NMR (C_6D_6, 470 MHz): δ -119.4.

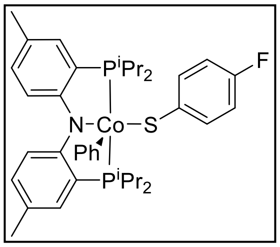

Spectroscopic observation of (PNP)Co(Ph)(SAr^F) (6c). In an Ar-filled glove box, a 10 mL Schlenk flask was charged with **4a** (10 mg, 0.016 mmol) and 5 mL of toluene. To this solution, Me_3SiI (10 µL, 0.07 mmol) was added and the reaction was left to stir overnight. The volatiles were

removed *in vacuo*. The residue was dissolved in THF and sodium 4-fluorothiophenolate (0.030 g, 0.019 mmol) was added. After 30 minutes, the volatiles were removed. The residue was dissolved in C_6D_6 for spectroscopic analysis. ^{1}H NMR (C_6D_6, 500 MHz): δ 7.87 (d, *J* = 8.6 Hz, 2H), 7.71 (br s, 1H, Co–Ph), 7.58 (dd, *J* = 8.8 Hs, *J* = 5.5 Hz, 2H), 7.09 (br s, 2H), 6.76 (dd, *J* = 8.6 Hz, *J* = 1.6 Hz, 2H), 6.67 (t, *J* = 8.8 Hz, 2H), 6.62 (br t, *J* = 7.0 Hz, 1H, Co–Ph), 6.57 (br s, 1H, Co–Ph), 6.27 (br s, 1H, Co–Ph), 5.99 (br s, 1H, Co–Ph), 2.84 (observed hept. *J* = 6.9 Hz, 2H, P–C*H*–(CH_3)$_2$), 2.45 (m, 2H, P–C*H*–(CH_3)$_2$), 2.17 (s, 6H, backbone tolyl methyls), 1.22 (br d, *J* = 5.7 Hz, 6H, P–CH–(*C*H_3)$_2$), 1.12 (br d, *J* = 5.5 Hz, 6H, P–CH–(*C*H_3)$_2$), 0.91 (br d, *J* = 5.6 Hz, 6H, P–CH–(*C*H_3)$_2$), 0.41 (br d, *J* = 5.5 Hz, 6H, P–CH–(*C*H_3)$_2$). ^{19}F NMR (C_6D_6, 470 MHz): δ -119.3.

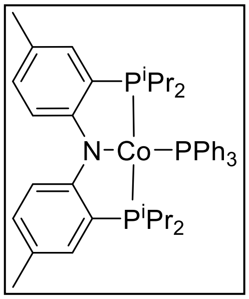

(PNP)Co(PPh$_3$) (8). In an Ar-filled glove box, a J. Young Tube was charged with **9** (0.017 g, 0.022 mmol) and 600 μL C_6D_6. To this solution was added **10** (0.011 g, 0.026 mmol). Immediate ^{1}H NMR observation revealed the appearance of new paramagnetically shifted resonances. This compound could not be isolated because over time, the complexes freely lose triphenylphosphine and make [(PNP)Co]$_2$, even at -35 °C. ^{1}H NMR (C_6D_6, 500 MHz): δ 76.65 ($\Delta\nu_{1/2}$ = 1600 Hz, 2H), 22.55 ($\Delta\nu_{1/2}$ = 120 Hz, 6H), 21.00 ($\Delta\nu_{1/2}$ = 130 Hz, 2H), 15.53 ($\Delta\nu_{1/2}$ = 120 Hz, 2H), 9.34 ($\Delta\nu_{1/2}$ = 160 Hz, 6H), 3.99 ($\Delta\nu_{1/2}$ = 220 Hz, 12H), 3.30 ($\Delta\nu_{1/2}$ = 200 Hz, 16H), -8.65 ($\Delta\nu_{1/2}$ = 360 Hz, 5H) -13.43 ($\Delta\nu_{1/2}$ = 140 Hz, 2H).

IV. Mechanistic Study

Thermolysis of 6a. 6a (4 mg, 0.006 mmol) was dissolved in 600 μL of C_6D_6 in a J. Young tube. To this solution, 1,4-dioxane (2 μL, 0.023 mmol) was added using a syringe to serve as an internal standard. A ^{1}H NMR spectrum of the mixture was acquired and then the NMR tube was placed in an 80 °C oil bath for three hours. The final mixture contained: **2a** (46% of initial **6a**), **3a** (46% of initial **6a**) and **A** (48% of initial **6a**) as observed by ^{1}H NMR spectroscopy.

***In situ* monitoring of consumption of 6a. 6a** (15 mg, 0.022 mmol) was dissolved in 500 µL of C_6D_6 in a J. Young tube. To this solution, 1.4-dioxane (5 µL, 0.058 mmol) was added using a syringe to serve as an internal standard. The tube was heated to 80 °C inside the NMR and monitored continually by ^{1}H NMR spectroscopy. The consumption of **6a** versus the internal standard as monitored by ^{1}H NMR spectroscopy fits a first order correlation.

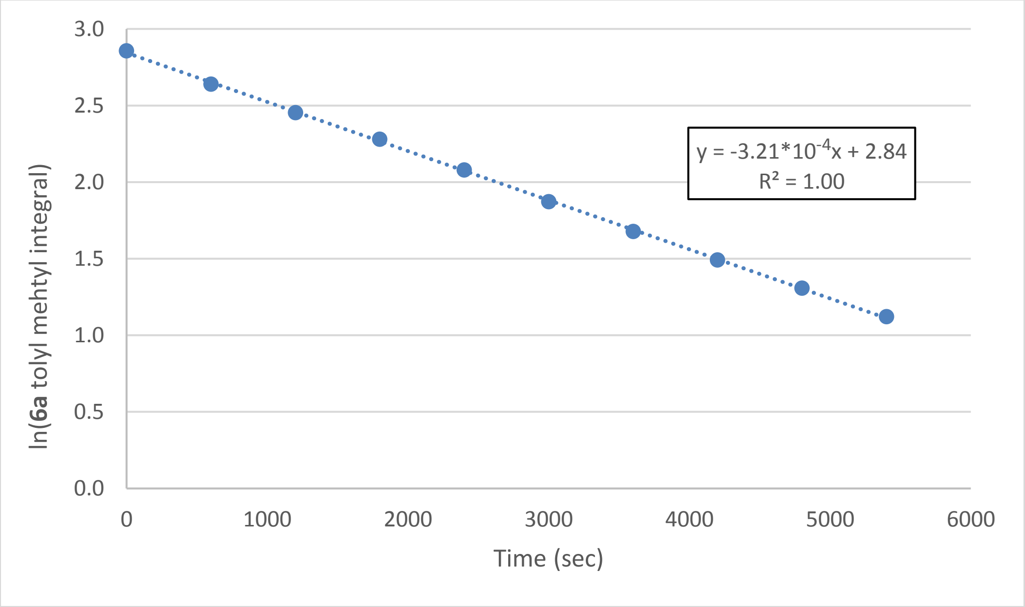

Figure S1. Plot of ln(**6a** integral) versus time.

Thermolysis of 6b. **6b** (40 mg, 0.057 mmol) was dissolved in 600 µL of C_6D_6 in a J. Young tube. A ^{1}H NMR spectrum of the mixture was acquired and then the NMR tube was placed in an 80 °C oil bath overnight. The final mixture contained: **2b, 3b**, and **D** in a 1.0:1.0:1.0 ratio as observed by ^{1}H and ^{19}F NMR spectroscopy.

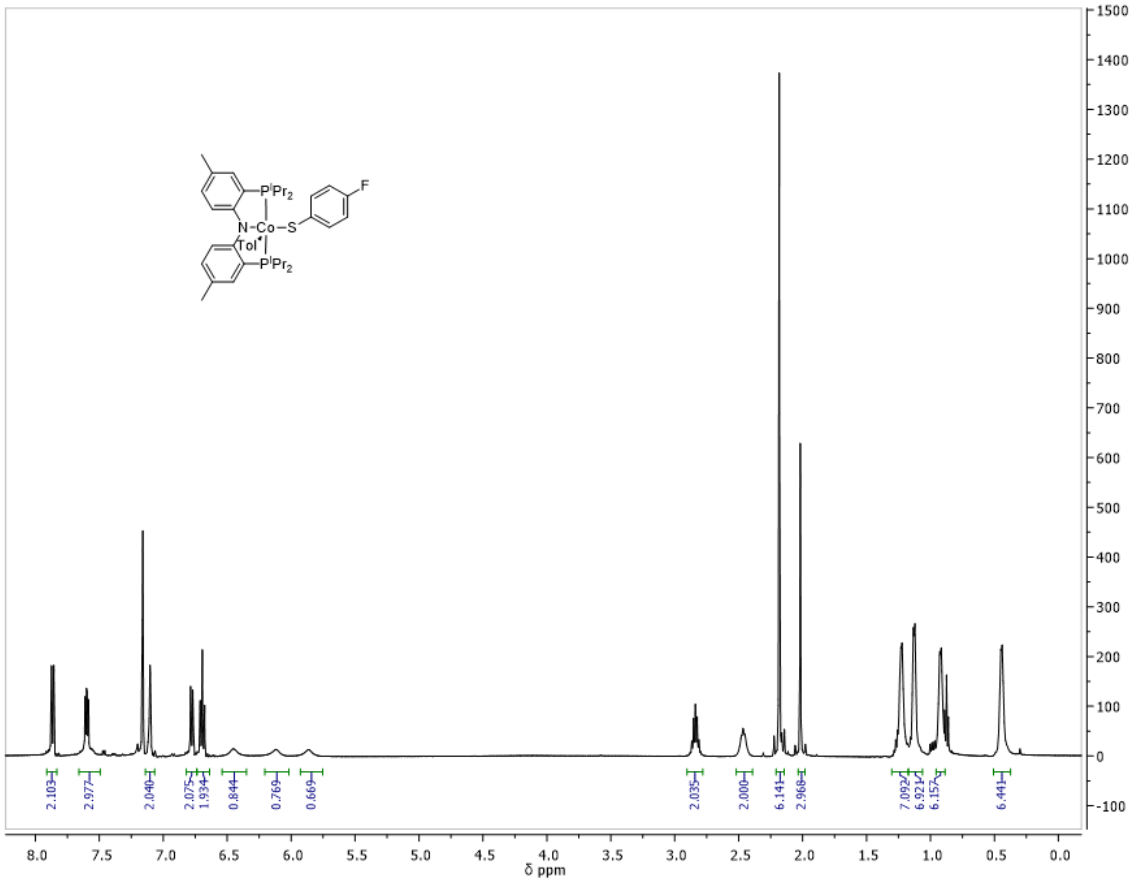

Figure S2. ^{1}H NMR (500 MHz, C_6D_6) spectrum of **6b** before thermolysis.

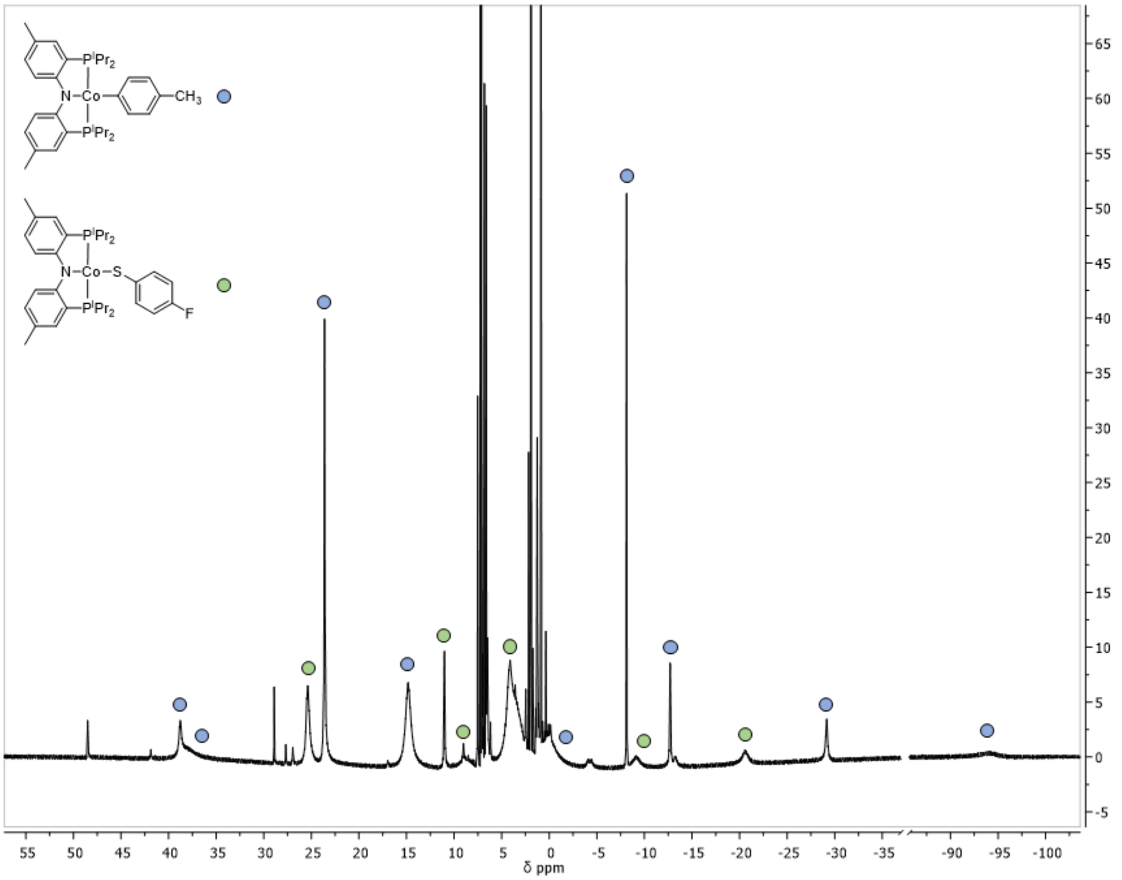

Figure S3. ^{1}H NMR (500 MHz, C_6D_6) of the thermolyzed **6b.**

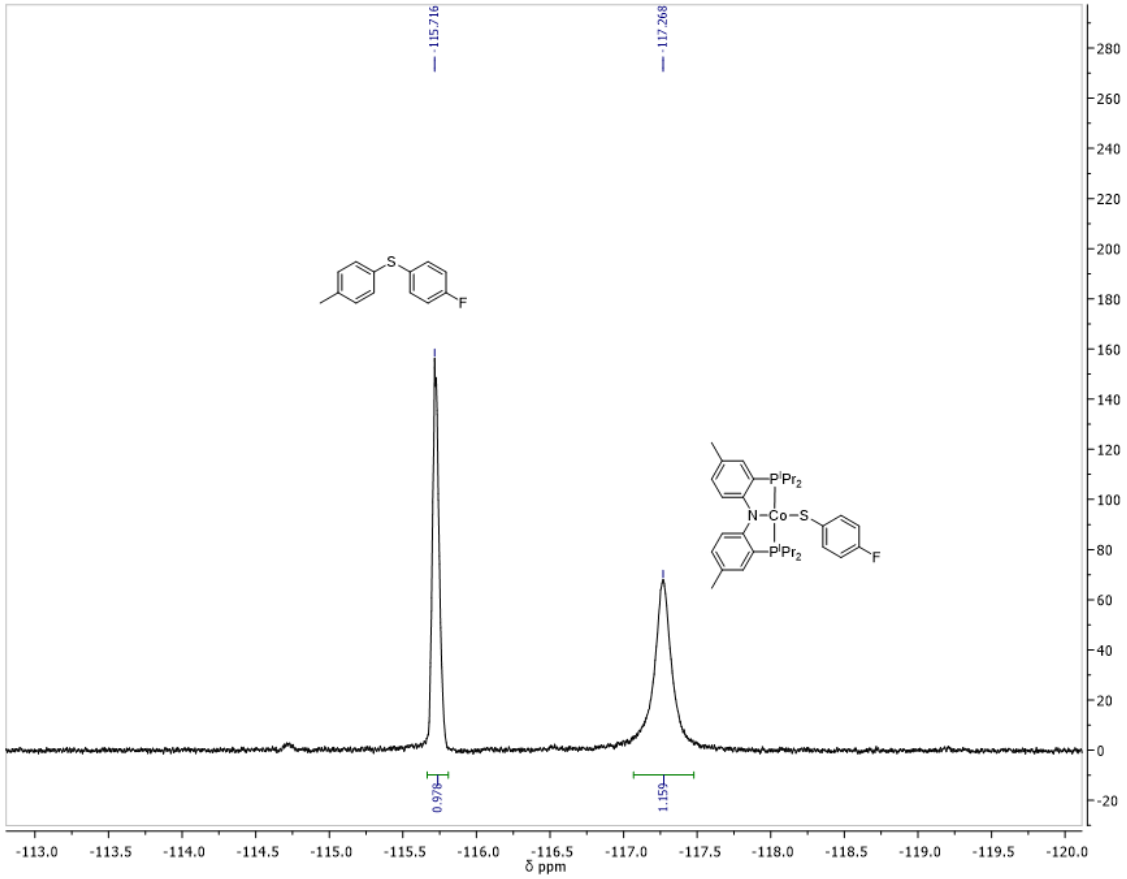

Figure S4. ^{19}F NMR (470 MHz, C_6D_6) of the thermolyzed **6b.**

Thermolysis of 6a with BHT as a radical inhibitor. **6a** (4 mg, 0.006 mmol) and 2,6-di-*tert*-butyl-4-methylphenol (BHT) (2 mg, 0.009 mmol) were dissolved in 600 μL of C_6D_6 in a J. Young tube. To this solution, 1.4-dioxane (2 μL, 0.023 mmol) was added using a syringe to serve as an internal standard. A ^{1}H NMR spectrum of the mixture was acquired and then the NMR tube was placed in an 80 °C oil bath for three hours. The final mixture contained: **2a** (46% of initial **6a**), **3a** (46% of initial **6a**) and **A** (45% of initial **6a**) as observed by ^{1}H NMR spectroscopy.

Thermolysis of a 1:1 mixture of 6a & 6b. In an Ar-filled glove box, a J. Young tube was charged with C_6D_6 (200 μL), **6a** (100 μL of a 0.15 M solution in C_6D_6, 0.015 mmol), **6b** (100 μL of a 0.14 M solution in C_6D_6, 0.014 mmol), and benzotrifluoride (100 μL of a 0.12 M solution in C_6D_6, 0.012 mmol) as a ^{19}F NMR referencing standard. The solution was then heated to 80 °C in an NMR spectrometer. ^{1}H and ^{19}F NMR spectra were acquired every ten minutes. *In situ* variable temperature ^{19}F NMR spectroscopy revealed the formation of **3b**, **D**, **C**, and the scrambled Co^{III}: **6c**. After thermolysis, ^{1}H NMR spectroscopy revealed the four expected (PNP)Co(X) species: **2a**, **2b**, **3a**, and **3b.** The four diaryl sulfides: **A**, **B**, **C**, and **D** were observed by GC-FID. This experiment was repeated with double the concentration of the two Co(III) complexes and, consistent with the first experiment, scrambling of thiolates in the Co^{III} complexes occurred, and the same distribution of final products were observed. Note: Figure S6 shows a ^{19}F NMR spectrum of a mixture of independently prepared **6b** and **6c**, showing that these two compounds give two resolved signals, as they do in the thermolysis reaction mixture.

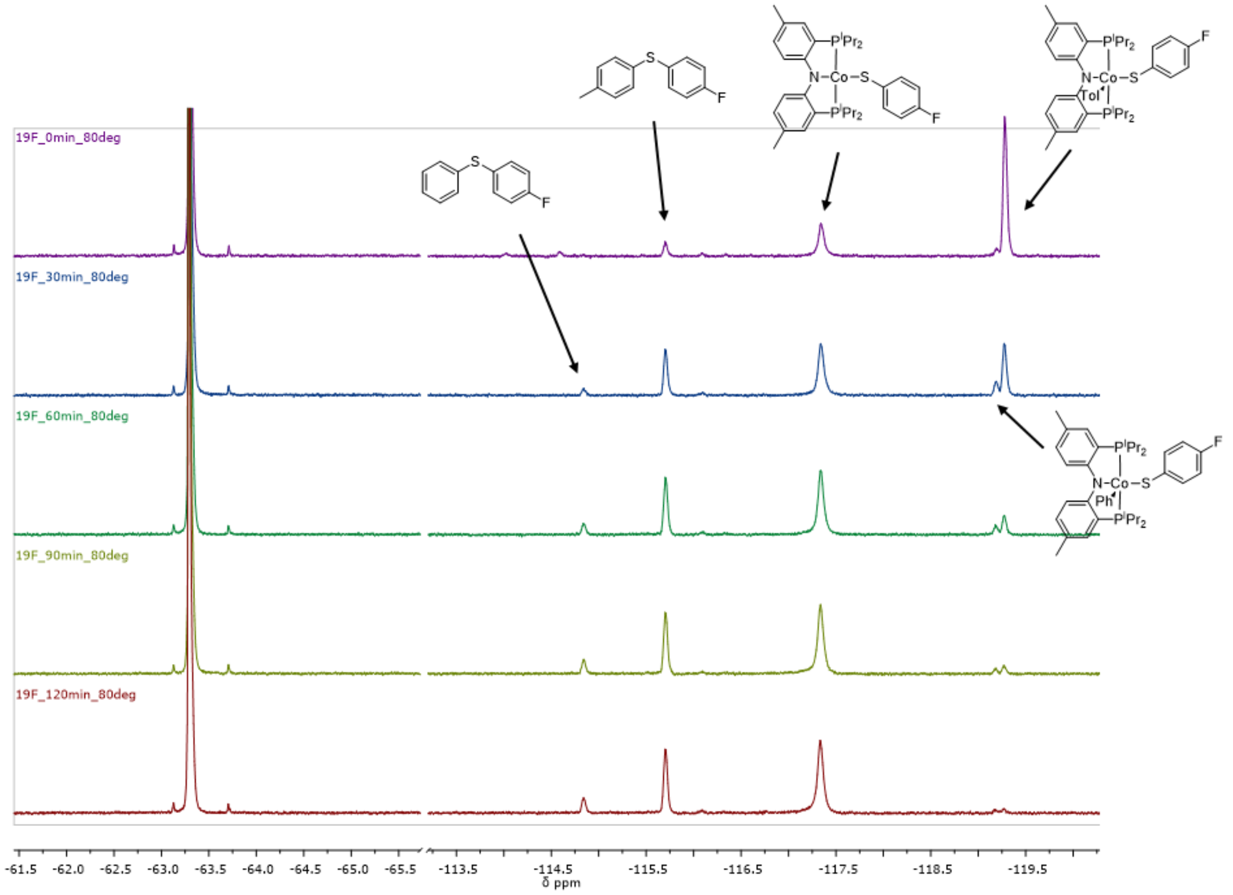

Figure S5. Variable temperature ^{19}F NMR (470 MHz, C_6D_6) observation of the mixed thermolysis of **6a** with **6b** (29 mM and 28 mM initial concentrations respectively). Timepoints shown are at 30 minute intervals.

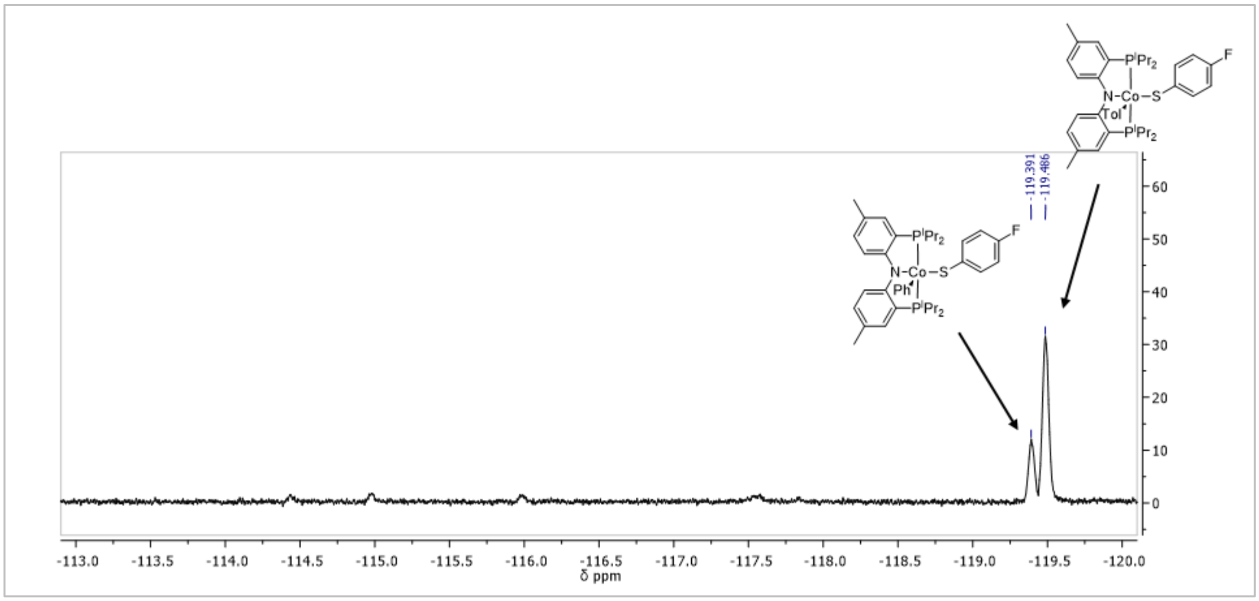

Figure S6. ^{19}F NMR (470 MHz, C_6D_6) of a pure sample of **6b** spiked with **6c**.

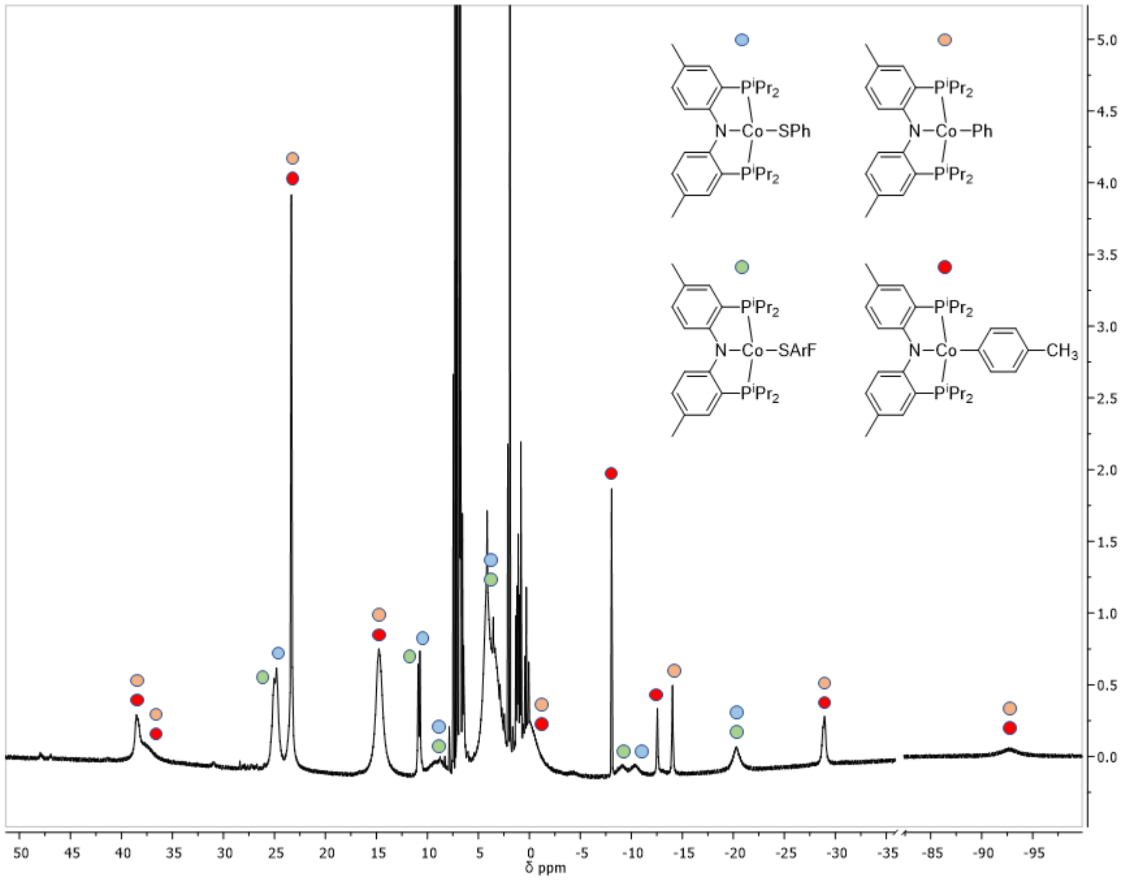

Figure S7. ^{1}H NMR (500 MHz, C_6D_6) of the mixed thermolysis of **6a** with **6b** after cooling to room temperature. The four expected Co(II) products are observed.

Thermolysis of 6b with A. In an Ar-filled glove box, a J. Young tube was charged with **6b** (200 μL of a 0.14 M solution in C_6D_6, 0.028 mmol), and **A** (10 μL, 0.059 mmol) then diluted with C_6D_6. The mixture was thermolyzed in an 80 °C oil bath for two days. **C**, and **D** were both observed by ^{19}F NMR spectroscopy. **C** constituted approximately 2% of the fluorinated diarylsulfides that were formed.

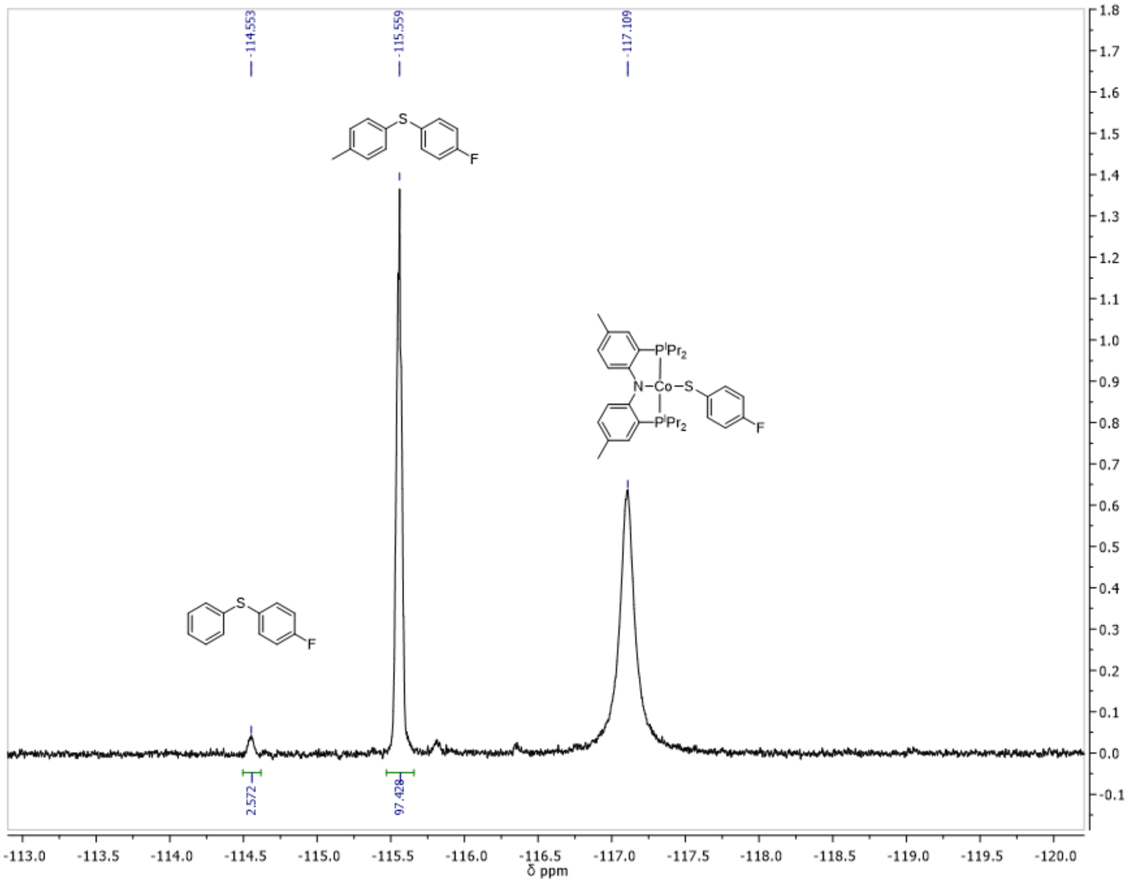

Figure S8. ^{19}F NMR (470 MHz, C_6D_6) spectrum showing the resultant mixture of thermolysis of **6b** with **A**.

Thermolysis of 6a with 3b. In an Ar-filled glovebox, a J. Young tube was charged with **6a** (18 mg, 0.027 mmol) and **3b** (17 mg, 0.028 mmol) then diluted with C_6D_6. This mixture was heated to 80 °C overnight. ^{1}H NMR spectroscopy revealed the formation of **3a**, and **2a** in a 1.0:0.95 ratio in addition to **3b**. ^{19}F NMR spectroscopy revealed the formation of **C** containing 6% of the total available fluorinated thiolate indicating that Co^{II} and Co^{III} complexes can exchange thiolate ligands.

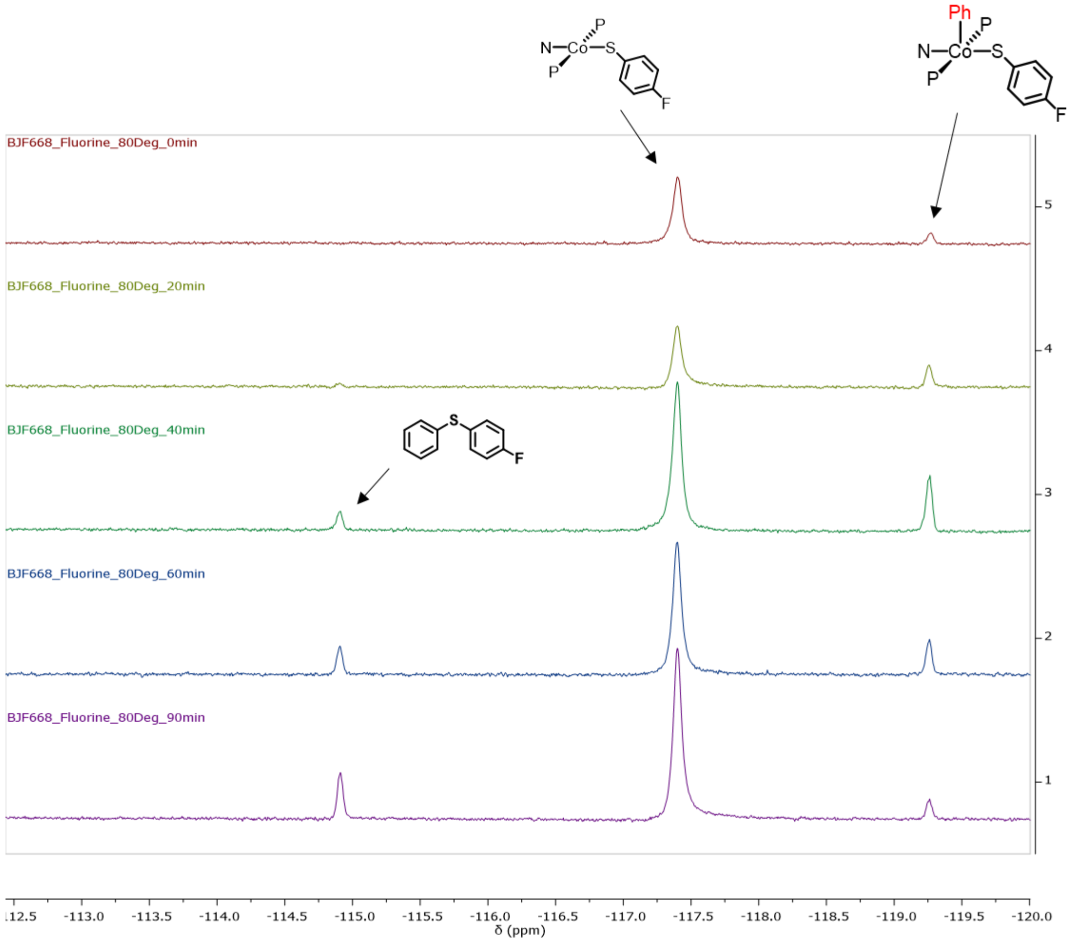

Figure S9. ^{19}F NMR (470 MHz, C_6D_6) *in situ* spectra of thermolysis of a 1:1 mixture of **6a** and **3b**.

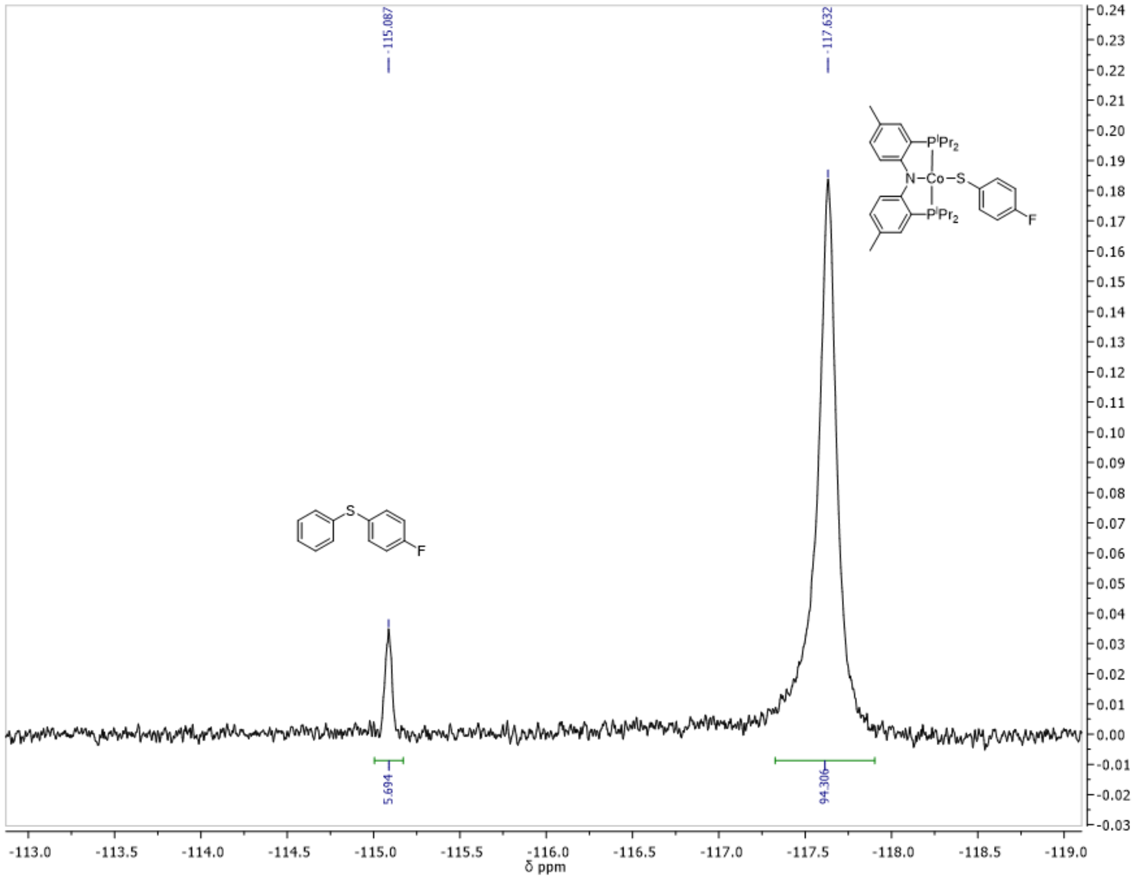

Figure S10. ^{19}F NMR (470 MHz, C_6D_6) after thermolysis of **6a** in the presence of **3b** showing the fluorinated diarylsulfide **C**.

Thermolysis of 2b and 3b with A. In an Ar-filled glove box, a J. Young tube was charged with **2b** (21mg, 0.036 mmol), **3b** (21 mg, 0.035 mmol), and **A** (16 µL, 0.096 mmol) then diluted with C_6D_6. The mixture was thermolyzed in an 80 °C oil bath for seven days. Only **2b**, **3b**, and **A** were observed by ^1H and ^{19}F NMR spectroscopy indicating that Co^{II} complexes cannot activate diaryl sulfides.

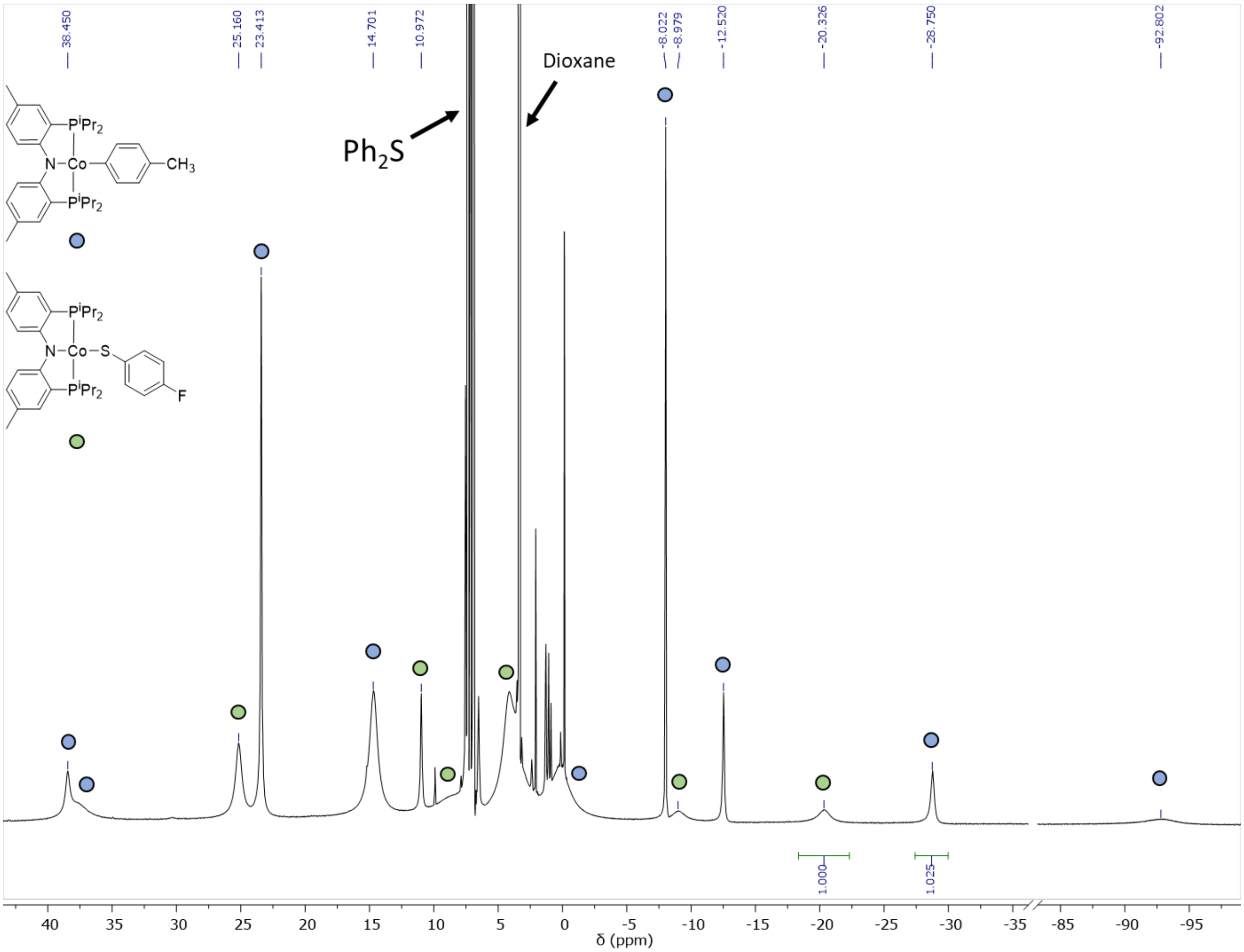

Figure S11. ^1H NMR (400 MHz, C_6D_6) spectrum of the seven-day thermolysis of **2b**, **2b**, and **A**. Only the starting materials are observed.

V. Reactions of Co (I) Compounds

Reaction of 7 with 6a. In an Ar-filled glove box, a J. Young tube was charged with **7** (23 mg, 0.047 mmol (PNP)Co) and **6a** (50 mg, 0.074 mmol). The solids were dissolved with C_6D_6. An immediate color change was observed upon mixing resulting in a green solution indicative of $(PNP)Co^{II}$ complexes. ^1H NMR spectroscopy revealed the formation of **2a** and **3a** in a 1.0:1.0 ratio.

Reaction of 8 with 6a. In an Ar-filled glove box, a J. Young tube was charged with $(N(TMS)_2)Co(PPh_3)_2$ (17 mg, 0.022 mmol) and $^{Me}PN^{H}P^{iPr}$ (11 mg, 0.025 mmol) in C_6D_6. A ^1H NMR spectrum was acquired showing formation of **8**. **6a** (50 mg, 0.074 mmol) was then added and an immediate color change was observed upon mixing resulting in a green solution indicative of $(PNP)Co^{II}$ complexes. ^1H NMR spectroscopy revealed the formation of **2a** and **3a** in a 1.0:1.0 ratio.

Reaction of 7 with PPh_3 and subsequent addition of tris(4-methoxyphenyl)phosphine. In an Ar-filled glove box, a J. Young tube was charged with **7** (20 mg, 0.042 mmol (PNP)Co) and triphenylphosphine (131 mg, 0.50 mmol) then diluted with C_6D_6. The solution was heated to 55 °C overnight resulting in complete conversion of the dimer to **8**. The tube was then brought back into the glove box and tris(4-methoxyphenyl)phosphine (176 mg, 0.50 mmol) was added. ^1H NMR spectroscopy revealed a second set of paramagnetically shifted resonances indicating that the phosphine is associated with the metal center.

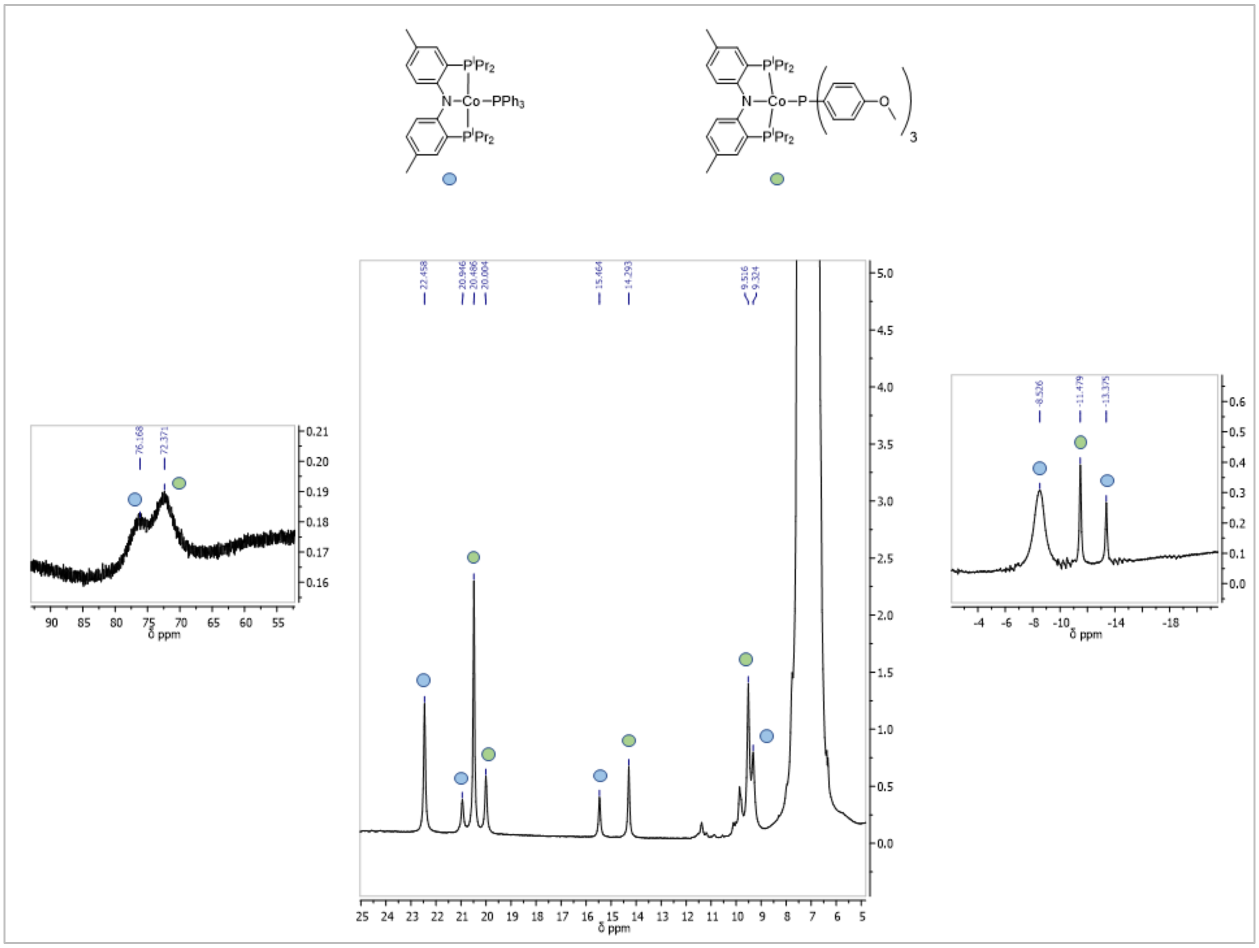

Figure S12. ^{1}H NMR (500 MHz, C_6D_6) of **7** after adding PPh_3 and then tris(4-methoxyphenyl)phosphine showing closely related paramagnetically shifted ^{1}H NMR resonances.

Reaction of 7 with A. In an Ar-filled glove box, a J. Young tube was charged with **7** (23 mg, 0.046 mmol (PNP)Co) and **A** (18 µL, 0.10 mmol) then diluted with C_6D_6. The solution was heated at 55 °C overnight resulting in consumption of the **7** and formation of **3a** and **2a** in a 1.0:1.1 ratio as observed by ^1H NMR spectroscopy.

Reaction of 7 with E. In an Ar-filled glove box, a J. Young tube was charged with **7** (23 mg, 0.046 mmol (PNP)Co) and **E** (125 µL of 0.40 M stock solution in C_6D_6, 0.05 mmol). The solution was heated in a 55 ^{O}C oil bath overnight resulting in little change by ^1H, and ^{19}F NMR spectroscopy. The solution was then heated in an 80 ^{o}C oil bath for three days resulting in complete consumption of **7**. Two paramagnetic products were identified by ^1H and ^{19}F NMR spectroscopy as **2c** and **3c** in a 1:0.6 ratio. No (PNP)Co(S-4-C_6H_4F) was observed by ^1H nor ^{19}F NMR spectroscopy.

The solution was treated with 50 µL of 2N HCl in diethyl ether after which, **1** and **2c** were observed as the major (PNP)Co^{II} complexes in solution in a 0.6:1 ratio by ^1H NMR spectroscopy. APCI-MS and GC-MS of the solution revealed only **E** and 2-isopropylthiophenol. No 4-fluorothiophenol was observed by these methods indicating that only the S–C^{ArF} bond was cleaved which is consistent with an oxidative addition C–S activation pathway.

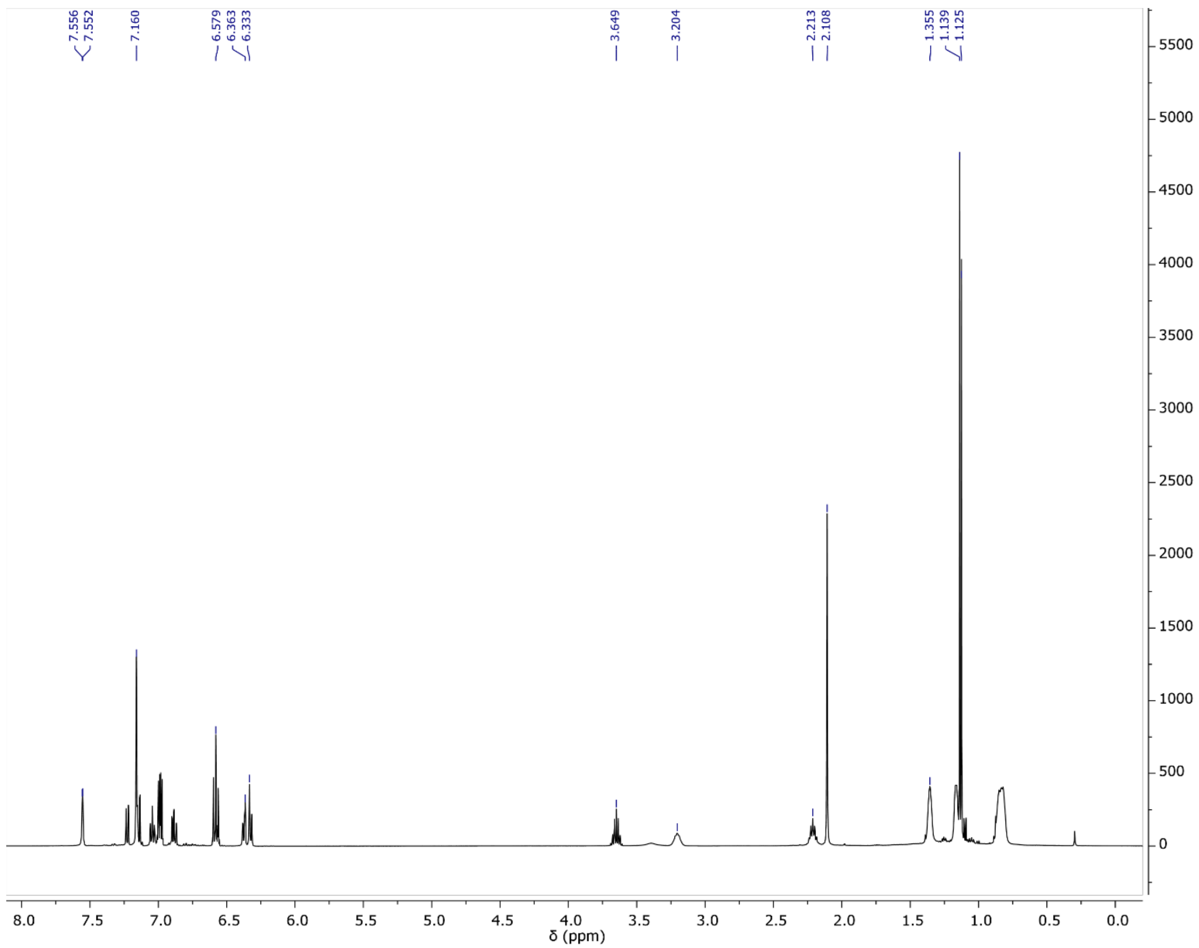

Figure S13. ^{1}H NMR (500 MHz, C_6D_6) of **7** and **E** before heating. Sample contains residual THF and silicone grease.

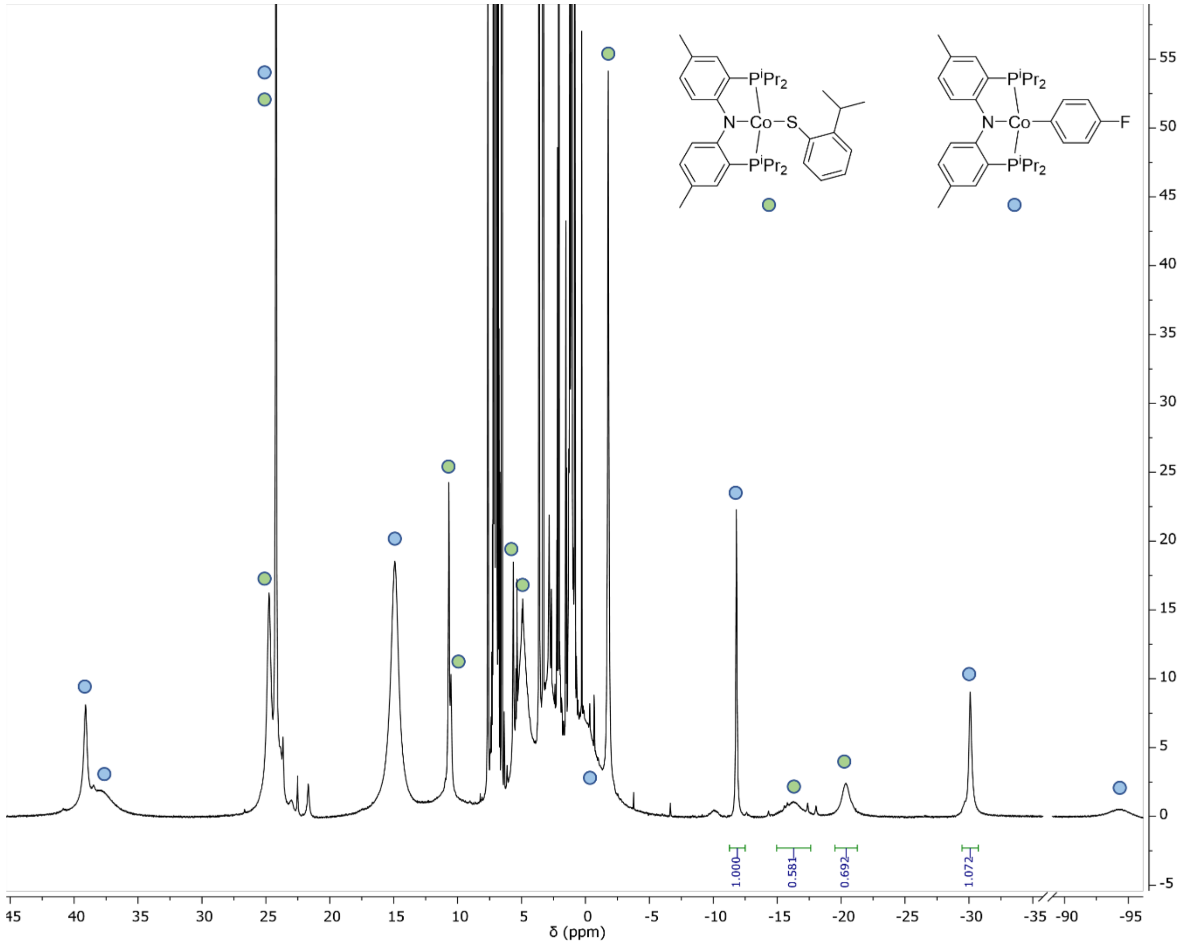

Figure S14. ^{1}H NMR (500 MHz, C_6D_6) of **7** and **E** after heating at 80 °C for three days.

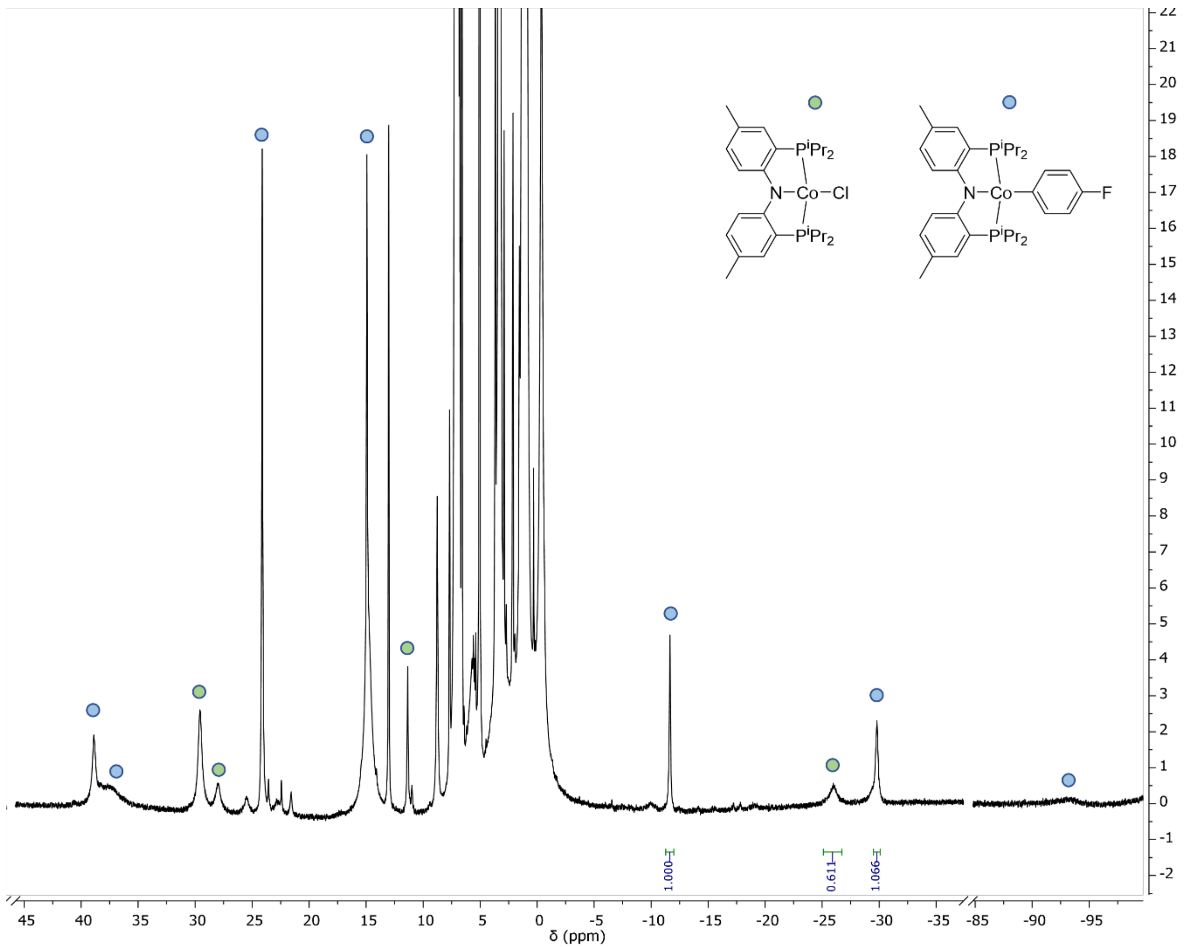

Figure S15. ^{1}H NMR (500 MHz, C_6D_6) of **7** and **E** after heating at 80 °C for three days and then treating with anhydrous HCl in diethyl ether.

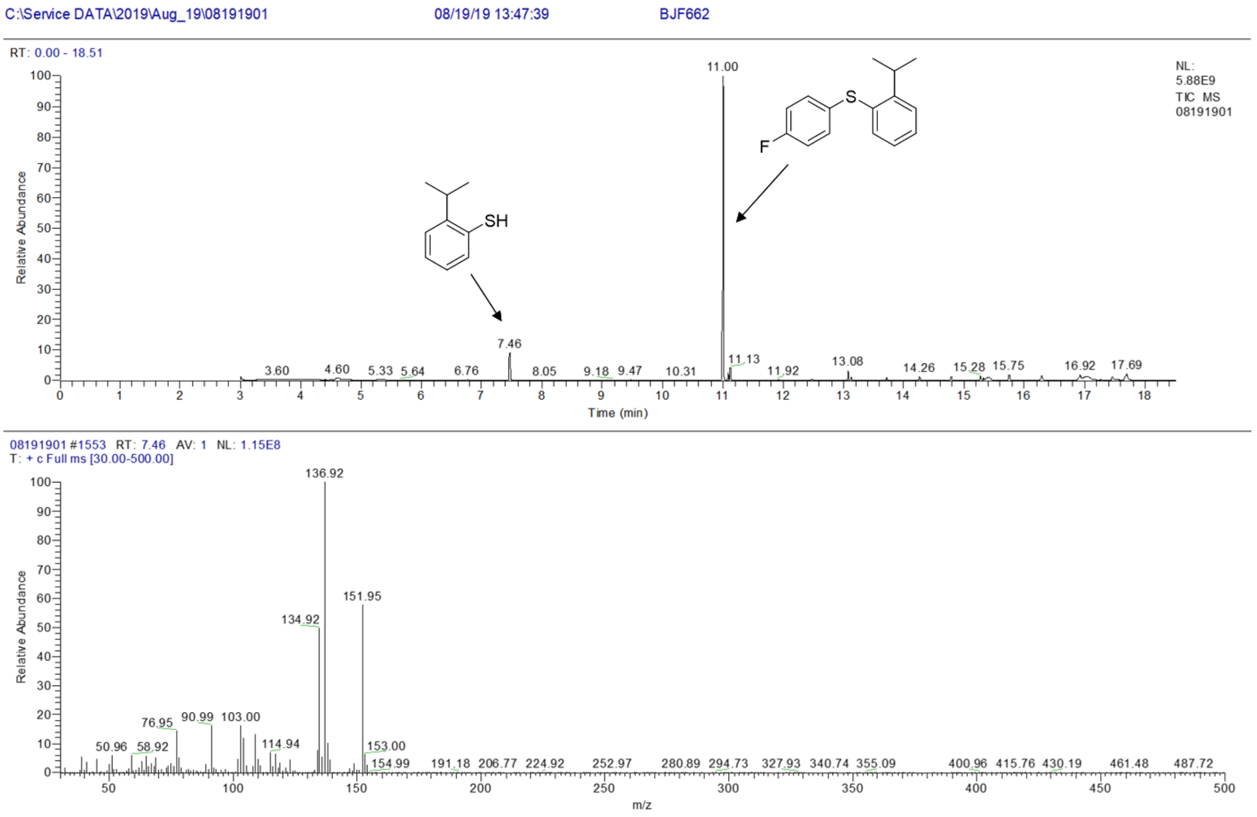

Figure S16. GC-MS of the reaction solution resulting from treatment with HCl. GC trace (top). Mass spectrum at a retention time of 7.46 minutes (bottom).

VI. NMR Spectra & GC Chromatograms

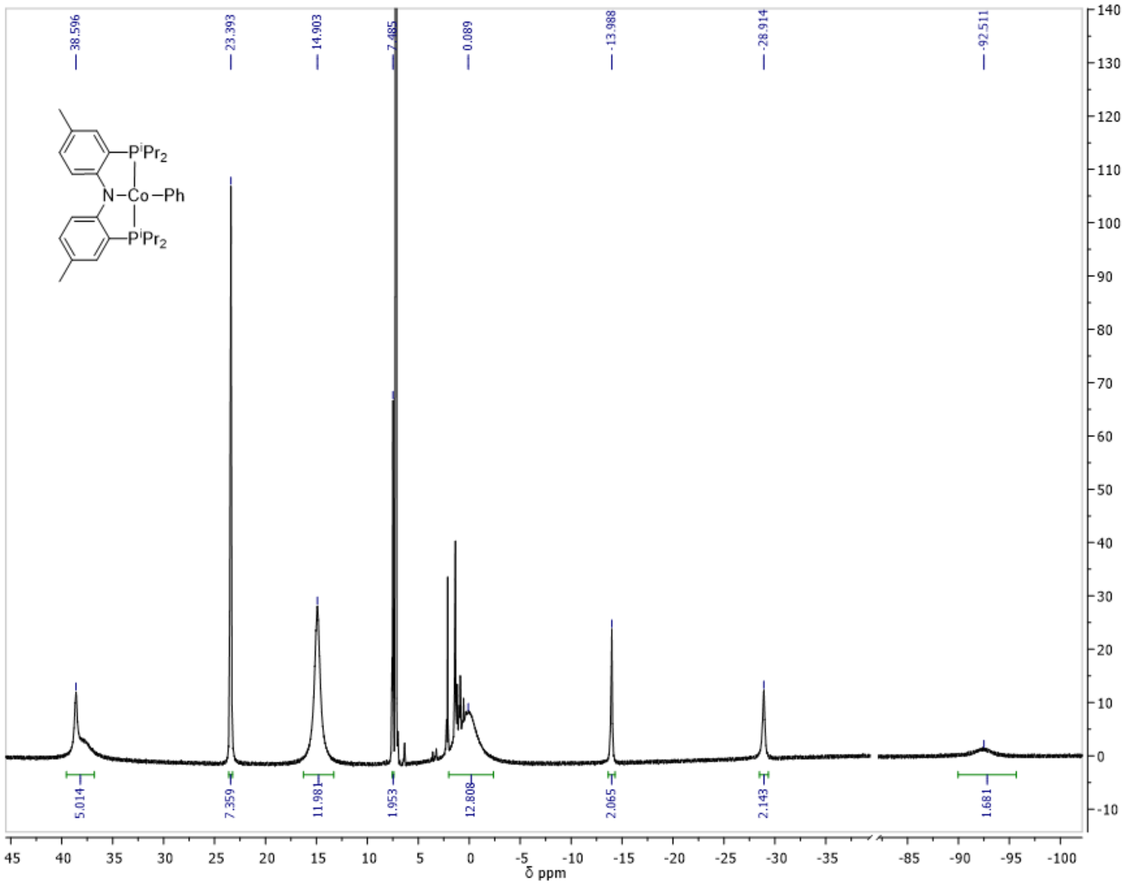

Figure S17. ^{1}H NMR (500 MHz, C_6D_6) spectrum of **2a**.

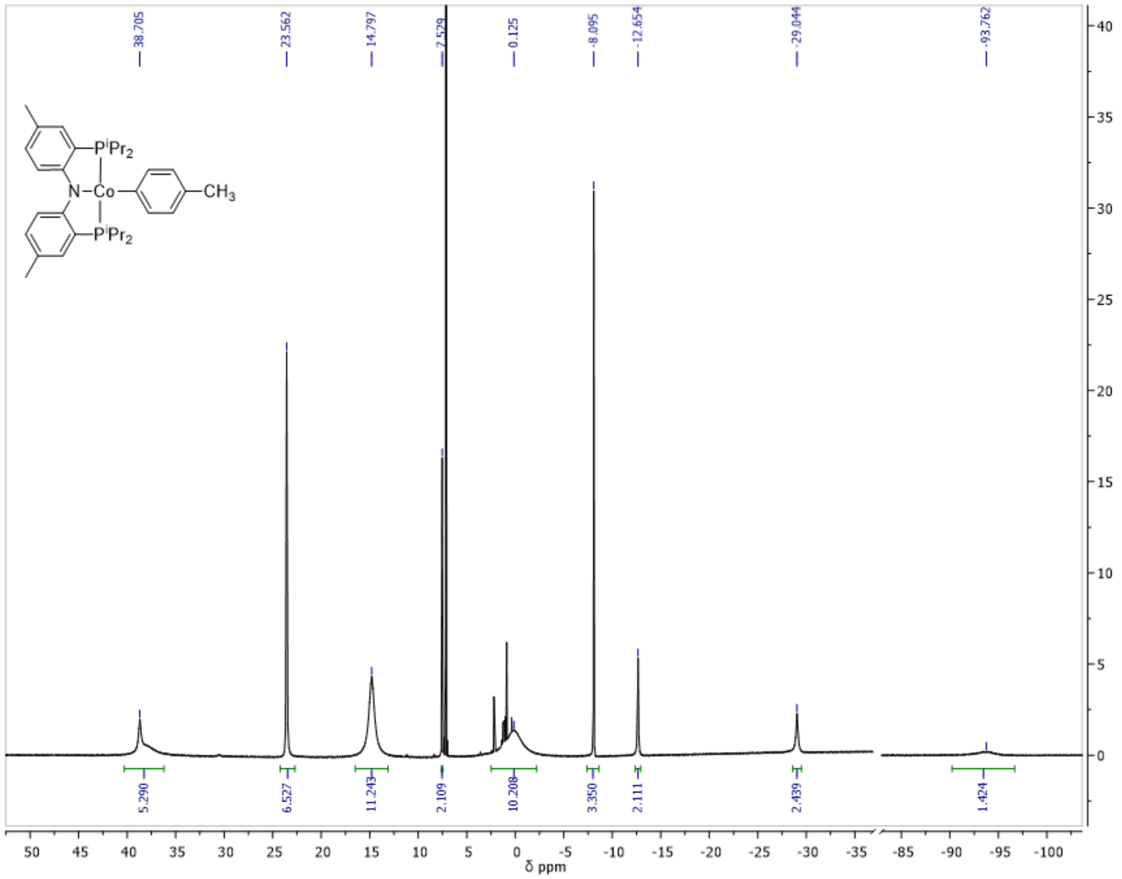

Figure S18. ^{1}H NMR (500 MHz, C_6D_6) spectrum of **2b**.

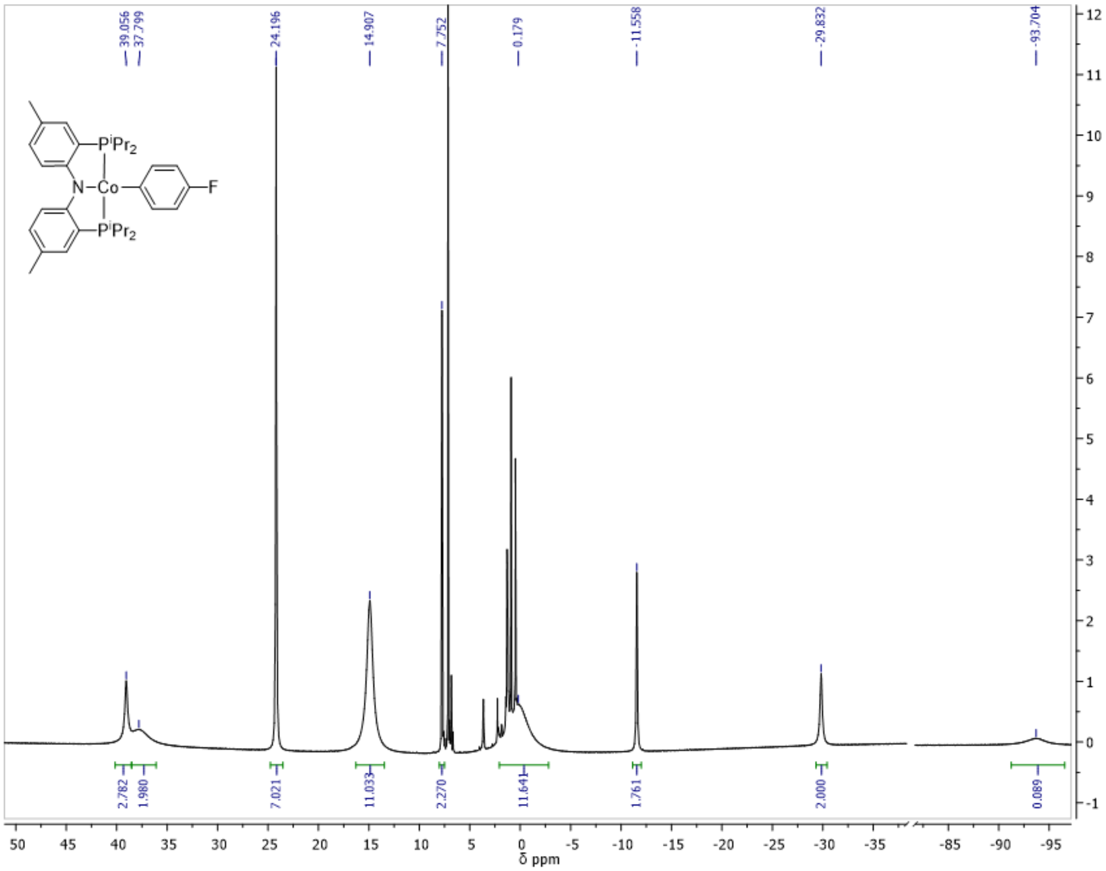

Figure S19. ^{1}H NMR (500 MHz, C_6D_6) spectrum of **2c.**

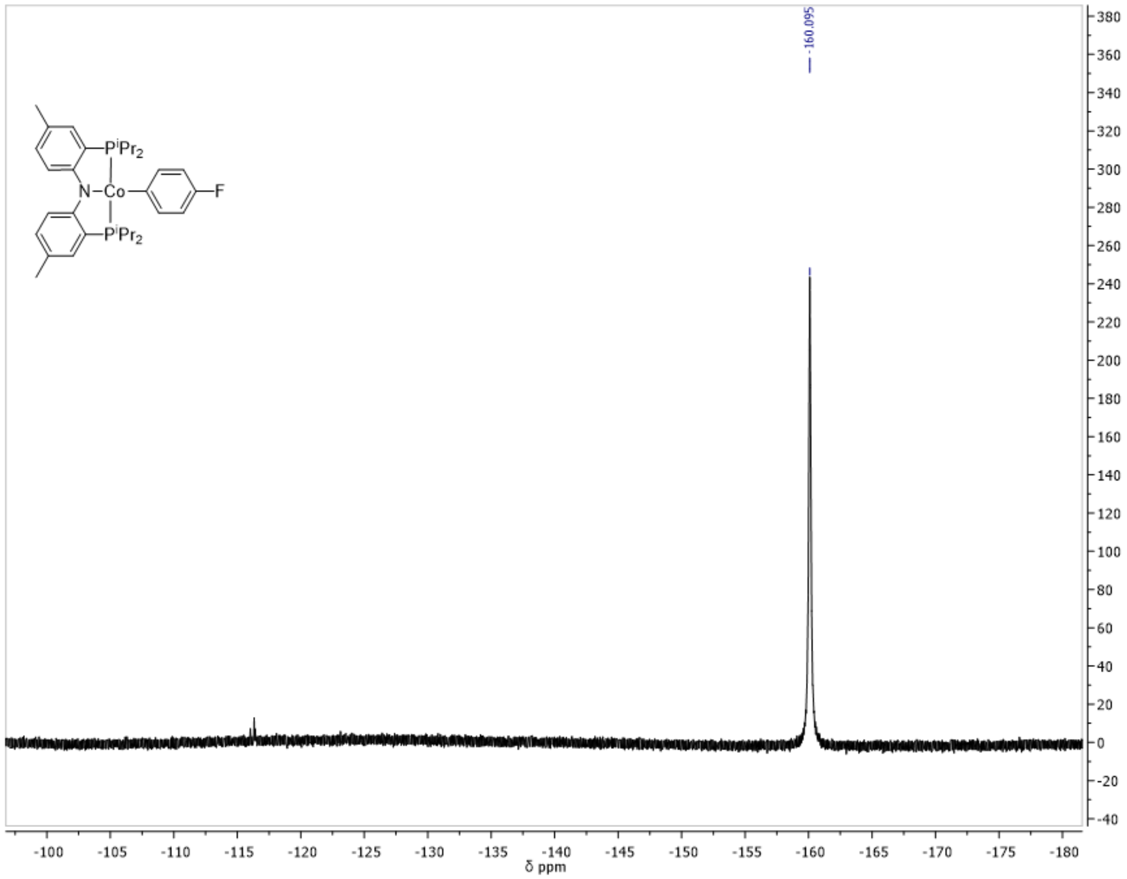

Figure S20. ^{19}F NMR (470 MHz, C_6D_6) spectrum of **2c**.

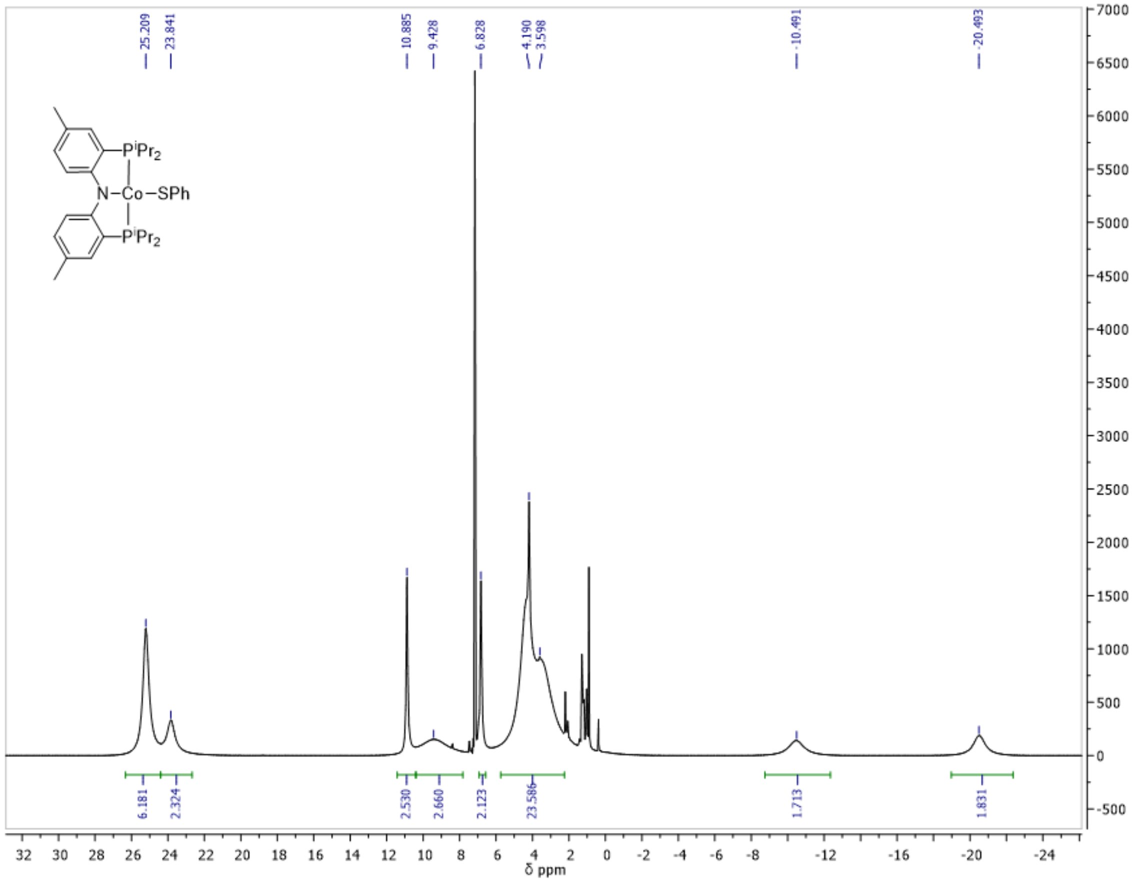

Figure S21. ^{1}H NMR (500 MHz, C_6D_6) spectrum of **3a**.

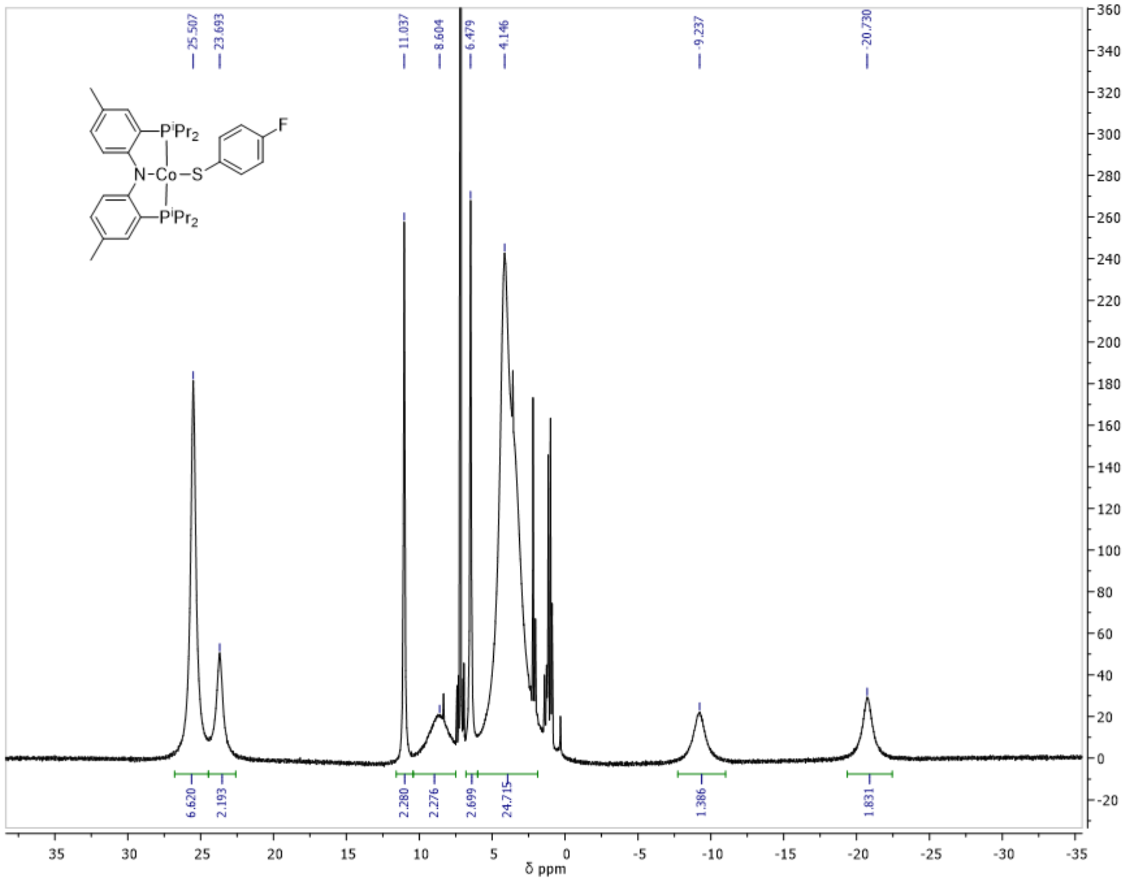

Figure S22. ^{1}H NMR (500 MHz, C_6D_6) spectrum of **3b**.

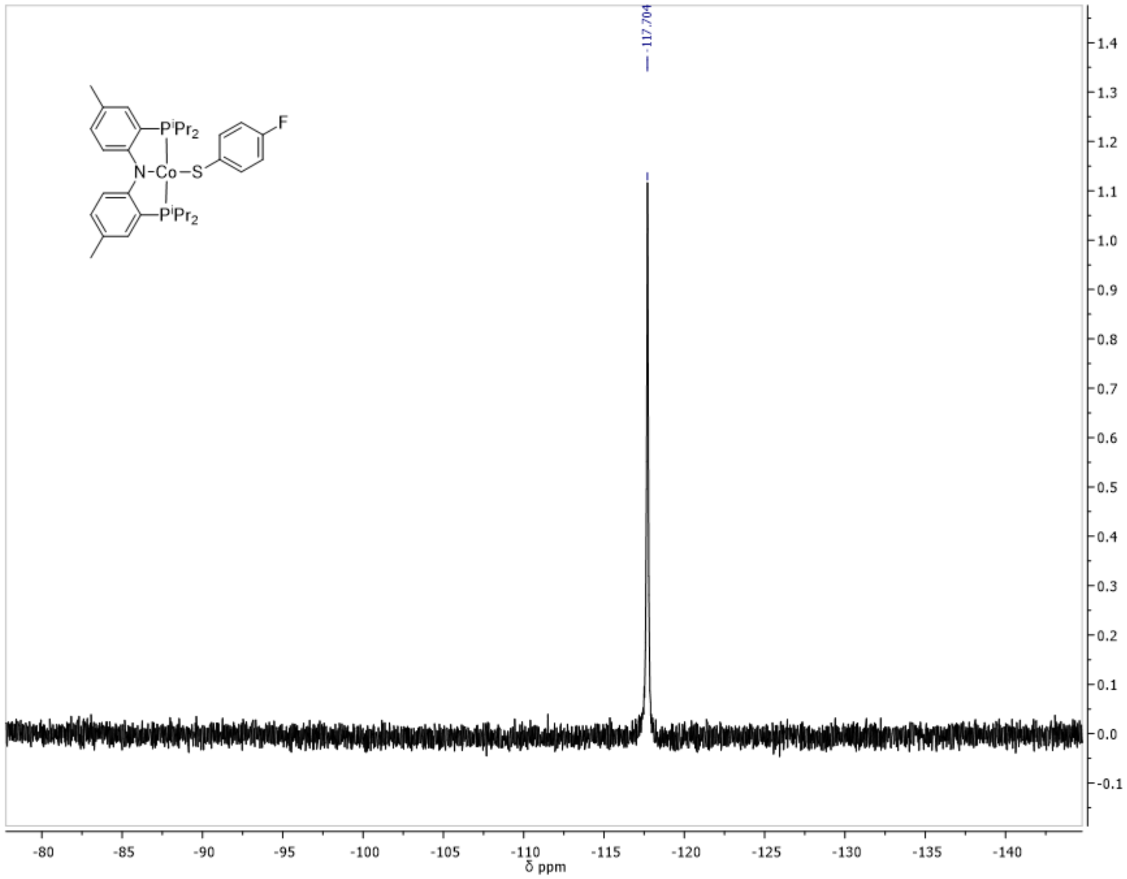

Figure S23. ^{19}F NMR (470 MHz, C_6D_6) spectrum of **3b**.

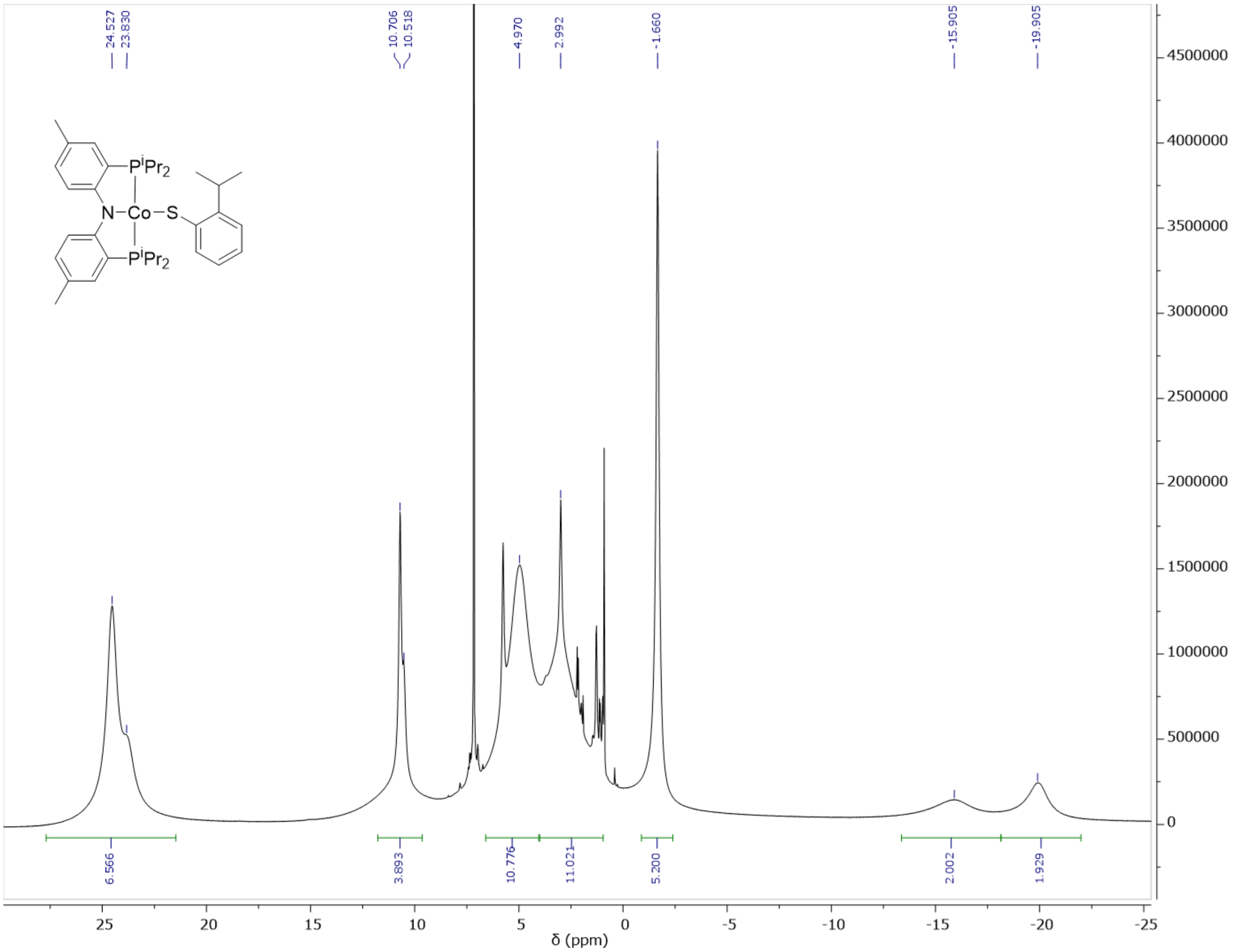

Figure S24. ^{1}H NMR (400 MHz, C_6D_6) spectrum of **3c**.

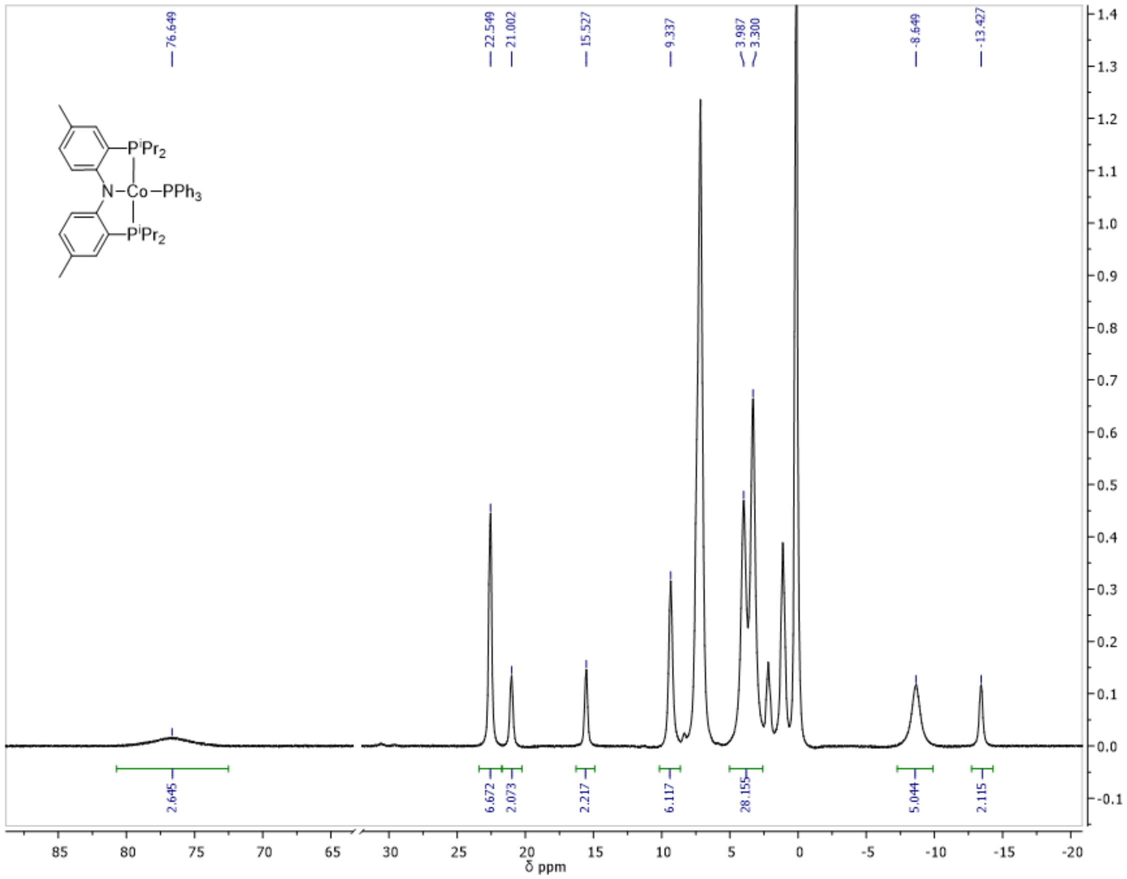

Figure S25. ^{1}H NMR (500 MHz, C_6D_6) spectrum of **8**. Sample contains triphenylphosphine and hexamethyldisilazane.

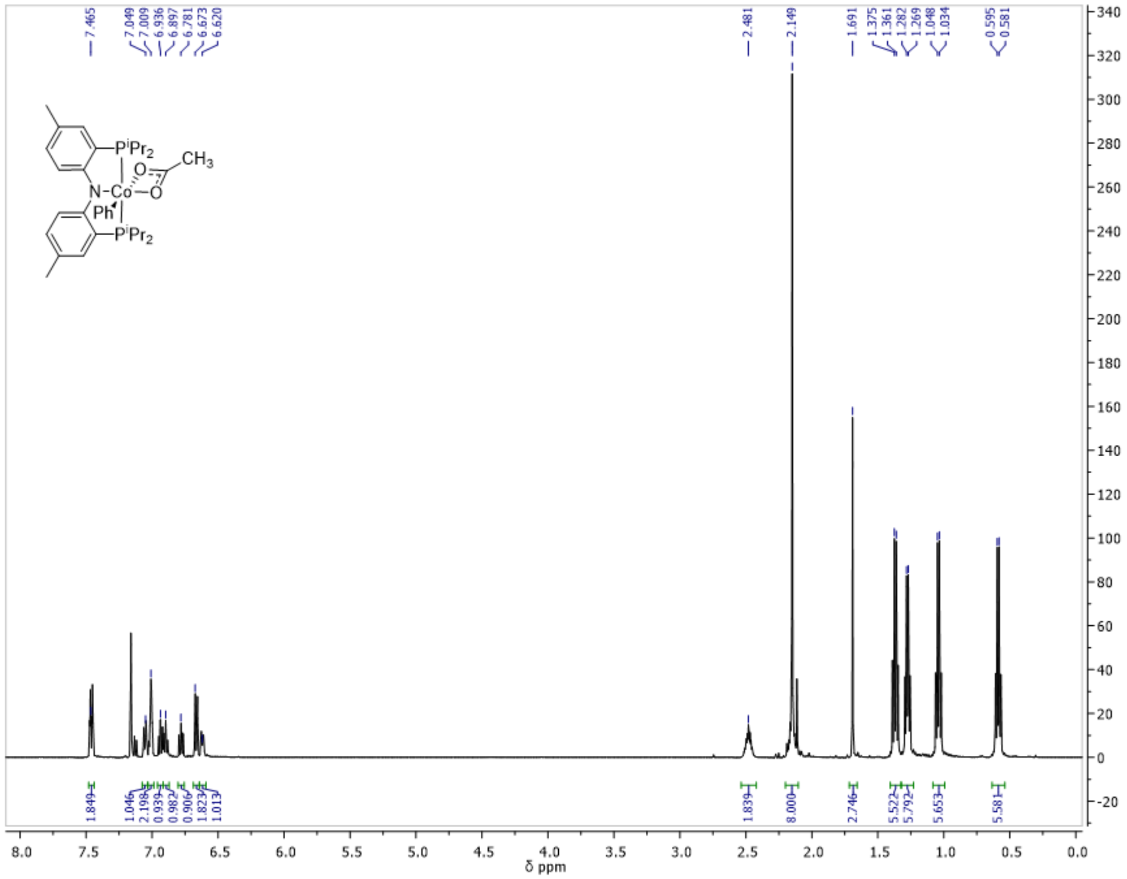

Figure S26. ^{1}H NMR (500 MHz, C_6D_6) spectrum of **4a**. Sample contains residual toluene.

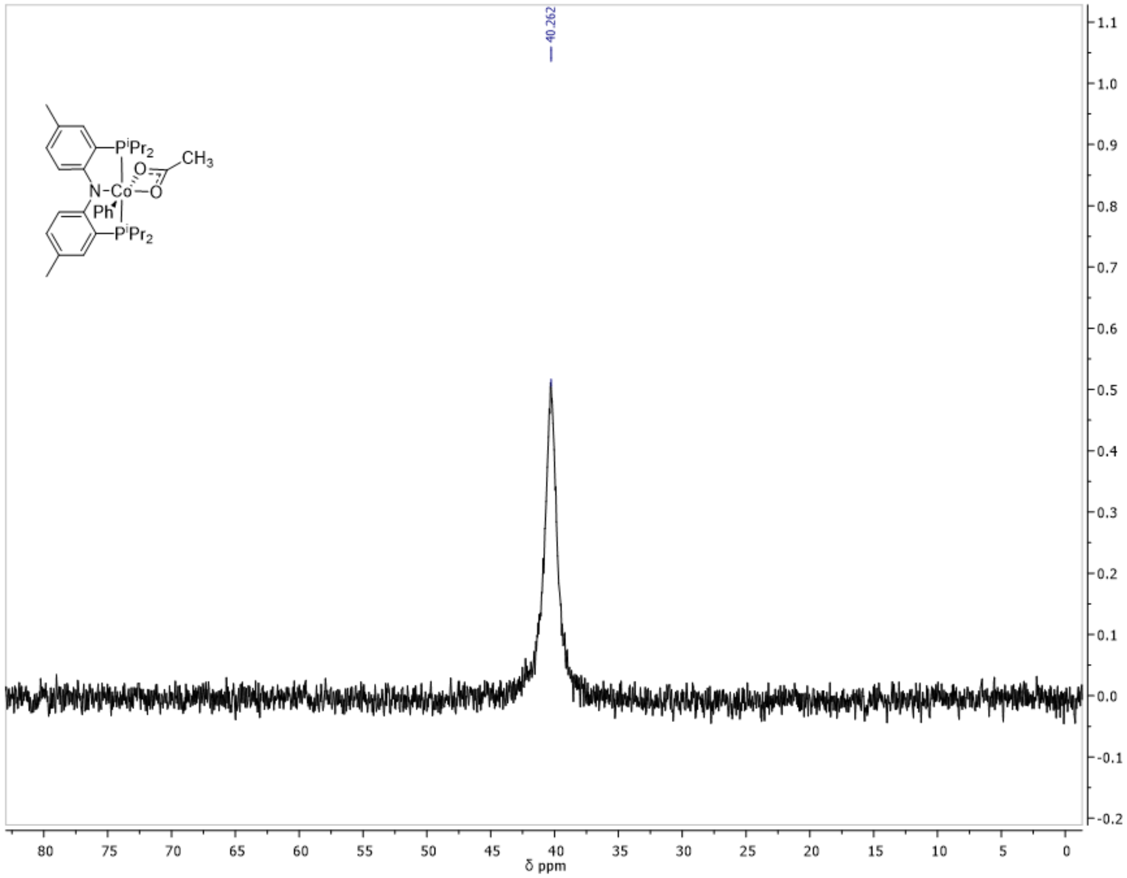

Figure S27. $^{31}P\{^1H\}$ NMR (202 MHz, C_6D_6) spectrum of **4a**.

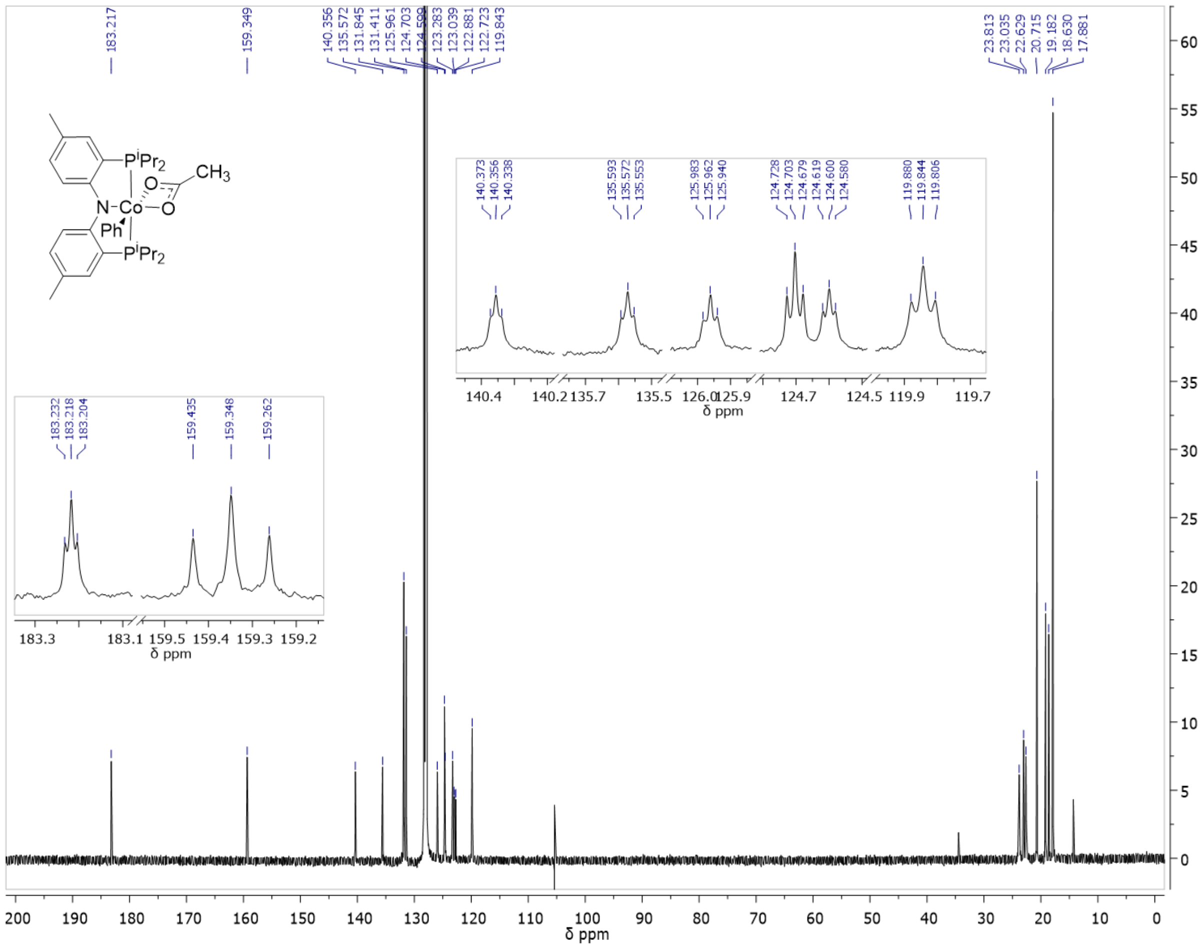

Figure S28. $^{13}C\{^1H\}$ NMR (125 MHz, C_6D_6) spectrum of **4a.** Pentane resonances not picked. Disturbance at 105 ppm is an artifact from the spectrometer. Insert Left: Expanded view of triplets downfield of 150 ppm. Insert Center: Expanded view of resonances between 119 and 141 ppm showing fine structure.

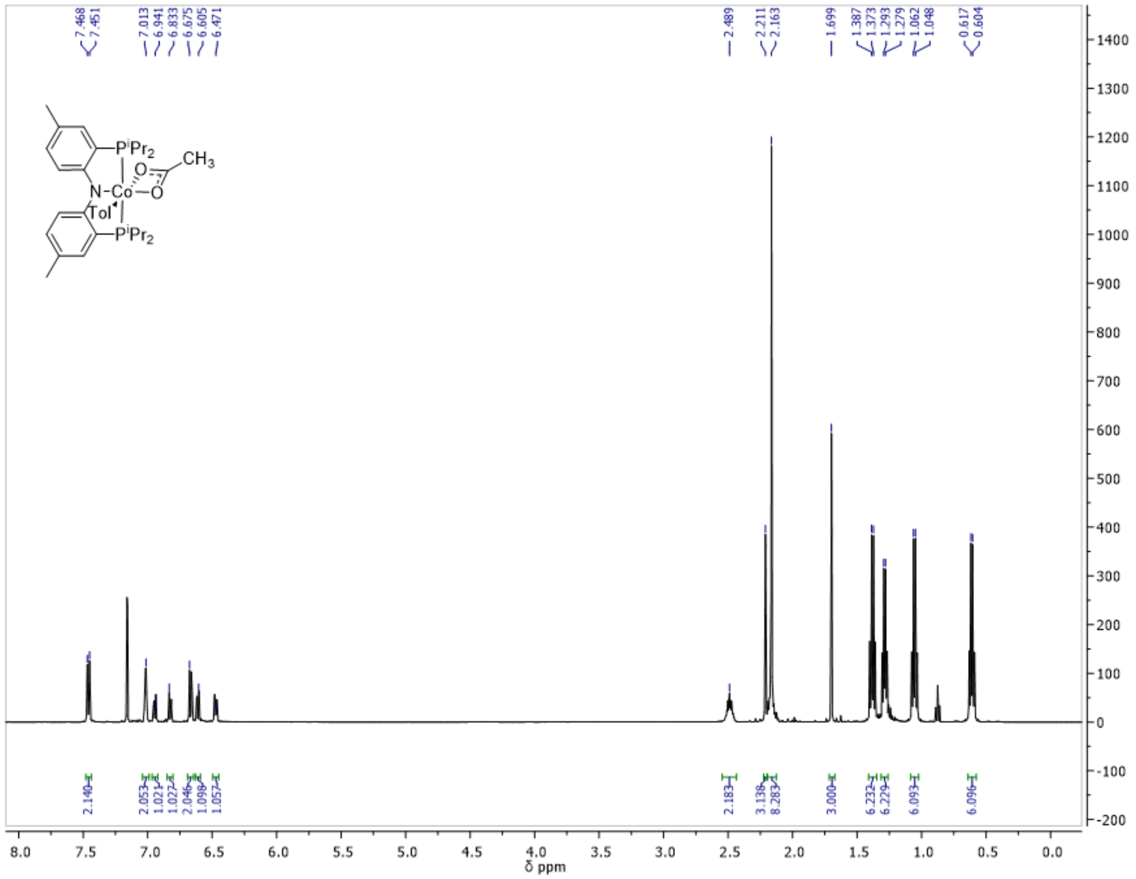

Figure S29. ^{1}H NMR (500 MHz, C_6D_6) spectrum of **4b**. Sample contains residual pentane.

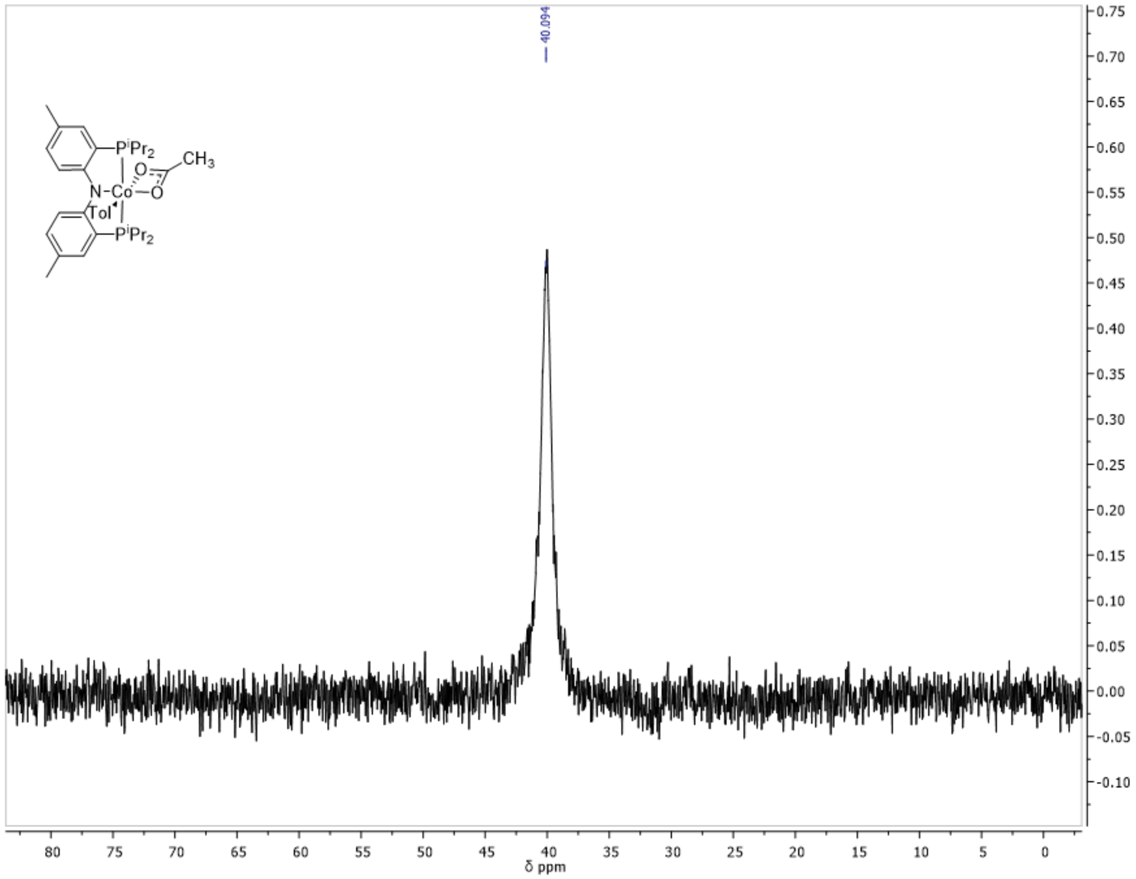

Figure S30. $^{31}P\{^1H\}$ NMR (202 MHz, C_6D_6) spectrum of **4b**.

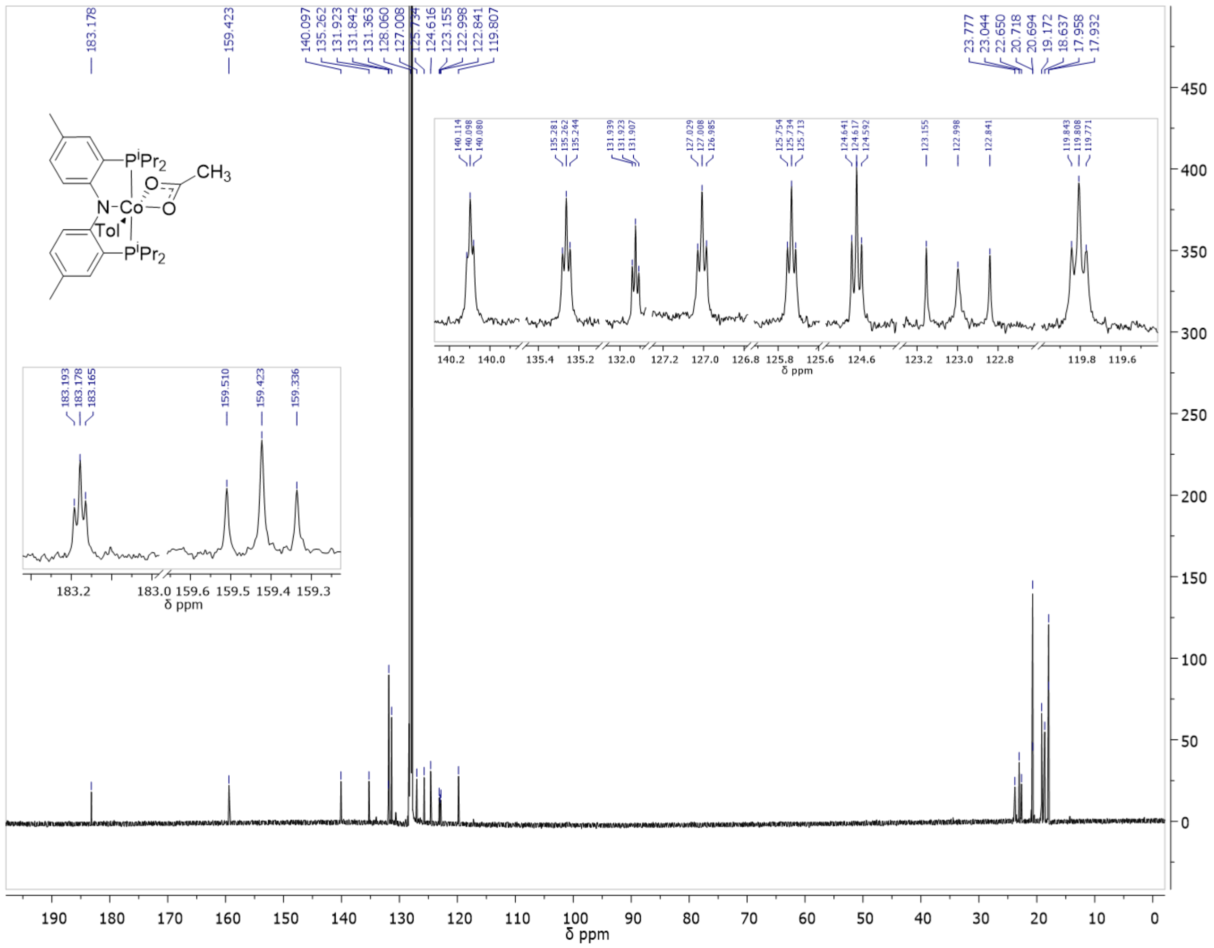

Figure S31. $^{13}C\{^1H\}$ NMR (125 MHz, C_6D_6) spectrum of **4b**. Insert Left: Expanded view of triplets downfield of 150 ppm. Insert Right: Expanded view of resonances between 119 and 141 ppm showing fine structure.

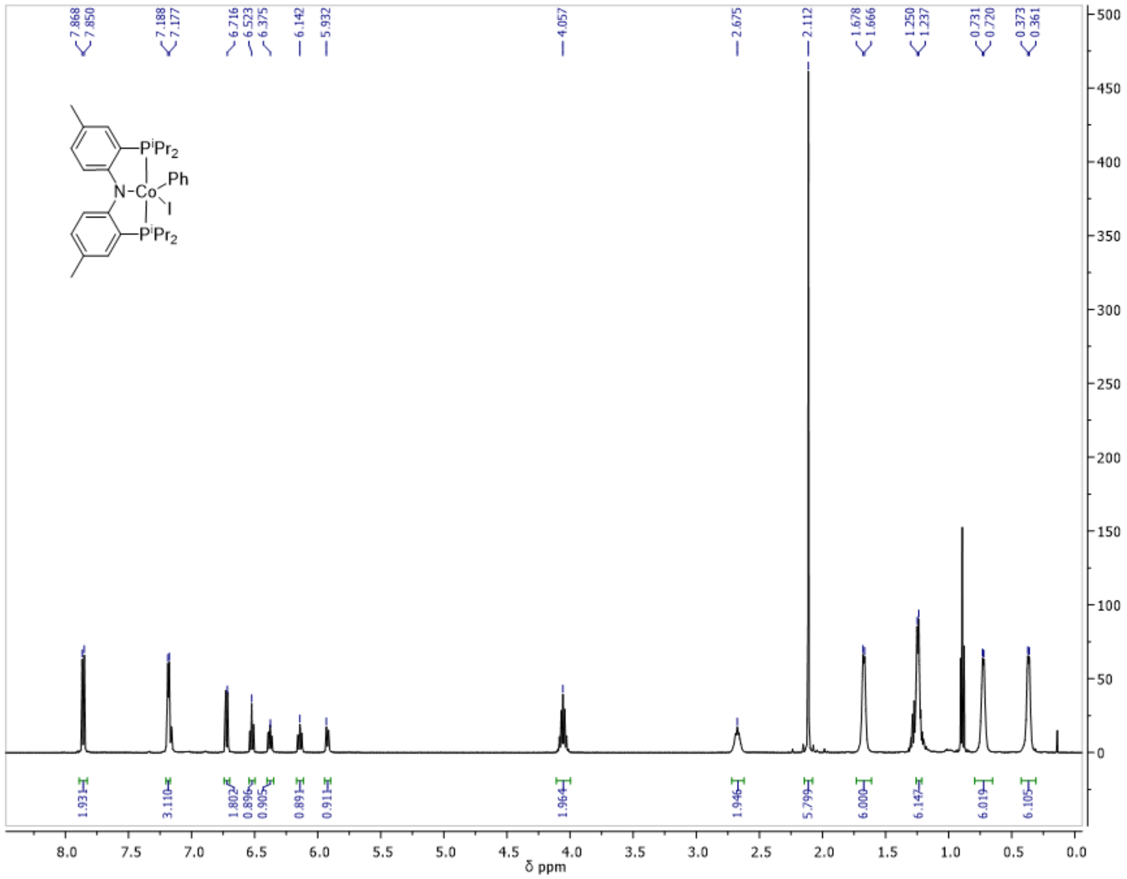

Figure S32. ^{1}H NMR (500 MHz, C_6D_6) spectrum of **5a**. Sample contains residual pentane and silicone grease.

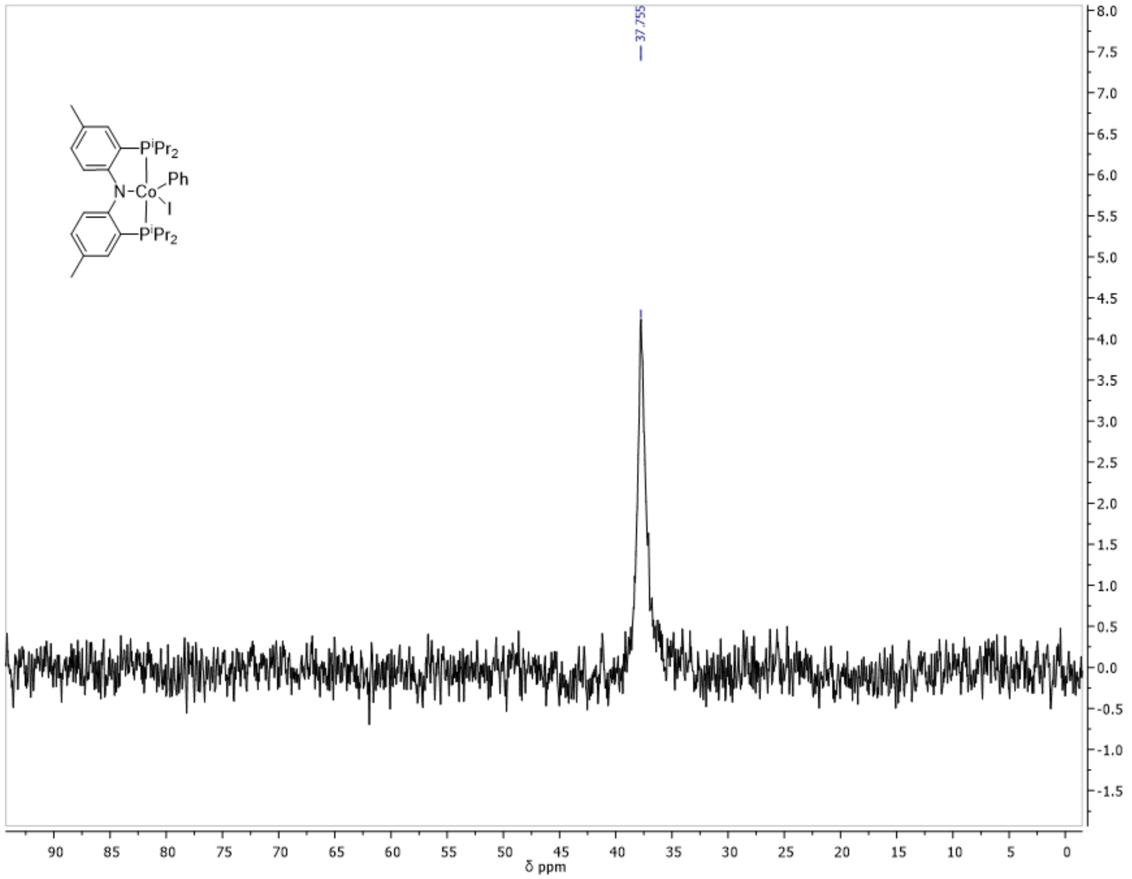

Figure S33. $^{31}P\{^{1}H\}$ NMR (202 MHz, C_6D_6) spectrum of **5a**.

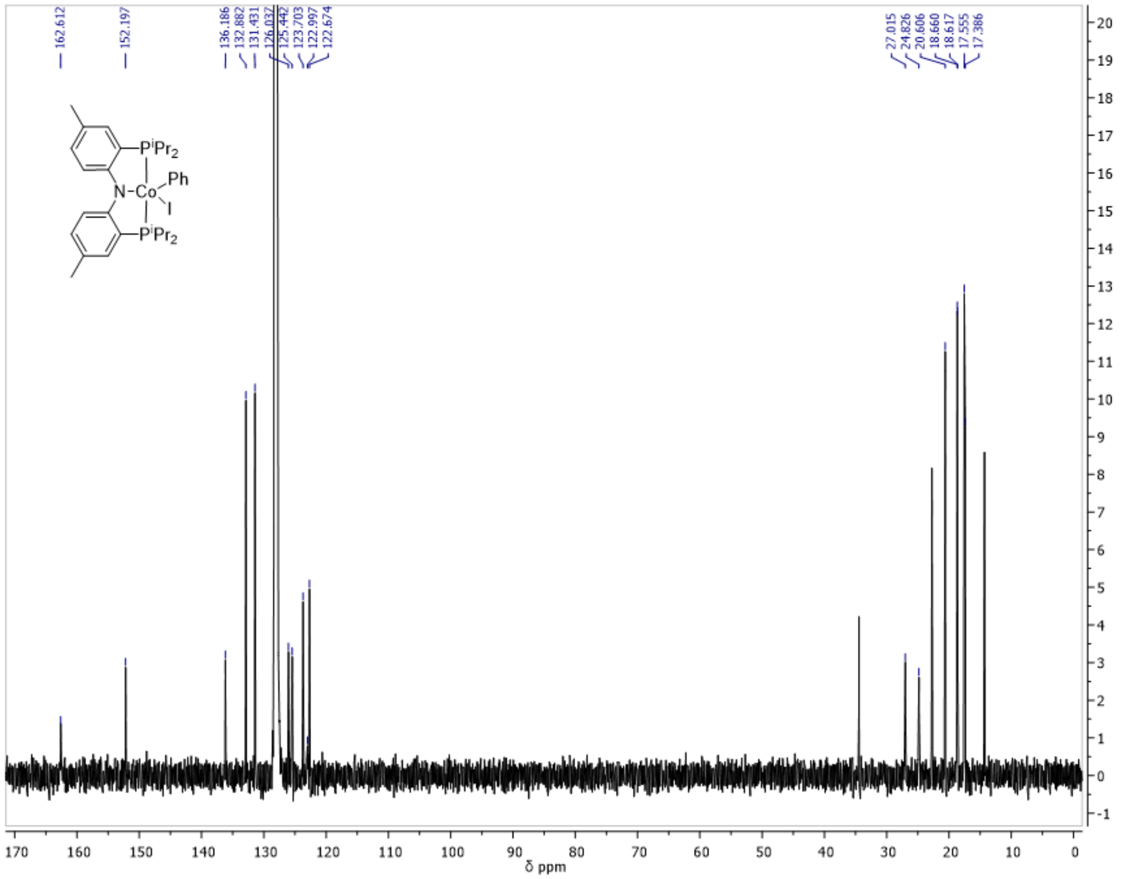

Figure S34. $^{13}C\{^1H\}$ NMR (125 MHz, C_6D_6) spectrum of **5a**. Pentane resonances not picked.

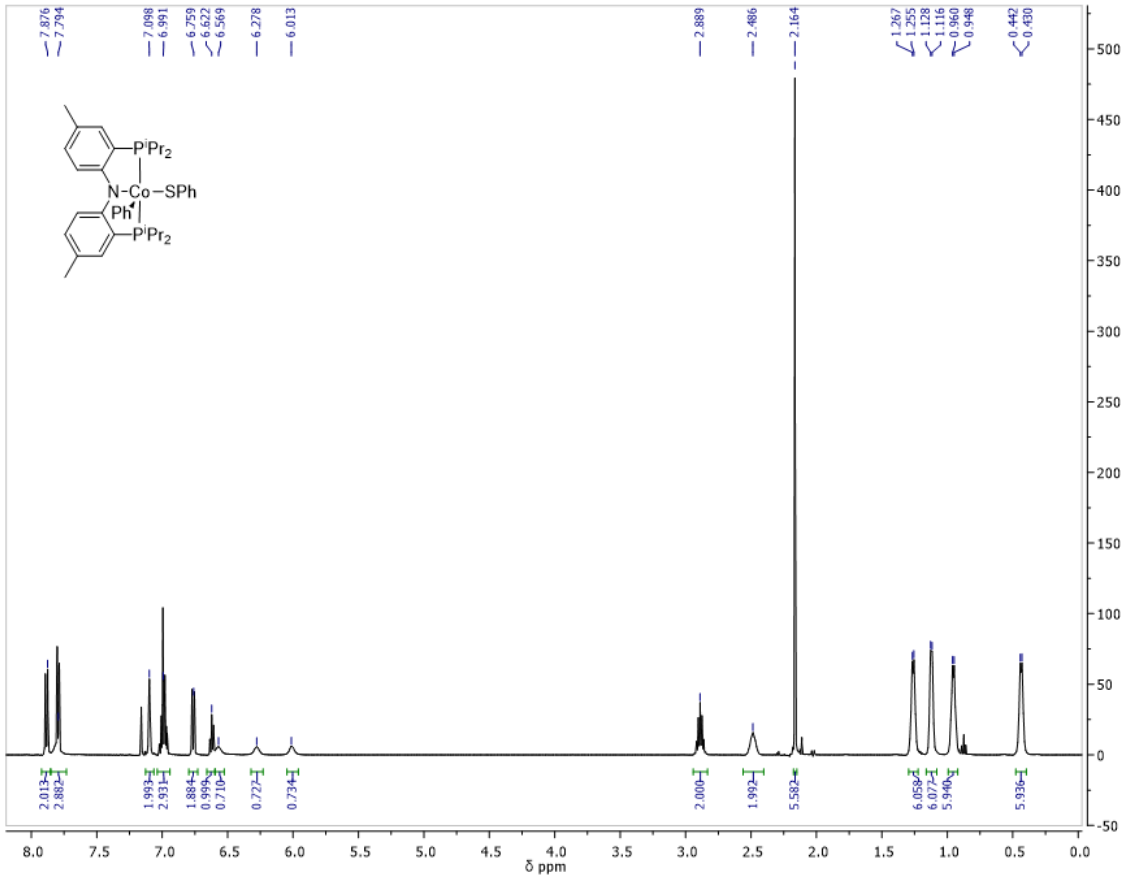

Figure S35. ^{1}H NMR (500 MHz, C_6D_6) spectrum of **6a.** Sample contains residual pentane.

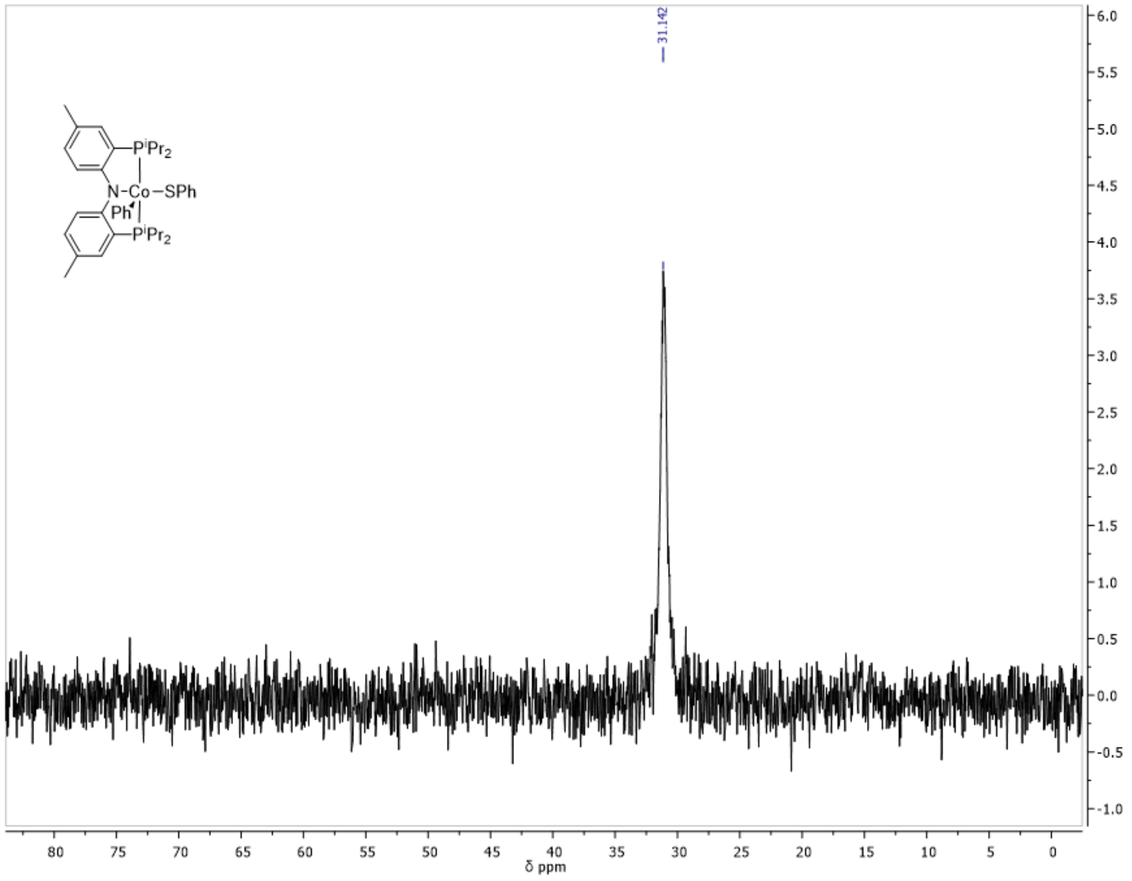

Figure S36. $^{31}P\{^1H\}$ NMR (202 MHz, C_6D_6) spectrum of **6a**.

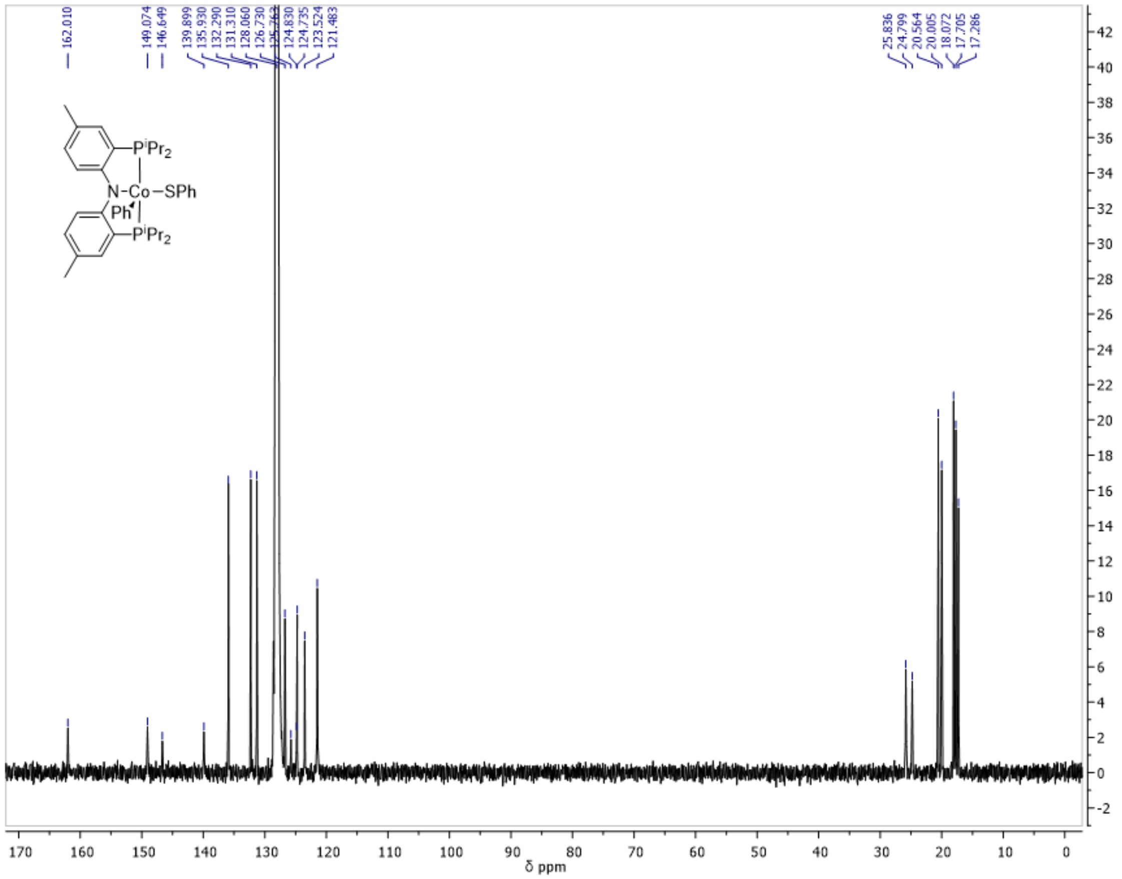

Figure S37. $^{13}C\{^1H\}$ NMR (125 MHz, C_6D_6) spectrum of **6a**.

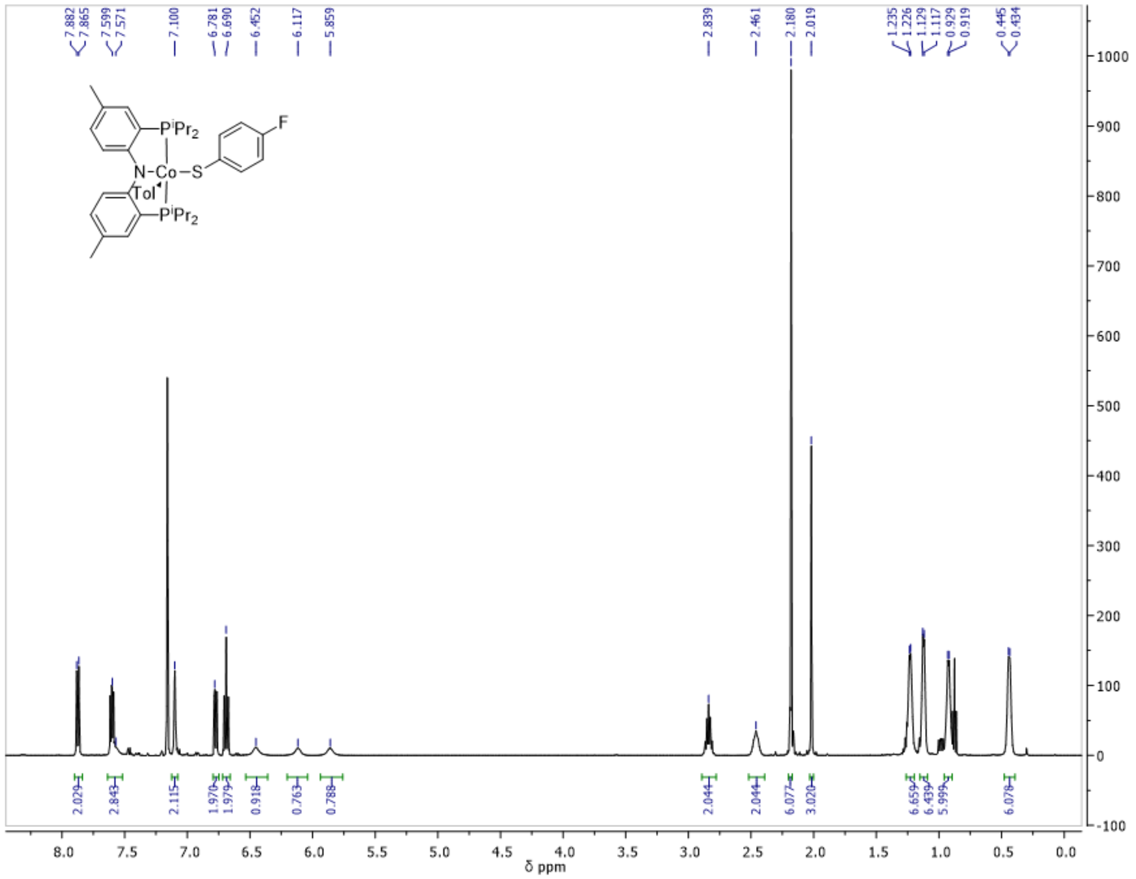

Figure S38. ^{1}H NMR (500 MHz, C_6D_6) spectrum of **6b**. Sample contains residual pentane.

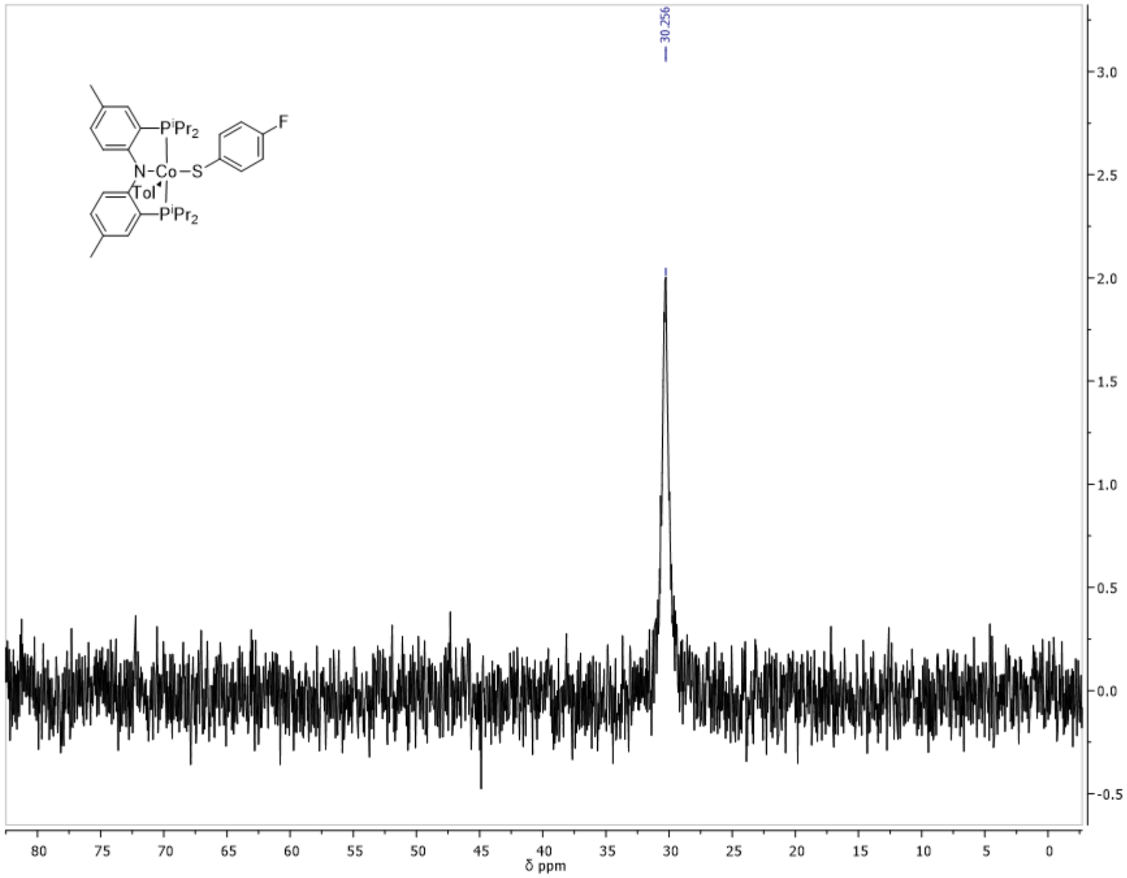

Figure S39. $^{31}P\{^{1}H\}$ NMR (202 MHz, C_6D_6) spectrum of **6b**.

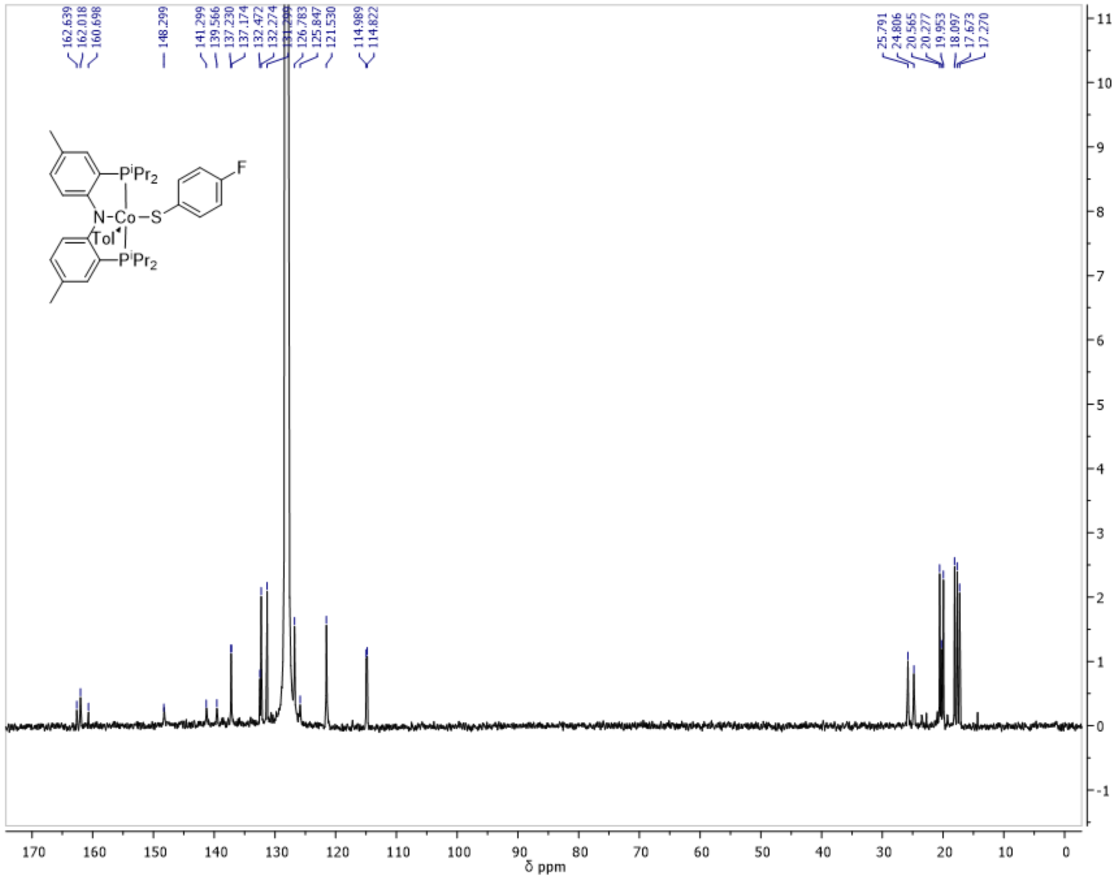

Figure S40. $^{13}C\{^1H\}$ NMR (125 MHz, C_6D_6) spectrum of **6b**.

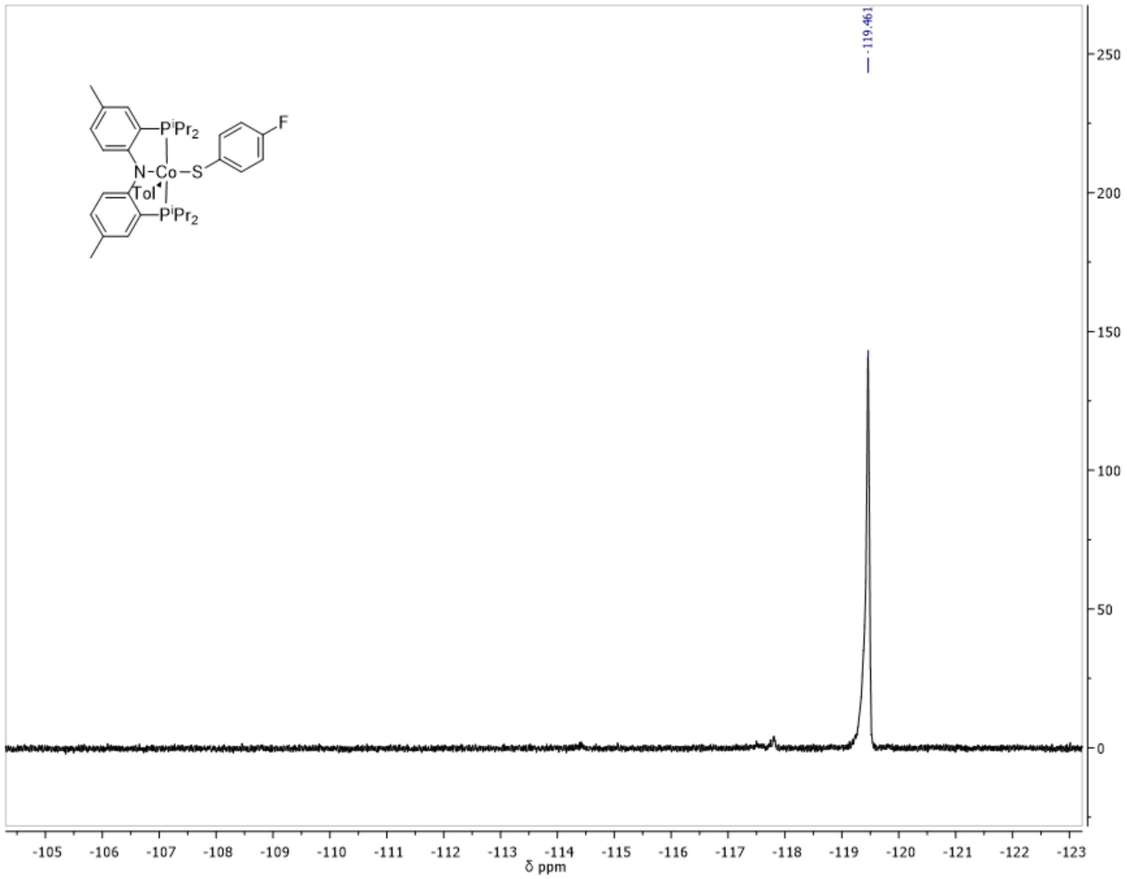

Figure S41. ^{19}F NMR (470 MHz, C_6D_6) spectrum of **6b**.

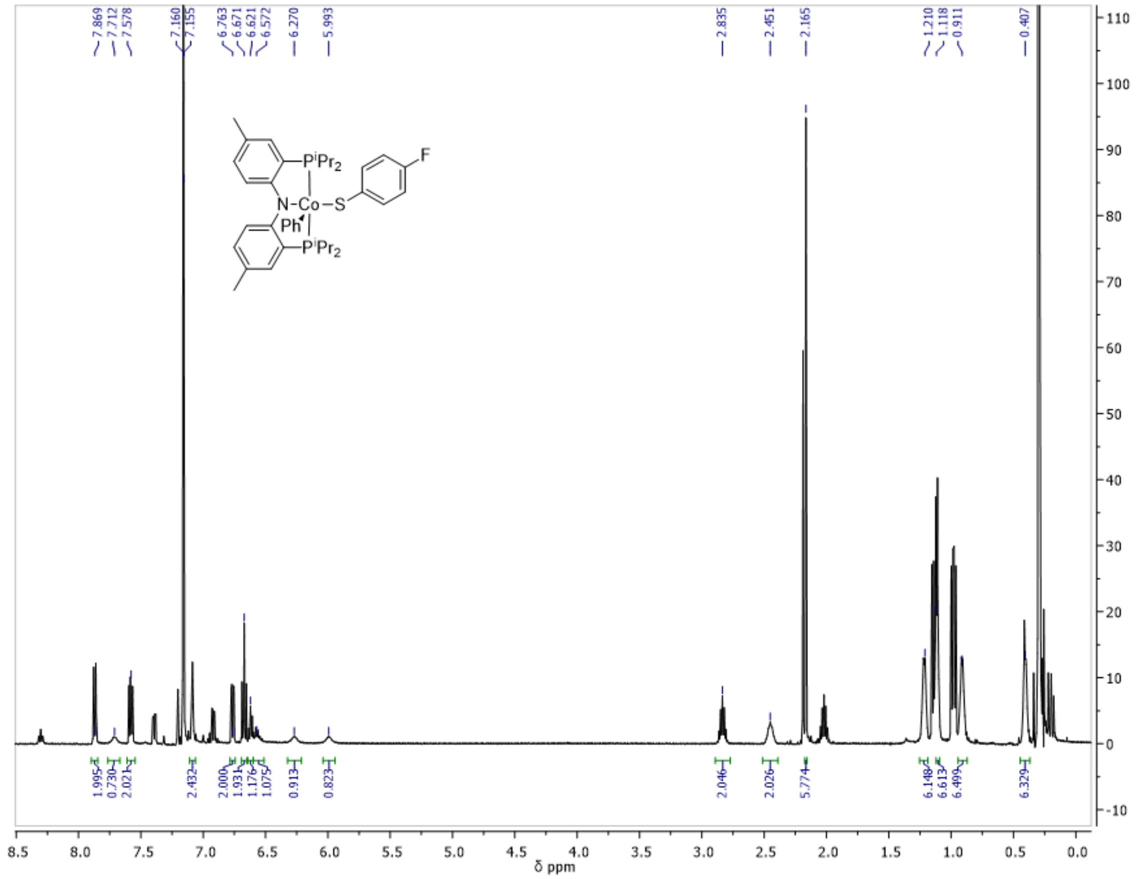

Figure S42. ^{1}H NMR (500 MHz, C_6D_6) spectrum of **6c.** Sample contains residual $^{Me}PN^{H}P^{iPr}$ and trimethylsilyl iodide. Resonances corresponding to the free ligand were not integrated.

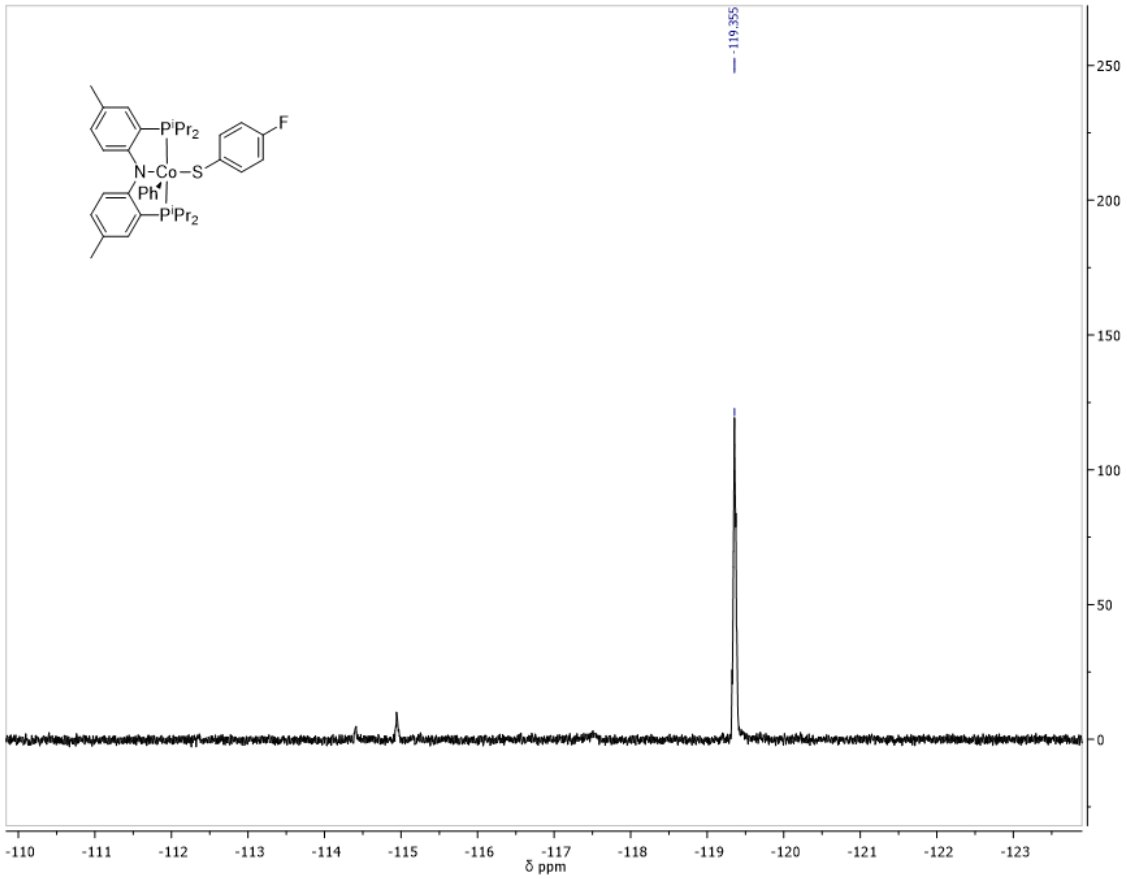

Figure S43. ^{19}F NMR (470 MHz, C_6D_6) spectrum of **6c**.

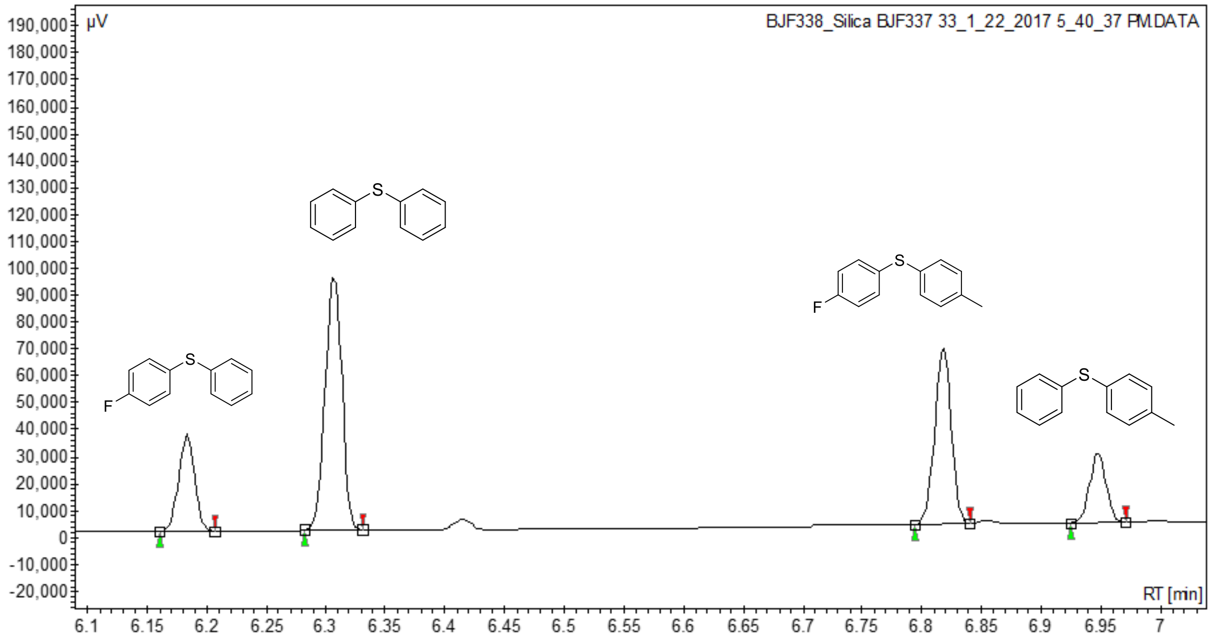

Figure S44.GC-FID of four diaryl sulfides**.**

VII. Computational Details

All computations were carried out with the Gaussian09 program.[15] All of the geometries were fully optimized by B3LYP[16] functional with LANL2DZ pseudopotentials and basis set for Co atom and 6-31G(d) basis set for the other atoms in the gas phase. Wave function of each structure was tested for SCF stability using standard methods[17] and the structure was reoptimized if necessary. In particular, structures **12s**, **13s**, and **7** were calculated to be open-shell singlet, which lies lower in energy than their respective closed-shell singlet counterparts. Harmonic vibrational frequency calculations at the same level of theory were performed to ensure that either a minimum (for intermediates) or a first-order saddle point (for transition states) was obtained, and also to evaluate the zero-point vibrational energy and thermal corrections at 298.15 K The single-point energies and solvent effects were computed with the M06[18] functional using the SDD pseudopotentials and basis set for Co atom and the 6-311+G(d,p) basis set for the other atoms based on the gas-phase optimized structures. The solvation energies were evaluated by a self-consistent reaction field (SCRF) using the SMD implicit solvent model.[19] Unless otherwise specified, the energies reported in this paper are Gibbs free energies under 298.15 K and 1 atm with solvent effect corrections.

VIII. SI References

[1] M. R. Brennan, D. Kim and A. R. Fout, *Chem. Sci.*, 2014, **5**, 4831-4839.

[2] Y. Wakatsuki and H. Yamazaki, *Inorg. Synth.*, 1989, **26**, 190.

[3] L. Fan, B. M. Foxman and O V. Ozerov, *Organometallics*, 2004, **23**, 326-328.

[4] J. C. Demott, N. Bhuvanesh and O. V. Ozerov, *Chem. Sci.*, 2013, **4**, 642-649.

[5] S. D. Timpa, C. J. Pell and O. V. Ozerov, *J. Am. Chem. Soc.*, 2014, **136**, 14772-14779.

[6] H, J. Cristau, B. Chabaud, A. Chêne and H. Christol, *Synthesis*, 1981, **11**, 892-894.

[7] L. J. Farrugia, *J. Appl. Cryst.*, 2012, **45**, 849-854.

[8] POV-Ray – Persistence of Vision Raytracer, http://www.povray.org/ (accessed May 3, 2020).

[9] APEX2 "Program for Data Collection on Area Detectors" BRUKER AXS Inc., 5465 East Cheryl Parkway, Madison, WI 53711-5373 USA.

[10] G. M. Sheldrick, "SADABS (version 2008/1): Program for Absorption Correction of Area Detector Frames, University of Göttingen, Göttingen, Germany, 2008.

[11] G. M. Sheldrick, *Acta Cryst*., 2008, **A64**, 112-122. b) G. M. Sheldrick, *Acta Cryst.*, 2015, **A71**, 3-8. c) Sheldrick, G. M. Sheldrick, *Acta Cryst*., 2015, **C71**, 3-8.

[12] a) A. L. Spek, PLATON - A Multipurpose Crystallographic Tool; Utrecht University, Utrecht, The Netherlands, 2008. b) A. L. Spek, . *J. Appl. Crystallogr.* **2003**, *36*, 7-13. c) A. L. Spek, *Acta. Cryst.*, 2009, **D65**, 148-155.

[13] O. V. Dolomanoc, L. J. Bourhis, R. J. Gildea, J. A. K. Howard and H. Puschmann, *J. Appl. Cryst.*, 2009, **42**, 339-341.

[14] T. Scattolin, E. Senol, G. Yin and Q. Guo, *Angew. Chem., Int. Ed.*, 2018, **57**, 12425-12429.

[15] M. J. Frisch, G. W. Trucks, H. B. Schlegel, G. E. Scuseria, M. A. Robb, J. R. Cheeseman, G. Scalmani, V. Barone, B. Mennucci, G. A. Petersson, H. Nakatsuji, M. Caricato, X. Li, H. P. Hratchian, A. F. Izmaylov, J. Bloino, G. Zheng, J. L. Sonnenberg, M. Hada, M. Ehara, K. Toyota, R. Fukuda, J. Hasegawa, M. Ishida, T. Nakajima, Y. Honda, O. Kitao, H. Nakai, T. Vreven, J. A. Montgomery, Jr., J. E. Peralta, F. Ogliaro, M. Bearpark, J. J. Heyd, E. Brothers, K. N. Kudin, V. N. Staroverov, R. Kobayashi, J. Normand, K. Radhavachari, A. Rendell, J. C. Burant, S. S. Iyengar, J. Somasi, M. Cossi, N. Rega, N. J. Millam, M. Klene, J. E. Knox, J. B. Cross, V. Bakken, C. Adamo, J. Jaramillo, R. Gomperts, R. E. Stratmann, O. Yazyev, A. J. Austin, R. Cammi, C. Pomelli, J. W. Ochterski, R. L. Martin, K. Morokuma, V. G. Zakrzewski, G. A. Voth, P. Salvador, J. J. Dannenberg, S. Dapprich, A. D. Daniels, Ö. Farkas, J. B. Foresman, J. V. Ortiz, J. Cioslowski and D. J. Fox, Gaussian 09 (Revision D.01), Gaussian, Inc., Wallingford, CT, 2009.

[16] a) A. D. Becke, *Phys. Rev. A.*, 1988, **38**, 3098-3100. b) C. Lee, W. Yang and R. G. Parr, *Phys. Rev. B.*, 1988, **37**, 785-789.

[17] R. Seeger and J. A. Pople, *J. Chem. Phys*., 1977, **66**, 3045–3050.

[18] Y. Zhao and D. Truhlar, *Theor. Chem. Acc.*, 2008, **120**, 215-241.

[19] A. V. Marenich, C. J. Cramer and D. G. Truhlar, *J. Phys. Chem. B.*, 2009, **113**, 6378-6796.
